# Comparison of different doses of Tripterygium glycosides treating in IgA vasculitis nephritis: A Bayesian network meta-analysis

**DOI:** 10.1016/j.heliyon.2024.e34329

**Published:** 2024-07-14

**Authors:** Hui Sun, Lijia Liu, Gang Wang, Wei Kong, Yu Zhong, Lan Yi, Yanqin Zou

**Affiliations:** a Department of Nephrology, Jiangsu Province Hospital of Chinese Medicine, Affiliated Hospital of Nanjing University of Chinese Medicine, Nanjing, Jiangsu, China; b No. 1 Clinical Medical College, Nanjing University of Chinese Medicine, Nanjing, Jiangsu, China; c Zou's Nephrology Medicine Intangible Cultural Heritage Inheritance Studio, Nanjing Boda Nephrology Hospital, Nanjing, Jiangsu, China; d Department of Nephrology, Nanjing Hospital of Traditional Chinese Medicine, Nanjing, Jiangsu, China

**Keywords:** Tripterygium glycosides, IgA vasculitis nephritis, Network meta-analysis, Traditional Chinese medicine, Randomized controlled trials

## Abstract

**Background:**

Tripterygium glycosides (TG) is extracted from the roots of *Tripterygium wilfordii Hook F* (Lei gong teng, a traditional Chinese medicine). It is widely used in China to treat immunoglobulin A vasculitis nephritis (IgAVN), which is a common secondary glomerular disease. As there are no guidelines for the rational application of TG, we performed this study to evaluate the efficacy and safety of different doses of TG and to determine the optimal treatment for IgAVN.

**Methods:**

Ten databases were searched from their inception to April 2023 for randomised controlled trials (RCTs) using TG, TG combined with glucocorticoids (GC), or TG combined with traditional Chinese medicine (TCM) to treat IgAVN. A network meta-analysis was performed following the protocol (CRD42023401645).

**Results:**

Forty-four eligible RCTs involving 3402 patients were included. For effective rate, TG 1.5 mg/kg/d (TG1.5) + TCM was ranked as the best intervention, followed by TG 1.0 mg/kg/d (TG1.0) + TCM, TG1.5, TG1.0+GC, TG1.0, TCM, GC, and routine treatment (RT). TG1.0+TCM ranked best in reducing recurrence, followed by TG1.0+GC, GC, TG1.5, and RT. Compared with TG1.0, TG1.0+TCM and TG1.5+TCM effectively reduced liver injury events. Compared with TG1.5, TG1.5+TCM and TG1.0+TCM effectively reduced leukopenia events. No significant differences in the reduction of gastrointestinal events were observed between the interventions. Subgroup analyses explored the effects of the participants’ age. The intervention rankings of the outcomes generally remained consistent. Only a small difference was observed in gastrointestinal events. TCM was the best treatment for reducing gastrointestinal events in paediatric patients.

**Conclusions:**

The results showed a positive correlation between dose and efficacy, whereas no relationship was found between dose and adverse events. TCM can boost the efficacy and reduce adverse events when combined with TG. In conclusion, we consider TG1.5+TCM as the best treatment for IgAVN. However, further research is required to confirm these findings.

## Highlights

• Using Bayesian network Meta-analysis to evaluate different doses of TG treating for IgAVN.
• Exploring the relationship between the dose, combination therapy, safety, and efficacy of TG.
• Finding that increasing TG dose could significantly improve efficacy but not cause more adverse effects.
• Finding that TCM combined with TG can boost efficacy and reduce adverse events.

## Graphical abstract

**Figure:**
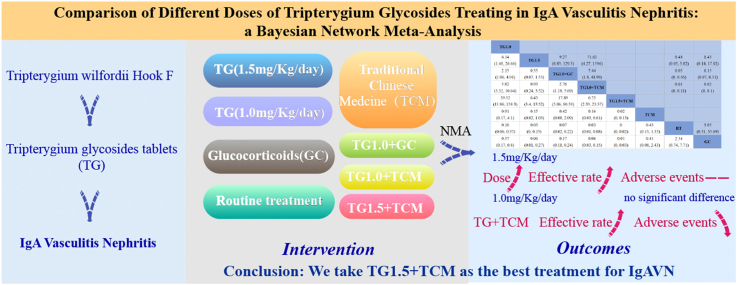

## 1 Introduction

IgA vasculitis (IgAV), also referred to as Henoch-Schönlein purpura, is a systemic disease that affects multiple organs, including the skin, joints, intestines, and kidneys [1]. It is one of the most common forms of vasculitis in children. Most reports show that IgAV nephritis (IgAVN) is diagnosed in 20–60 % of patients with IgAV [2]. The severity of renal injury determines the long-term prognosis of patients with IgAV. In general, IgAVN in children <16 years old is self-limiting. However, a 20-year follow-up study showed that 20 % of children with IgAVN developed chronic kidney disease. Some children are in complete clinical remission for a long time, but chronic renal failure may still occur [3]. IgAVN in adults is not self-limiting. This condition may be more severe and is likely to relapse.

Currently, management of IgAVN remains controversial. According to the Kidney Disease Improving Global Outcomes Guidelines [4], treatment for IgAVN includes angiotensin-converting enzyme inhibitors (ACEI), angiotensin II receptor blockers (ARB), glucocorticoids (GC), and immunosuppressants. Many studies indicate that ACEI/ARB are the preferred drugs for treating IgAVN and should be used as soon as possible to reduce urinary protein levels [5,6]. However, some patients cannot tolerate adequate use of ACEI and ARB because of hypotension. If patients fail to respond to this treatment, GC and immunosuppressant therapy should be considered. GC and immunosuppressants may cause more adverse events, which requires clinicians to weigh their advantages and disadvantages to make the right decision.

*Tripterygium wilfordii Hook F* (Lei gong teng, a traditional Chinese medicine [TCM]) is a member of Tripterygium of the Celastraceae family (http://www.theplantlist.org/). According to TCM theory, Lei gong teng can dispel wind and dampness, relieve swelling and pain, and promote blood circulation to dredge collaterals. Tripterygium glycosides (TG) were extracted from *Tripterygium wilfordii Hook F* root and prepared into tablets. Due to its immunosuppressive effect, it is widely used to treat various types of glomerulonephritis in China, including IgAVN, IgA nephropathy, and idiopathic membranous nephropathy [7]. A meta-analysis of IgAVN in children, including 16 randomised controlled trials (RCTs), reported that the total effective rate (efficacy defined as a 30 % decrease in haematuria and albuminuria compared to baseline values) was better in the TG group than in the GC group (RR 1.26, 95%CI 1.04, 1.51, P = 0.02) [8]. Another meta-analysis of IgAVN in adults, including 12 RCTs, reported that the total effective rate was better in the TG + GC group than in the GC group (RR 1.20, 95%CI 1.13, 1.27, P < 0.00001) and ACEI group (RR 1.20, 95%CI 1.13, 1.27, P < 0.00001) [9]. This shows that TG have a better effect than GC on children and adults.

Currently, the use of TG in China is controversial. Although it has immunosuppressive effects, it also has many adverse effects such as liver injury and damage to the haematological and reproductive systems. It is difficult for clinicians to perform rational clinical applications because there are no precise regulations regarding dosage use. In several randomised controlled clinical studies in China, TG was available at various doses, including 2.0 mg/kg/d, 1.5 mg/kg/d, and 1.0 mg/kg/d, with the latter two being the most commonly used doses. An increasing number of RCTs have been published in recent years. Therefore, given the above-mentioned controversial issues and the latest relevant research, we conducted a network meta-analysis (NMA) to estimate the efficacy and safety of different doses of TG and to further identify better treatments for patients with IgAVN.

## 2 Materials and methods

### 2.1 Study design

The NMA was registered with the PROSPERO database (CRD42023401645). This study was conducted following a pre-specified protocol [10] and the Preferred Reporting Items for Systematic Reviews and Meta-Analyses (PRISMA) for network meta-analyses [11] (Supplementary file S1).

### 2.2 Search strategy

The following databases were searched: PubMed, Web of Science (WOS), Embase, Cochrane Library, Chinese Biomedical Literature Database (CBM), China National Knowledge Infrastructure (CNKI), Wanfang Database, VIP Database, International Clinical Trials Registry Platform (ICTRP), and ClinicalTrials.gov trial registry. All databases were searched from their inception until April 2023. A detailed search strategy is available in Supplementary File S2. We also reviewed the relevant article references to determine whether a study was omitted.

### 2.3 Inclusion and exclusion criteria

Eligible studies were selected to meet the following criteria: (1) RCTs that compared TG therapies with GC, TCM, or routine treatment (RT) for treating IgAVN; and (2) outcomes that included one of the following: effective rate (defined as a 25%–50 % decrease in haematuria and albuminuria compared with baseline values), recurrence rate, liver injury events, leukopenia events, and gastrointestinal events. The exclusion criteria were: (1) duplicate publications; (2) studies with incomplete data; (3) studies with no specific description of treatment, such as those that did not specify the dosage of medication; and (4) RT containing other essential treatments besides anti-infection, anti-allergy, ACEI, and ARB.

### 2.4 Data extraction and quality assessment

Two reviewers (Sun and Liu) searched the same databases and independently evaluated all the eligible articles for inclusion. Duplicates were eliminated using EndNote 20 software. Data from the included studies were independently extracted by two reviewers (Sun and Liu) using Microsoft Excel 16.0. Data of interest included authors, year of publication, sex, age, disease course, sample size, diagnostic criteria, treatment and control group interventions, outcome-related data, duration of treatment, adverse events, and information needed to evaluate the risk of bias. Disagreements were resolved through discussion with a third reviewer (Wang).

Two reviewers (Sun and Liu) independently assessed the methodological quality of the included studies using the Cochrane risk of bias tool. This tool assesses selection, performance, detection, attrition, reporting, and other sources of bias. Each project's risk assessment results were classified as low, unclear, or high risk. Disagreements were analysed by Yi.

### 2.5 Statistical analysis

We used RevMan 5.4, Stata 17.0, and R 4.2.2 software to perform the analyses. Odds ratios (OR) with 95 % confidence intervals (CI) were evaluated for dichotomous data. Pairwise meta-analyses were conducted using RevMan software. Heterogeneity was analysed using the I^2^ statistical test. A fixed-effects model was used because no heterogeneity was observed (I<50 %). The “networkplot” function of Stata was used to generate network plots. The NMA was conducted in a Bayesian framework through Markov Chain Monte Carlo (MCMC) simulation using the “gemtc” R package of R software. A random-effects network meta-analysis model was used to synthesise the study effect sizes. Convergence was assessed using the Brooks-Gelman-Rubin plot method. We assessed heterogeneity for all comparisons from the NMA models using the I^2^ statistical test in the “gemtc” R package. The node splitting method was used to examine the inconsistency between the direct and indirect comparisons if a loop connecting three or more arms existed. Inconsistency was deemed non-significant when the p-value was >0.05, when comparing direct and indirect effects. The rank probabilities of the interventions were evaluated using a ranking cumulative probability plot and surface under the cumulative ranking curve (SUCRA), in which a SUCRA value from 1 to 0 indicated that the treatment effects decreased. Cluster rank plots were generated based on SUCRA values to evaluate the efficacy and safety of the different interventions in the two dimensions. To test the robustness of the results, we performed subgroup analyses by age, given that this variable might have affected the participants’ responses to treatment. We divided the RCTs with participants under 18 years old into a child group and those older than 18 years into an adult group. Publication bias was evaluated using funnel plots using Stata software. In all statistical tests, a p-value <0.05 was considered statistically significant.

## 3 Results

### 3.1 Study selection

**Fig. 1:**
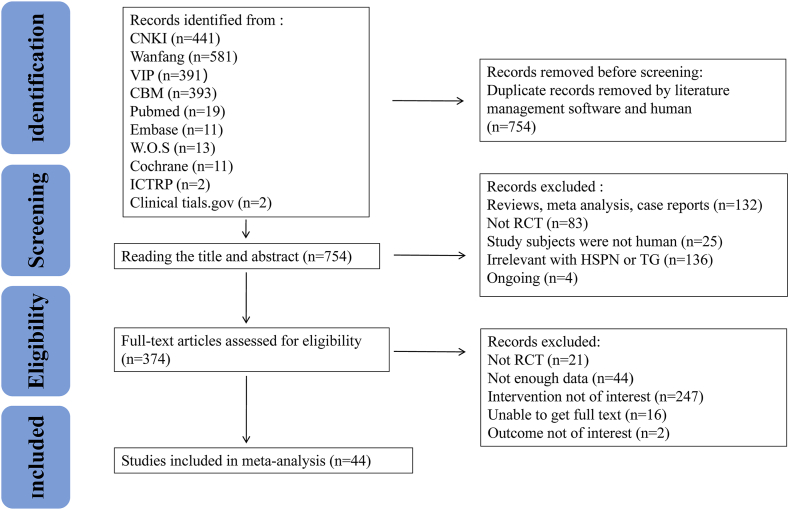
Study selection flowchart.

On the initial search, 1868 articles were included. After removing duplicates, 754 articles were retained. A total of 376 articles were excluded after reading titles and abstracts. After reading the full text, 374 records were excluded. Finally, 44 studies with 3402 participants were included in this meta-analysis. The detailed selection process is illustrated in Fig. 1.

### 3.2 Study characteristics

The characteristics of the included studies are shown in Supplementary Table S3.1, and references to the studies are shown in Supplementary file S3.2. The 44 enrolled studies were published between 1994 and 2022. All the patients were diagnosed with IgAVN. The definition of IgAVN includes renal impairment manifestations during the IgAV, such as proteinuria and haematuria (primarily within six months). The number of participants in each study ranged from 30 to 127. Approximately 56.68 % of the participants were male. Of the 44 RCTs, 30 worked on children, 11 worked on adults, and 3 did not limit the age of participants.

**Fig. 2:**
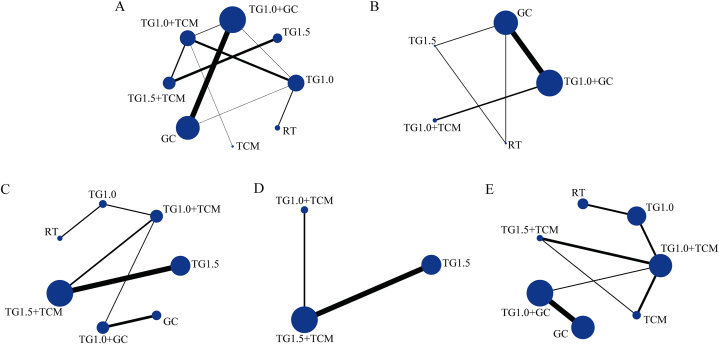
Network plots for each outcome. A. Effective rate; B, Recurrence rate; C, Liver Injury Events; D, Leukopenia events; E, Gastrointestinal events. Abbreviations: TG1.0, Tripterygium Glycosides (dose: 1 mg/kg/day); TG1.5, Tripterygium Glycosides (dose: 1.5 mg/kg/day); TCM, Traditional Chinese Medicine; GC, Glucocorticoids; RT, Routine treatment.

Five studies compared the effects of TG and RT, 20 studies compared the effects of TG/TG + GC and GC, 16 studies compared TG + TCM and TG/TCM, 2 studies compared TG + TCM and TG + GC, and 3 studies compared different doses of TG. The pairwise meta-analysis (PMA) results are summarised in Table 1, Table 2. Network plots of the intervention comparisons for each outcome are shown in Fig. 2. Each node represented an intervention. The node size represents the number of patients receiving this intervention, and the thickness of the line between the two nodes represents the number of studies comparing the two interventions.

**Table 1:** Results of PMA (comparison of effective rate).

Comparison	Number of Studies	Number of Participants	I^2^ (%)	p (I^2^)	Effect Estimate (OR, 95 % CI)	p (OR)
TG1.0 VS RT	3	278	0	0.69	5.95 [2.52, 14.07]	<0.0001
TG1.0 VS GC	1	59	–	–	2.85 [0.99, 8.21]	0.05
TG1.0+GC VS GC	18	1413	0	1.00	5.55[3.85,8.00]	<0.00001
TG1.0+TCM VS TG1.0	7	486	0	0.83	4.5[2.51,8.07]	<0.00001
TG1.0+TCM VS TCM	2	124	0	0.81	5.03[1.76,14.44]	0.003
TG1.0+TCM VS TG1.0+GC	2	108	0	0.34	4.01[1.49,10.82]	0.006
TG1.5+TCM VS TG1.0+TCM	3	164	0	0.76	4.35[1.60,11.79]	0.004
TG1.5+TCM VS TG1.5	7	517	0	0.93	5.71[3.01,10.84]	<0.00001
TG1.5+TCM VS TCM	1	24	–	–	34.09[1.64,707.92]	0.02

Abbreviations: **TG1.0,** Tripterygium Glycosides (dose: 1 mg/kg/day); **TG1.5,** Tripterygium Glycosides (dose: 1.5 mg/kg/day); **TCM,** traditional Chinese medicine; **GC,** glucocorticoid; **RT,** routine treatment; **OR,** odds ratio; **CI,** confidence interval.

**Table 2:** Results of PMA (comparison on secondary outcomes (NO. of studies >1)).

Comparison	Number of Studies	Number of Participants	I^2^ (%)	p (I^2^)	Effect Estimate (OR, 95 % CI)	p (OR)
Recurrence rate						
TG1.0+GC VS GC	8	649	0	0.94	0.17 [0.09, 0.32]	<0.00001
TG1.0+TCM VS TG1.0+GC	2	108	0	0.95	0.15 [0.04, 0.58]	0.006
Liver injury events						
TG1.5+TCM VS TG1.5	5	401	0	0.95	0.29 [0.09, 0.98]	0.05
TG1.5+TCM VS TG1.0+TCM	2	140	0	0.33	1.43 [0.44, 4.68]	0.55
TG1.0+GC VS GC	3	186	36	0.21	0.62 [0.20, 1.92]	0.40
Gastrointestinal events						
TG1.0+GC VS GC	6	436	58	0.04	−0.01 [-0.06, 0.04]	0.59
TG1.0+TCM VS TCM	2	124	22	0.26	0.03 [-0.05, 0.11]	0.43
TG1.5+TCM VS TG1.0+TCM	2	84	0	0.39	0.02 [-0.06, 0.11]	0.60
TG1.0 VS RT	2	196	10	0.29	−0.05 [-0.11, 0.01]	0.08
TG1.0+TCM VS TG1.0	2	160	0	0.40	0.00 [-0.06, 0.06]	1.00
Leukopenia events						
TG1.5+TCM VS TG1.5	5	401	0	0.99	0.19 [0.05, 0.74]	0.02
TG1.5+TCM VS TG1.0+TCM	2	140	0	0.77	2.01 [0.51, 7.94]	0.32

Abbreviations: **TG1.0,** Tripterygium Glycosides (dose: 1 mg/kg/day); **TG1.5,** Tripterygium Glycosides (dose: 1.5 mg/kg/day); **TCM,** traditional Chinese medicine; **GC,** glucocorticoid; **RT,** routine treatment; **OR,** odds ratio; **CI,** confidence interval.

**Table 3:** The league table of two outcomes.

TG1.0							
6.14 (1.48, 26.66)	TG1.5	9.27 (0.83, 129.3)	71.62 (4.27, 1596)			0.48 (0.05, 3.82)	1.43 (0.14, 17.92)
2.15 (1.06, 4.91)	0.35 (0.07, 1.53)	TG1.0+GC	7.44 (1.8, 41.99)			0.05 (0, 0.56)	0.15 (0.07, 0.31)
5.82 (3.32, 10.64)	0.93 (0.24, 3.52)	2.76 (1.18, 5.69)	TG1.0+TCM			0.01 (0, 0.11)	0.02 (0, 0.1)
39.52 (11.86, 158.3)	6.43 (3.4, 13.52)	17.89 (5.06, 80.59)	6.75 (2.33, 23.57)	TG1.5+TCM			
0.91 (0.17, 4.1)	0.15 (0.02, 1.03)	0.42 (0.08, 2.09)	0.16 (0.03, 0.61)	0.02 (0, 0.13)	TCM		
0.16 (0.06, 0.37)	0.03 (0, 0.15)	0.07 (0.02, 0.22)	0.03 (0.01, 0.08)	0 (0, 0.02)	0.43 (0.13, 1.35)	RT	3.05 (0.31, 35.69)
0.37 (0.17, 0.8)	0.06 (0.01, 0.27)	0.17 (0.11, 0.24)	0.06 (0.03, 0.15)	0.01 (0, 0.03)	0.41 (0.08, 2.43)	2.34 (0.74, 7.71)	GC

Note: A summary of the NMA results for the efficacy rate (bottom left) and recurrence rate (top right corner) is shown in Table 3. The row-defining treatment was compared with the column-defining treatment, and the relative effects were measured as risk ratios along with 95%CI. Abbreviations: **TG1.0,** Tripterygium Glycosides (dose: 1 mg/kg/day); **TG1.5,** Tripterygium Glycosides (dose: 1.5 mg/kg/day); **TCM,** traditional Chinese medicine; **GC,** glucocorticoid; **RT,** routine treatment.

### 3.3 Risk of bias

A summary of the risk of bias assessment is shown in Supplementary Fig. S4.1, and the assessment of each study is shown in Supplementary Fig. S4.2. For selection bias (random sequence generation), one study was at a high risk of bias because the patients were grouped based on enrolment time. Selection bias (allocation concealment), performance bias, detection bias, and reporting bias were evaluated as “unclear risks " because all studies did not obtain information on allocation concealment, blinding, prescribed schemes, or protocols. Regarding attrition bias and other bias, there was no data omission and other obvious bias in all studies, so they were evaluated as “low risk."

### 3.4 Inconsistency, heterogeneity

The node-splitting analysis results showed no significant inconsistency between the direct and indirect comparisons (Supplementary Fig. S5.1.1, Fig. S5.5.1). Study heterogeneity was assessed using the statistic of I^2^. Moreover, there was no evidence of differences in heterogeneity between the direct and indirect evidence (Supplementary Fig. S5.1.2, Fig. S5.2.1, Fig. S5.3.1, Fig. S5.4.1, Fig. S5.5.2).

### 3.5 Outcomes of interest

#### 3.5.1 Effective rate

**Fig. 3:**
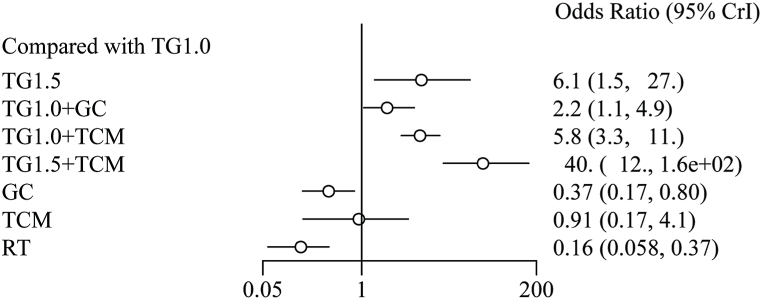
The forest plot of effective rate. Abbreviations: TG1.0, Tripterygium Glycosides (dose: 1 mg/kg/day); TG1.5, Tripterygium Glycosides (dose: 1.5 mg/kg/day); TCM, traditional Chinese medicine; GC, glucocorticoid; RT, routine treatment; CrI, confidence interval.

A total of 42 studies reported information on the efficacy rate, which included 3173 individuals who were pooled to perform the NMA. A network diagram of the treatments is shown in Fig. 2A. The pooled odds ratios (OR) and 95 % confidence intervals (CI) corresponding to the PMA are shown in Table 1. Direct comparisons suggested that, compared to TG1.0, TG1.0+TCM (OR 4.50, Crl [2.51, 8.07], I^2^ = 0 %) had a higher efficacy rate (p < 0.001). Bayesian NMA (Fig. 3) also proved that compared to TG1.0, TG1.0+TCM (OR 5.82, Crl [3.32, 10.64]) could significantly improve efficacy. TG1.0+GC (OR 2.15, Crl [1.06, 4.91]), TG1.5 (OR 6.14, Crl [1.48, 26.66]) and TG1.5+TCM (OR 39.52, Crl [11.86, 158.3]) showed similar effects. TCM (OR: 0.91, Crl [0.17, 4.1]) showed no significant effect. However, GC (OR 0.37, Crl [0.17, 0.8]) and RT (OR 0.16, Crl [0.06, 0.37]) were lower than TG 1.0 in the efficacy rate. The cumulative probability plot (Fig. 5A) shows the rank for all these interventions. TG1.5+TCM was ranked as the best treatment, with the highest probability of improving efficacy, followed by TG1.0+TCM, TG1.5, TG1.0+GC, TG1.0, TCM, GC, and RT (Table 4).

**Table 4:** The surface under the cumulative ranking curve (SUCRA) of each outcome.

Rank	Effective rate		Recurrence rate	
	Treatment	S–V	Treatment	S–V
1	TG1.5+TCM	1.000	TG1.0+TCM	0.998
2	TG1.0+TCM	0.782	TG1.0+GC	0.742
3	TG1.5	0.772	GC	0.371
4	TG1.0+GC	0.563	TG1.5	0.292
5	TG1.0	0.364	RT	0.097
6	TCM	0.351		
7	GC	0.154		
8	RT	0.014		

Rank	Liver injury events		Gastrointestinal events		leukopenia events	
	Treatment	S–V	Treatment	S–V	Treatment	S–V
1	TG1.0+TCM	0.820	TCM	0.802	TG1.0+TCM	0.914
2	RT	0.709	TG1.0	0.614	TG1.5+TCM	0.586
3	TG1.5+TCM	0.676	TG1.5+TCM	0.605	TG1.5	0.000(5.250E-05)
4	TG1.0+GC	0.482	TG1.0+TCM	0.600		
5	GC	0.422	TG1.0+GC	0.299		
6	TG1.5	0.345	GC	0.296		
7	TG1.0	0.046	RT	0.285		

Abbreviations: **TG1.0,** Tripterygium Glycosides (dose: 1 mg/kg/day); **TG1.5,** Tripterygium Glycosides (dose: 1.5 mg/kg/day); **TCM,** traditional Chinese medicine; **GC,** glucocorticoid; **RT,** routine treatment; **S–V,** SUCRA value.

Our subgroup analysis of the children with IgAVN revealed consistent results (Supplementary file S5.1.3). In the adult group, only two interventions were included: TG1.0+GC and GC. The direct comparison suggested that compared to GC, TG + GC (OR 1.23, Crl [1.17, 1.30]) had a higher effective rate (Supplementary file S5.1.4).

#### 3.5.2 Recurrence rate

**Fig. 4:**
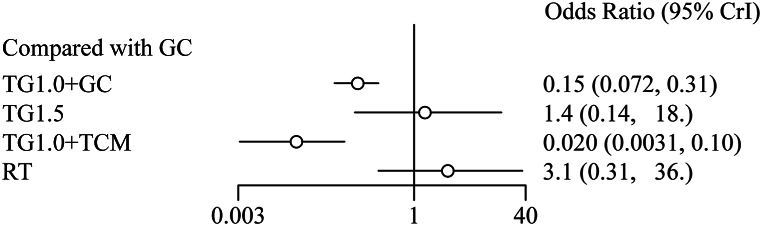
The forest plot of recurrence rate. Abbreviations: TG1.0, Tripterygium Glycosides (dose: 1 mg/kg/day); TG1.5, Tripterygium Glycosides (dose: 1.5 mg/kg/day); TCM, traditional Chinese medicine; GC, glucocorticoid; RT, routine treatment; CrI, confidence interval.

**Fig. 5:**
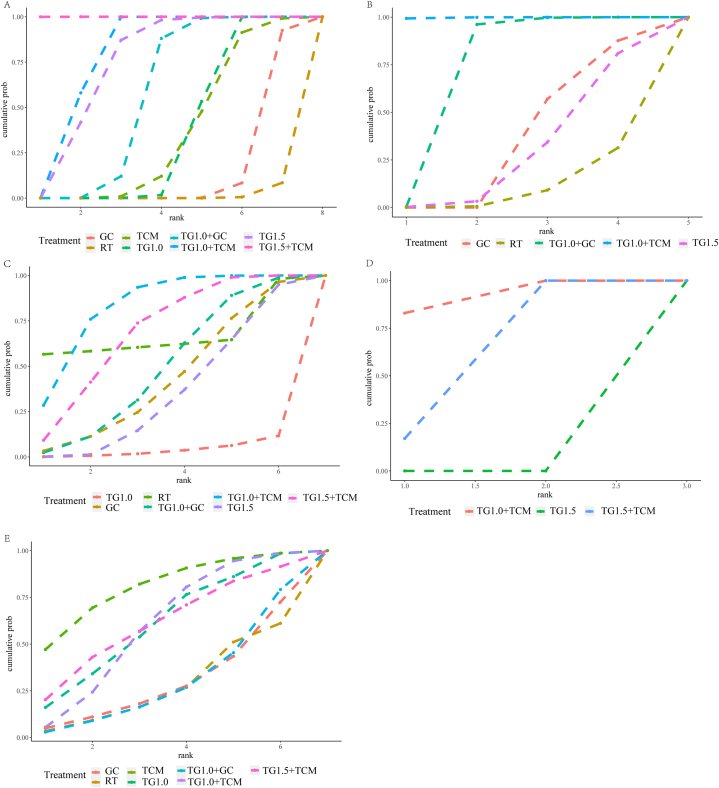
The ranking cumulative probability plot of each outcome. A. Effective rate; B, Recurrence rate; C, Liver Injury Events; D, Leukopenia events; E, Gastrointestinal events. Abbreviations: **TG1.0,** Tripterygium Glycosides (dose: 1 mg/kg/day); **TG1.5,** Tripterygium Glycosides (dose: 1.5 mg/kg/day); **TCM,** Traditional Chinese Medicine; **GC,** Glucocorticoids; **RT,** Routine treatment.

Eleven studies reported information on the recurrence rate, and 821 individuals were available for NMA. A network diagram of the treatments is shown in Fig. 2B. The direct comparisons (Table 2) suggested that compared to GC, TG1.0+GC (OR 0.17, Crl [0.09, 0.32], I^2^ = 0 %) significantly reduced the recurrence rate (p < 0.001). TG1.5 (OR1.33, Crl [0.18, 10.12]) did not show significant effects. Bayesian NMA (Fig. 4) proved that compared to GC, TG1.0+GC (OR 0.15, Crl [0.07, 0.31]) reduced the recurrence rate. TG1.0+TCM (OR 0.02, Crl [0, 0.1]) had a similar effect. TG1.5 (OR 1.43, Crl [0.14, 17.92]) and RT (OR 3.05, Crl [0.31,35.69]) did not show better effects in reducing recurrence. TG1.0+TCM was ranked as the best treatment with the highest probability of reducing the recurrence rate, followed by TG1.0+GC, GC, TG1.5, and RT (Fig. 5B–Table 4).

Our subgroup analysis results indicated that compared with GC, TG1.0+GC was significantly more effective (OR 0.19, Crl [0.11, 0.34]) in reducing recurrence in adults with IgAVN (Supplementary file S5.2.3). The RCTs examining children with IgAVN did not report this outcome.

#### 3.5.3 Liver injury events

Thirteen studies involving 937 individuals reported on liver injury. A network diagram of the treatments is presented in Fig. 2C. The PMA suggested that compared to TG1.0, TG1.0+TCM (OR 0.13, Crl [0.01, 2.58]) did not achieve a significant effect (P > 0.05). The NMA results (Supplementary Fig. S5.3.3) showed that compared with TG1.0, TG1.0+TCM (OR 0, Crl [0, 0.23]) effectively reduced the incidence of liver injury events. TG1.5+TCM (OR 0, Crl [0,0.7]) (indirect comparison) and RT (OR 0, Crl [0, 0.65]) also had similar effects. However, TG1.5 (OR 0, Crl [0, 9.02]), TG1.0+GC (OR 0, Crl [0,4.03]) and GC (OR 0, Crl [0,6.66]) could not reduce the occurrence of liver injury. TG1.0+TCM was ranked as the best treatment with the highest probability of reducing the incidence of liver dysfunction, followed by RT, TG1.5+TCM, TG1.0+GC, GC, TG1.5, and TG1.0 (Fig. 5C–Table 4).

Our subgroup analysis of children with IgAVN revealed similar results; TG1.0+TCM was the best-ranked treatment for reducing liver dysfunction events (Supplementary file S5.3.5). The RCTs examining adults with IgAVN did not report this outcome.

#### 3.5.4 Leukopenia events

Seven studies, involving 541 individuals, reported leukopenia. A network diagram of the treatments is presented in Fig. 2D. PMA (Table 2) suggested that compared to TG1.5, TG1.5+TCM (OR 0.19, Crl [0.05,0.74], I^2^ = 0 %) reduced the incidence of leukopenia (p = 0.02). The NMA results (Supplementary Fig. S5.4.3) showed that, compared to TG1.5, TG1.5+TCM (OR 0, Crl [0, 0.02]) effectively reduced the incidence of leukopenia events. TG1.0+TCM (OR 0, Crl [0, 0.01]) had a similar effect. TG1.0+TCM was ranked as the best treatment with the highest probability of reducing the incidence of leukopenia, followed by TG1.5+TCM and TG1.5 (Fig. 5D–Table 4). The seven RCTs were all working on children with IgAVN. There was no need to perform subgroup analysis based on age.

#### 3.5.5 Gastrointestinal events

Fourteen studies involving 1086 individuals reported gastrointestinal events. A network diagram of the treatments is presented in Fig. 2E. PMA (Table 2) suggested that compared to TG1.0, TG1.0+TCM (OR 1, Crl [0.18, 5.62], I^2^ = 0 %) did not achieve a significant effect (P > 0.05). The NMA results (Supplementary Fig. S5.5.4) showed that, compared to TG1.0, TG1.0+TCM (OR 1.02, Crl [0.08, 13.93]) did not effectively reduce the adverse effects of gastrointestinal events. No significant differences were observed between TG1.0, TG1.5+TCM, TG1.0, TG1.0 and TG1.0+GC, TG1.0, and GC. TCM was ranked as the best treatment for reducing gastrointestinal events, followed by TG1.0, TG1.5+TCM, TG1.0+TCM, TG1.0+GC, GC, and RT (Fig. 5E–Table 4). However, according to our subgroup analysis, TCM showed significant benefits compared with other interventions in children with IgAVN (Supplementary file S5.5.6). The RCTs examining adults with IgAVN did not report this outcome.

#### 3.5.6 Two-dimensional outcomes

Supplementary file S6 shows the cluster rank plots of the effective rate and adverse events (liver injury, leukopenia, and gastrointestinal reactions). Based on SUCRA values, two nodes representing TG1.0+TCM and TG1.5+TCM were distributed around the 45-degree diagonal line (Supplementary Fig. S6.1, Fig. S6.2), demonstrating that these interventions balanced efficacy and safety. Furthermore, because the node representing TG1.5+TCM was the farthest from the origin of the coordinates, TG1.5+TCM had the best intervention effect, followed by TG1.0+TCM. Supplementary Fig. S6.3 shows that TG1.0+TCM and TG1.5+TCM were away from the 45-degree diagonal line. Their distances from their origins were similar.

#### 3.5.7 Publication bias

**Fig. 6:**
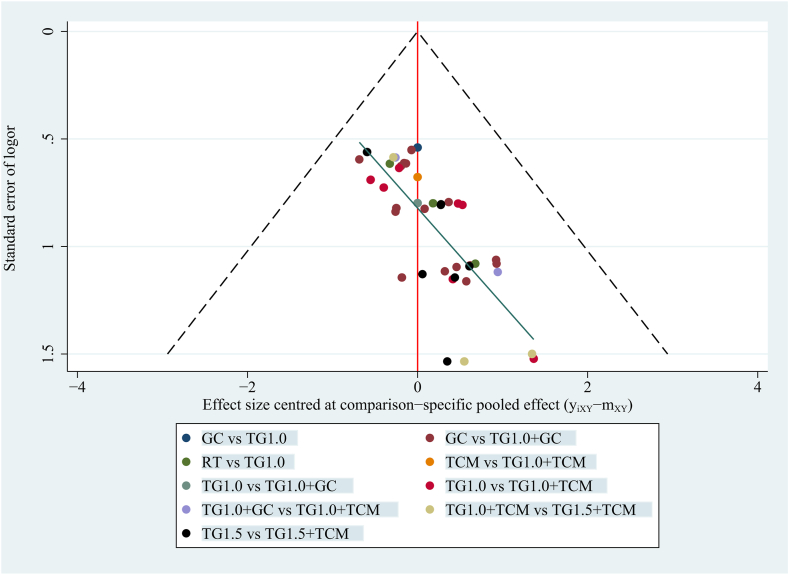
The funnel plot of effective rate. Abbreviations: **TG1.0,** Tripterygium Glycosides (dose: 1 mg/kg/day); **TG1.5,** Tripterygium Glycosides (dose: 1.5 mg/kg/day); **TCM,** traditional Chinese medicine; **GC,** glucocorticoid; **RT,** routine treatment.

The number of RCTs reporting the effective rate, recurrence rate, liver injury, and gastrointestinal events was greater than 10. The publication bias of these four outcomes was evaluated using funnel plots. Legends with different colours represent comparisons between different interventions. Fig. 6 and Supplementary Fig. S5.2.2, Fig. S5.3.4, and Fig. S5.5.5 show that the funnel plots of the abovementioned outcomes were visually symmetrical, indicating no publication bias. However, many studies are scattered at the bottom, mainly because of small sample sizes.

## 4 Discussion

Our NMA assessed the relative efficacy and safety of the different interventions in patients with IgAVN. Compared to previous meta-analyses, our NMA considered the dose and provided more information. This study aimed to evaluate different doses of TG and their combinations to determine the best treatment for IgAVN. All 42 RCTs involving 3173 participants provided evidence that TG1.5, TG1.0+GC, TG1.0+TCM, and TG1.5+TCM were associated with a significant improvement in efficacy compared with TG1.0. The evidence provided by 11 RCTs enrolling 821 patients with IgAVN supports the benefits of TG1.0+TCM and TG1.0+GC in reducing recurrence. TG1.0+TCM and TG1.5+TCM showed apparent advantages in terms of the incidence of adverse events, especially liver injury and leukopenia. From this study, it can be concluded that the therapeutic effect of TG on IgAVN is dose-dependent. The higher the dose, the better the efficacy. With increasing dose, there was no significant difference in the incidence of adverse events (P > 0.05). We also found that a combination of Chinese herbal medicines reduced the adverse effects of TG.

The primary outcome was the effectiveness rate. Urinary proteins and erythrocytes are two critical observational indicators for evaluating the therapeutic effects. Any change in 24-h urine protein levels was assumed to directly reflect a therapeutic effect. Although urinary erythrocytes recede more slowly than proteins, they remain an essential criterion for judgment [12]. NMA results showed that TG1.5+TCM was the best treatment in terms of efficacy. Moreover, it was significantly better than TG 1.0+TCM in terms of efficacy. The NMA results were generally consistent with those of the PMA. Compared to TCM alone, the combination of TCM and TG showed more significant advantages. IgAVN is an immune-related disease [1]. IgAVN treatment focuses on controlling immune inflammation. Studies have shown that TG is anti-inflammatory, inhibits the immune response, repairs and protects kidney podocytes, and improves glomerular capillary permeability [13]. Compared to TG1.0, TG1.5 was more effective. No previous meta-analysis has been performed in terms of dose. Our results showed a positive correlation between the dose and effective rate. However, further clinical studies are required to confirm this hypothesis.

Another essential observational indicator is the recurrence rate. There may be no association between the initial clinical presentation of IgAVN and the final renal outcome. Severe haematuria and proteinuria can also lead to spontaneous remission, and patients with mild symptoms may eventually develop end-stage kidney disease [14]. Therefore, follow-up is essential to monitor disease recurrence. The NMA results showed that TG1.0+TCM was the best treatment for recurrence. Compared with GC, TG1.0+GC was better at reducing the recurrence rate, which is consistent with the results of previous meta-analyses studies' results [15]. The recommended treatment for IgAVN is GC combined with antihypertensives, anticoagulants, and other comprehensive treatments. However, GC can relieve some clinical symptoms quickly but cannot prevent recurrence [16]. These results indicate that GC with TG could effectively reduce the recurrence rate. In this study, few RCTs provided information about the recurrence rate, so TG 1.0 and TG 1.5 could not be compared. The follow-up time of the included RCTs was short, mostly approximately one year. A complete comparison can be obtained if there are additional relevant studies.

This study used liver injury, leukopenia, and gastrointestinal events as leading indicators of adverse events. Drug-induced liver injury is a common adverse clinical outcome. It usually manifests as elevated levels of alanine aminotransferase, aspartate aminotransferase, and alkaline phosphatase (ALP). Previous studies have reported that large doses of TG (60–90 mg per day) can cause liver damage within a short time (6–10 days) [17]. The chemical constituents of TG include triptolide (TP) and celastrol (Cel). They are primary active ingredients with immunosuppressive, anti-inflammatory, anti-obesity, and anti-cancer effects [18]. However, many studies have reported that TP can damage the liver and haematological and reproductive systems [19,20]. The NMA results showed that TG1.0+TCM was the best treatment, with the lowest incidence of liver injury. However, in PMA, TG1.0+TCM did not show a significant effect compared to TG1.0 (P > 0.05). The reason for this difference may be that only one RCT compared TG1.0 with TG1.0+TCM. We still take the NMA results as the final conclusion, as they have direct and indirect comparison effects. A previous NMA study [21] of six kinds of Chinese patent medicines treating children with IgAVN showed that TCM could reduce the incidence of adverse events compared to conventional treatment. Many studies, including clinical trials, have shown that TCM has unique advantages for treating liver injury [22]. This study showed no significant differences in TG1.0, TG1.0+GC, and GC, similar to the previous meta-analysis findings [23]. Currently, no RCTs and meta-analyses compare TG 1.0 with TG 1.5 in liver injury events. This NMA result showed no significant difference between TG 1.0 and TG 1.5 in liver dysfunction. However, considering the low quality of the enrolled RCTs and the small sample size, its accuracy needs to be further clarified by expanding the sample size.

In China, many clinicians have found that patients could experience haematological changes. Some researchers have examined the incidence of adverse haematological events. The results showed that the three major haematological adverse events were leukopenia (OR 5.6 %, CI 4.3 %–7.3 %), haemoglobin reduction (OR 1.7 %, CI 0.5 %–5.0 %), and thrombocytopenia (OR 1.8 %, CI 1.0 %–3.1 %) [24]. Leukopenia had the highest incidence; therefore, we considered it a major adverse event observed in this study. The NMA results showed that TG1.0+TCM was the best treatment, with the lowest incidence of leukopenia. However, only a few RCTs provided information about leukopenia events, which lacked a comparison between GC and TG 1.0.

The gastrointestinal reactions are also a leading indicator of adverse events. Its main clinical manifestations are nausea, vomiting, abdominal distension, abdominal pain, and diarrhoea. A retrospective analysis of adverse events showed that the incidence of gastrointestinal events was 4.3 % and mainly occurred within 1–3 days after medication [25]. The NMA results showed no statistically significant differences in the incidence of gastrointestinal events among the different interventions. Although drugs can cause gastrointestinal reactions, most are mild. These nonspecific changes can spontaneously regress after treatment withdrawal. Animal studies have also shown that TG can cause gastrointestinal reactions in rats; however, it is tolerable and gradually regresses after long-term administration. A positive correlation was also found between toxicity and dose [26]. However, this study did not find that the gastrointestinal events were related to the dose. Compared with animal experiments, the dose gap in this study was not sufficiently large, which may be the reason for the lack of a significant difference. In addition, according to the results of our subgroup analysis, TCM showed significant benefits in the child group.

In the subgroup analysis, the results for adults and children were almost consistent with the overall situation, indirectly demonstrating that the overall results were reliable and stable. At present, there is no significant difference between the treatment of IgAVN in adults and children, and adult treatment options are often drawn from paediatric guidelines. It is feasible to combine the statistical analyses of patients of different ages with IgAVN.

To further explore the best drugs that can balance efficacy and safety, we conducted a comprehensive evaluation using cluster rank plots. The first group comprised liver injury events and efficacy, whereas the second group comprised gastrointestinal events and efficacy. TG1.5+TCM was the furthest from the origin of the coordinates in the 2 cluster rank plots (Supplementary Fig. S6.1, Fig. S6.2), indicating that it had positive effects on different outcomes. The third group comprised patients with leukopenia events and efficacy (Supplementary Fig. S6.3). Only a few RCTs provided information on leukopenia events with the three interventions involved. Therefore, the dots in the figure are scattered, and the distances from the origin of the coordinates (TG1.0+TCM and TG1.5+TCM) are similar. According to the NMA results, TG1.5+TCM was significantly superior to TG1.0+TCM in terms of the efficacy rate. There was no significant difference between TG1.5 + TCM and TG1.0 + TCM in the leukopenia events. Overall, a higher dose of TG significantly increased the effective rate but did not increase the adverse effects. Therefore, TG1.5+TCM may be the best treatment option for IgAVN.

The strengths of this study are as follows: previous meta-analyses focused only on comparing TG with other therapeutic interventions for the treatment of IgAVN. To the best of our knowledge, this study is the first to examine the correlations among the dosage, safety, and effectiveness of TG. It is also the first study to investigate the combination of TCM with varying doses of TG. This study has several methodological advantages. The integration of direct and indirect evidence has contributed to a more comprehensive understanding of the therapeutic landscape for IgAVNs. We performed analysis of SUCRA values to further rank several therapeutic interventions and created cluster rank plots to provide a multidimensional evaluation from the perspectives of efficacy and safety, while also improving the readability of the data. Additionally, the subgroup analyses based on age enhanced the robustness of our findings.

However, the NMA has some limitations. First, multicentre, large-sample studies are lacking. The included RCTs lacked adequate information on double blinding and allocation and did not have consistent diagnostic and clinical response rate criteria. However, high-quality studies are required to confirm this hypothesis. Second, after literature screening and selection, a substantial number of RCTs were excluded. This exclusion predominantly affected the investigation of certain treatment regimens, notably TG1.5+GC, limiting the dose stratification of TG + GC. The enrolled RCTs did not consider patients with severe IgAVN as observation subjects. Therefore, the efficacy and safety of immunosuppressants and plasmapheresis could not be discussed. Third, all the RCTs were conducted in China. Of these, only a few RCTs have focused on adults with IgAVN. This demographic specificity limits the generalisability of our findings.

Therefore, more high-quality, large-scale RCTs should be conducted to validate these results. Future studies should focus on patients with severe IgAVN. Expanding the therapeutic modalities explored in these trials, such as immunosuppressants and TG1.5+GC, is essential. TG application has been analysed as an effective treatment method for IgAVN; however, the molecular mechanisms driving its therapeutic effects need to be investigated in detail. The integration of TCM with TG appears to enhance efficacy while mitigating toxicity, and the mechanistic basis of this synergistic effect requires in-depth exploration.

## 5 Conclusion

The results showed a positive correlation between the dose and efficacy. TG1.5 was superior to TG1.0 in treating IgAVN, with no significant difference in adverse events. Compared with GC, TG1.0+GC is more effective and can reduce recurrence. Furthermore, TCM can boost the efficacy and reduce adverse events when combined with TG. In conclusion, we consider TG1.5+TCM as the best treatment for IgAVN. This result sheds new light on the clinical application of TG, not only to determine the precise TG dosage, but also to underscore the potential of TCM to improve therapeutic outcomes through its synergistic effects with conventional medicine.

## Funding statement

This work was supported by the Academic Inheritance and Communication Project of the China Academy of Chinese Medical Sciences under Grant CI2022E021XB, and the Jiangsu Province Traditional Chinese Medicine Science and Technology Development Plan Project under Grant ZD202205.

## Data availability statement

Data will be made available on request.

## Ethics statement

Review and approval by an ethics committee was not needed for this study because this work was conducted on the basis of RCTs that have already received ethical approval.

**Table:** List of abbreviations

CI	Confidence interval
GC	Glucocorticoids
IgAV	IgA vasculitis
IgAVN	IgA vasculitis nephritis
NMA	Network meta-analysis
OR	Odds ratio
PMA	Pairwise meta-analysis
PRISMA	Preferred Reporting Items for Systematic Review and Meta-Analysis
PROSPERO	International Prospective Register of Systematic Reviews
RCT	Randomized controlled trial
RT	Routine treatment
SUCRA	Surface Under the Cumulative Ranking Curve
TCM	Traditional Chinese medicine
TG	Tripterygium glycosides
TG1.0	Tripterygium glycosides (dose: 1 mg/kg/day)
TG1.5	Tripterygium glycosides (dose: 1.5 mg/kg/day)

## CRediT authorship contribution statement

**Hui Sun:** Writing – original draft, Visualization, Software, Methodology, Formal analysis, Conceptualization. **Lijia Liu:** Writing – original draft, Visualization, Methodology, Investigation, Formal analysis, Data curation. **Gang Wang:** Writing – review & editing, Project administration, Funding acquisition, Formal analysis. **Wei Kong:** Writing – review & editing, Formal analysis. **Yu Zhong:** Writing – review & editing, Funding acquisition. **Lan Yi:** Resources. **Yanqin Zou:** Supervision, Funding acquisition.

## Declaration of competing interest

The authors declare the following financial interests/personal relationships which may be considered as potential competing interests:Yanqin Zou reports financial support was provided by 10.13039/501100005892China Academy of Chinese Medical Sciences. Yu Zhong reports financial support was provided by Jiangsu Province Administration of Traditional Chinese Medicine. If there are other authors, they declare that they have no known competing financial interests or personal relationships that could have appeared to influence the work reported in this paper.
