# Supplementary material for: Comparison of different doses of Tripterygium glycosides treating in IgA vasculitis nephritis: A Bayesian network meta-analysis
Source: Heliyon. 2024 Jul 14;10(14):e34329. doi: 10.1016/j.heliyon.2024.e34329 (PMC11305250; doi:10.1016/j.heliyon.2024.e34329)

Supplementary Sections

[S1. PRISMA checklist for network meta-analysis 3](#_Toc155027437)

[S2. Search strategy 8](#_Toc155027438)

[S3. Characteristics of RCTs involved in the study 10](#_Toc155027439)

[Table S3.1. Characteristics of included RCTs 10](#_Toc155027440)

[S3.2. References of included RCTs 14](#_Toc155027441)

[S4. Risk of bias assessment of included RCT 18](#_Toc155027442)

[Fig. S4.1 Risk of bias graph 18](#_Toc155027443)

[Fig. S4.2Risk of bias summary. 19](#_Toc155027444)

[S5. Supplementary information for all the outcomes of interest 20](#_Toc155027445)

[S5.1 Effective rate 21](#_Toc155027446)

[Fig. S5.1.1 Node-splitting analysis of inconsistency 21](#_Toc155027447)

[Fig. S5.1.2 Results of heterogeneity analysis 22](#_Toc155027448)

[S5.1.3 NMA including trials with participants' age <18. 24](#_Toc155027449)

[S5.1.4 PMA including trials with participants' age >18. 29](#_Toc155027450)

[S5.2 Recurrence rate 31](#_Toc155027451)

[Fig. S5.2.1 Results of heterogeneity analysis 31](#_Toc155027452)

[Fig. S5.2.2 The funnel plot 32](#_Toc155027453)

[S5.2.3 PMA including trials with participants' age >18. 33](#_Toc155027454)

[S5.3 Liver injury events 35](#_Toc155027455)

[Fig. S5.3.1 Results of heterogeneity analysis 35](#_Toc155027456)

[Table S5.3.2 The league table of liver injury events 36](#_Toc155027457)

[Fig. S5.3.3 The forest plot 37](#_Toc155027458)

[Fig. S5.3.4 The funnel plot 38](#_Toc155027459)

[S5.3.5 NMA including trials with participants' age <18. 39](#_Toc155027460)

[S5.4 Leukopenia events 43](#_Toc155027461)

[Fig. S5.4.1 Results of heterogeneity analysis 43](#_Toc155027462)

[Table S5.4.2 The league table of leukopenia events 44](#_Toc155027463)

[Fig. S5.4.3 The forest plot 45](#_Toc155027464)

[S5.5 Gastrointestinal events 46](#_Toc155027465)

[Fig. S5.5.1 Node-splitting analysis of inconsistency 46](#_Toc155027466)

[Fig. S5.5.2 Results of heterogeneity analysis 47](#_Toc155027467)

[Table S5.5.3 The league table of gastrointestinal events 48](#_Toc155027468)

[Fig. S5.5.4 The forest plot 49](#_Toc155027469)

[Fig. S5.5.5 The funnel plot 50](#_Toc155027470)

[S5.5.6 NMA including trials with participants' age <18. 51](#_Toc155027471)

[S6. The clusterank plots 55](#_Toc155027472)

[Fig. S6.1 Effective rate and liver injury events 55](#_Toc155027473)

[Fig. S6.2 Effective rate and leukopenia events 56](#_Toc155027474)

[Fig. S6.3 Effective rate and gastrointestinal events 57](#_Toc155027475)

S1. PRISMA checklist for network meta-analysis

| **Section/topic** | **#** | **Checklist item** | **Reported on page #** |
| --- | --- | --- | --- |
| **TITLE** | | |  |
| Title | 1 | Identify the report as a systematic review incorporating a network meta-analysis (or related form of  meta-analysis). | 1 |
| **ABSTRACT** | | |  |
| Structured summary | 2 | Provide a structured summary including, as applicable:  Background: main objectives  Methods: data sources; study eligibility criteria, participants, and interventions; study appraisal; and synthesis methods, such as network meta-analysis.  Results: number of studies and participants identified; summary estimates with corresponding confidence/credible intervals; treatment rankings may also be discussed. Authors may choose to summarize pairwise comparisons against a chosen treatment included in their analyses for brevity.  Discussion/Conclusions: limitations; conclusions and implications of findings.  Other: primary source of funding; systematic review registration number with registry name. | 2 |
| **INTRODUCTION** | | |  |
| Rationale | 3 | Describe the rationale for the review in the context of what is already known, including mention of why a network meta-analysis has been conducted | 3-4 |
| Objectives | 4 | Provide an explicit statement of questions being addressed with reference to participants, interventions, comparisons, outcomes, and study design (PICOS). | 3-4 |
| **METHODS** | | |  |
| Protocol and registration | 5 | Indicate if a review protocol exists and if and where it can be accessed (e.g., Web address), and, if available, provide registration information including registration number. | 4 |
| Eligibility criteria | 6 | Specify study characteristics (e.g., PICOS, length of follow-up) and report characteristics (e.g., years considered, language, publication status) used as criteria for eligibility, giving rationale. Clearly describe eligible treatments included in the treatment network, and note whether any have been clustered or merged into the same node (with justification). | 4-5 |
| Information sources | 7 | Describe all information sources (e.g., databases with dates of coverage, contact with study authors to identify additional studies) in the search and date last searched. | 5 |
| Search | 8 | Present full electronic search strategy for at least one database, including any limits used, such that it could be repeated. | 5 |
| Study selection | 9 | State the process for selecting studies (i.e., screening, eligibility, included in systematic review, and, if applicable, included in the meta-analysis). | 5 |
| Data collection process | 10 | Describe method of data extraction from reports (e.g., piloted forms, independently, in duplicate) and any processes for obtaining and confirming data from investigators. | 5 |
| Data items | 11 | List and define all variables for which data were sought (e.g., PICOS, funding sources) and any assumptions and simplifications made. | 5 |
| Geometry of the network | 12 | Describe methods used to explore the geometry of the treatment network under study and potential biases related to it. This should include how the evidence base has been graphically summarized for presentation, and what characteristics were compiled and used to describe the evidence base to readers | 6 |
| Risk of bias within individual studies | 13 | Describe methods used for assessing risk of bias of individual studies (including specification of whether this was done at the study or outcome level), and how this information is to be used in any data synthesis. | 6 |
| Summary measures | 14 | State the principal summary measures (e.g., risk ratio, difference in means). Also describe the use of additional summary measures assessed, such as treatment rankings and surface under the cumulative ranking curve (SUCRA)  values, as well as modified approaches used to present summary findings from meta-analyses. | 6 |
| Planned methods of analysis | 15 | Describe the methods of handling data and combining results of studies for each network meta-analysis. This should include, but not be limited to: Handling of multigroup trials; Selection of variance structure; Selection of prior distributions in Bayesian analyses; and Assessment of model fit. | 6 |
| Assessment of inconsistency | 16 | Describe the statistical methods used to evaluate the agreement of direct and indirect evidence in the treatment network(s) studied. Describe efforts taken to address its presence when found. | 6 |
| Risk of bias across studies | 17 | Specify any assessment of risk of bias that may affect the cumulative evidence (e.g., publication bias, selective reporting within studies) | 6 |
| Additional analyses | 18 | Describe methods of additional analyses if done, indicating which were prespecified. This may include, but not be limited to, the following: Sensitivity or subgroup analyses; Meta-regression analyses; Alternative formulations of the treatment network; and Use of alternative prior distributions for Bayesian analyses (if applicable). | 6 |
| **RESULTS** | | |  |
| Study selection | 19 | Give numbers of studies screened, assessed for eligibility, and included in the review, with reasons for exclusions at each stage, ideally with a flow diagram. | 7 |
| Presentation of network structure | 20 | Provide a network graph of the included studies to enable visualization of the geometry of the treatment network. | 8 |
| Summary of network geometry | 21 | Provide a brief overview of characteristics of the treatment network. This may include commentary on the abundance of trials and randomized patients for the different interventions and pairwise comparisons in the network, gaps of evidence in the treatment network, and potential biases reflected by the network structure. | 8 |
| Study characteristics | 22 | For each study, present characteristics for which data were extracted (e.g., study size, PICOS, follow-up period) and provide the citations. | 8-10 |
| Risk of bias within studies | 23 | Present data on risk of bias of each study and, if available, any outcome level assessment. | 10-11 |
| Results of individual studies | 24 | For all outcomes considered (benefits or harms), present, for each study: 1) simple summary data for each intervention group, and 2) effect estimates and confidence intervals. Modified approaches may be needed to deal with information from larger networks. | 12-16 |
| Synthesis of results | 25 | Present results of each meta-analysis done, including confidence/credible intervals. In larger networks, authors may focus on comparisons versus a particular comparator (e.g., placebo or standard care), with full findings presented in an appendix. League tables and forest plots may be considered to summarize pairwise comparisons. If additional summary measures were explored (such as treatment rankings), these should also be presented. | 12-16 |
| Exploration for inconsistency | 26 | Describe results from investigations of inconsistency. This may include such information as measures of model fit to compare consistency and inconsistency models, P values from statistical tests, or summary of inconsistency estimates from different parts of the treatment network. | 16-17 |
| Risk of bias across studies | 27 | Present results of any assessment of risk of bias across studies for the evidence base being studied. | 16-17 |
| Results of additional analyses | 28 | Give results of additional analyses, if done (e.g., sensitivity or subgroup analyses, meta-regression  analyses, alternative network geometries studied, alternative choice of prior distributions for  Bayesian analyses, and so forth). | 11,17-18 |
| **DISCUSSION** | | |  |
| Summary of evidence | 29 | Summarize the main findings, including the strength of evidence for each main outcome; consider their relevance to key groups (e.g., health care providers, researchers, and policymakers). | 19-20 |
| Limitations | 30 | Discuss limitations at study and outcome level (e.g., risk of bias), and at review level (e.g., incomplete retrieval of identified research, reporting bias). Comment on the validity of the assumptions, such as transitivity and consistency. Comment on any concerns regarding network geometry (e.g., avoidance of certain comparisons). | 20 |
| Conclusions | 31 | Provide a general interpretation of the results in the context of other evidence, and implications for future research. | 20 |
| **FUNDING** | | |  |
| Funding | 32 | Describe sources of funding for the systematic review and other support (e.g., supply of data); role of funders for the systematic review. This should also include information regarding whether funding has been received from manufacturers of treatments in the network and/or whether some of the authors are content experts with professional conflicts of interest that could affect use of treatments in the network. | 21 |

S2. Search strategy

**PubMed:**

#1 (((((((((((("IgA Vasculitis"[Mesh]) OR (Henoch-Schonlein purpura[Title/Abstract]))

OR (Henoch purpura[Title/Abstract])) OR (Schonlein disease[Title/Abstract])) OR

(Anaphylactoid Purpura[Title/Abstract])) OR (Allergic Purpura[Title/Abstract])) OR

(Hemorrhagic Vasculitis[Title/Abstract])) OR (Rheumatoid Purpura[Title/Abstract]))

OR (Nonthrombopenic Purpura[Title/Abstract])) OR (purpura

nephritis[Title/Abstract])) OR (purpura nephropathy[Title/Abstract])) OR

(HSPN[Title/Abstract])) OR (HSN[Title/Abstract])

#2 (((((("Tripterygium"[Mesh]) OR (Tripterygium wilfordii)) OR (lei gong teng)) OR

(Leigong Teng)) OR (Thundergod Vine)) OR (Tripterygium hypoglaucum)

#3 #1 AND #2

**Web of Science:**

TS= (IgA Vasculitis OR Henoch-Schönlein Purpura OR Henoch Purpura OR Schonlein disease OR Anaphylactoid Purpura OR Allergic Purpura OR Hemorrhagic Vasculitis OR Rheumatoid Purpura OR Nonthrombopenic Purpura OR purpura nephritis OR purpura nephropathy OR anaphylactic purpura nephritis OR HSPN OR HSN) AND ALL= (Tripterygium OR lei gong teng)

**Cochrane Central Register of Controlled Trials**

IgA Vasculitis OR Henoch-Schönlein Purpura OR Henoch Purpura OR Schonlein disease OR Anaphylactoid Purpura OR Allergic Purpura OR Hemorrhagic Vasculitis OR Rheumatoid Purpura OR Nonthrombopenic Purpura OR purpura nephritis OR purpura nephropathy OR anaphylactic purpura nephritis OR HSPN OR HSN in Title Abstract Keyword AND Tripterygium OR Tripterygium wilfordii OR Tripterygium wilfordii Hook F OR lei gong teng OR Thundergod Vine OR Tripterygium hypoglaucum OR Leigong Teng in All Text

**Embase:**

('iga vasculitis':ti,ab,kw OR 'henoch-schönlein purpura':ti,ab,kw OR 'henoch purpura':ti,ab,kw OR 'schonlein disease':ti,ab,kw OR 'anaphylactoid purpura':ti,ab,kw OR 'allergic purpura':ti,ab,kw OR 'hemorrhagic vasculitis':ti,ab,kw OR 'rheumatoid purpura':ti,ab,kw OR 'nonthrombopenic purpura':ti,ab,kw OR 'purpura nephritis':ti,ab,kw OR 'purpura nephropathy':ti,ab,kw OR 'anaphylactic purpura nephritis':ti,ab,kw OR 'hspn':ti,ab,kw OR 'hsn':ti,ab,kw) AND ('tripterygiums' OR 'tripterygium wilfordii' OR 'tripterygium wilfordius' OR 'wilfordius, tripterygium' OR 'leigong teng' OR 'leigong tengs' OR 'teng, leigong' OR 'tengs, leigong' OR 'thundergod vine' OR 'thundergod vines' OR 'vine, thundergod' OR 'vines, thundergod' OR 'tripterygium hypoglaucum' OR 'tripterygium hypoglaucums' OR 'hypoglaucums, tripterygium')

**China National Knowledge Infrastructure Library (CNKI) :**

#1 SU=过敏性紫癜 OR SU=紫癜性肾炎 OR SU=紫癜肾 OR SU=HSPN OR SU= 紫癜肾炎

#2 SU=雷公藤 OR SU=雷公藤属 OR SU=雷公藤甲素 OR SU=雷公藤乙素 OR

SU=雷公藤红素 OR SU=雷公藤多苷 OR SU=雷公藤多甙

#3 #1 AND #2

S3. Characteristics of RCTs involved in the study

## Table S3.1 Characteristics of included RCTs

| **Study ID** | **SEX (M/F)** | **Age**  **(years)** | **Course (months)** | **Sample size** | **Treatment** | **Control** | **Outcomes** | **Duration of treatment**  **(weeks/days)** |
| --- | --- | --- | --- | --- | --- | --- | --- | --- |
| **CAI Hongkai**,  **2015** | 54/36 | 7.79(7.5 ± 1.9 / 8.1 ± 2.4) | 11.27(11.6 ± 3.3/10.9 ± 2.8) | 90(47/43) | TG1.0+TCM | TG1.0 | effective rate | 12W |
| **CAI Yubin,**  **2012** | 46/34 | 38.81 | 49.8 | 80(40/40) | TG1.0+GC | GC | effective rate, recurrence rate, gastrointestinal events | 12W |
| **CHEN Hao,**  **2012** | 32/20 | 8.4 | 0.3 | 52(26/26) | TG1.0+TCM | TG1.0 | effective rate, liver injury events | 8W |
| **CHEN Min,**  **2015** | 68/52 | 41.6 ± 10.2 | NA | 120(60/60) | TG1.0+GC | GC | effective rate, recurrence rate | 4W |
| **CHEN Tingshun,**  **2016** | NA | ≤18 | NA | 100(50/50) | TG1.0+TCM | TCM | effective rate, gastrointestinal events | 12W |
| **DING Ying,**  **2004** | 29/21 | 10.64 | NA | 50(30/20) | TG1.0+TCM | TG1.0 | effective rate | 12W |
| **DONG Lei,**  **2016** | 45/33 | 6.77 | NA | 78(39/39) | TG1.0+GC | GC | effective rate, gastrointestinal events, leukopenia events | NA |
| **DU Yanbin,**  **2018** | 50/30 | 40.9 | 68.98 | 80(40/40) | TG1.0+GC | GC | effective rate, recurrence rate | 8W |
| **HE Chuanmei,**  **2020** | 60/36 | 8.3 | 3.56 | 96(48/48) | TG1.0 | RT | effective rate, gastrointestinal events, liver injury events, leukopenia events | 5D |
| **JIE Dongying,**  **2011** | 47/33 | 7.2 ± 1.7 | 5.9 ± 1.7 | 80(40/40) | TG1.0+TCM | TG1.5+TCM | effective rate, liver injury events, leukopenia events | 8W |
| **JIN Ruixia,**  **2009** | 29/43 | 18.7 ± 14.2 | NA | 72(36/36) | TG1.0+GC | GC | effective rate | 12W |
| **JING Shijuan,**  **2019** | 33/39 | 33.59 | 1.31 | 72(24/24/24) | TG1.0+TCM、TG1.5+TCM | TCM | effective rate, gastrointestinal events | 12W |
| **KU Laijuan,**  **2020** | 53/43 | 8.61 | 0.698 | 96(48/48) | TG1.0+TCM | TG1.0 | effective rate | 12W |
| **LI Gaofeng,**  **2021** | 43/37 | 7.99 | 0.605 | 80(40/40) | TG1.0+TCM | TG1.0 | effective rate, gastrointestinal events | 12W |
| **LI Zhenming,**  **2010** | 29/31 | >18 | NA | 60(30/30) | TG1.0+GC | GC | effective rate, gastrointestinal events, liver injury events, leukopenia events | 12W |
| **LIU Yuanjing,**  **2017** | 57/43 | 7.51 | 2.415 | 100(50/50) | TG1.0 | RT | gastrointestinal events, leukopenia events | 12W |
| **LU Xinting,**  **2018** | 77/43 | 44.405 | 70.62 | 120(60/60) | TG1.0+GC | GC | effective rate, recurrence rate | 4W |
| **LV Zongli,**  **2009** | 37/25 | 18.45 | NA | 62(32/30) | TG1.0+TCM | TG1.0+GC | effective rate, recurrence rate, gastrointestinal events, liver injury events | 8W |
| **MA Zhizhong,**  **2017** | 22/24 | 9.6 ± 1.5 | 0.5 | 46(23/23) | TG1.0+TCM | TG1.0+GC | effective rate, recurrence rate | 12W |
| **MENG Qingjun,**  **2016** | NA | ≤18 | NA | 80(40/40) | TG1.5+TCM | TG1.5 | effective rate | 12W |
| **NIU Dongchun,**  **2019** | 55/37 | 8.575 | 2.455 | 92(46/46) | TG1.0+GC | GC | effective rate, gastrointestinal events | 8W |
| **QIU Jing,**  **2017** | 47/33 | 8.45 | 0.5495 | 80(40/40) | TG1.0+TCM | TG1.0 | effective rate, gastrointestinal events | 12W |
| **SHI Zumei,**  **2017** | 71/37 | 45.5 | 6.3 | 108(54/54) | TG1.0+GC | GC | effective rate | 4W |
| **SONG Chaozheng,**  **2004** | NA | 3~14 | 1.27 | 59(29/30) | TG1.0 | GC | effective rate | 8W |
| **SUN Yinan,**  **2022** | 20/18 | 9.15 ± 1.39 | 66.36 ± 14.76 | 38(19/19) | TG1.0+TCM | TG1.0 | effective rate | 8W |
| **WANG Pengjin,**  **2016** | 53/47 | 40.74 | 48.72 | 100(50/50) | TG1.0+GC | GC | effective rate | NA |
| **WANG Zhexiong,**  **1994** | NA | 7.817 | NA | 63(24/19/20) | TG1.5 | GC、RT | recurrence rate | 24W |
| **WEN Yuling,**  **2014** | 48/39 | 7.101 | 5.801 | 87(44/43) | TG1.5+TCM | TG1.5 | effective rate, liver injury events, leukopenia events | 8W |
| **XIE Xiaoshu,**  **2016** | 31/29 | 8.05 | NA | 60(30/30) | TG1.5+TCM | TG1.5 | effective rate, liver injury events, leukopenia events | 12W |
| **YANG Meng,**  **2019** | 73/51 | 6.45 | 1.045 | 124(62/62) | TG1.5+TCM | TG1.5 | effective rate, leukopenia events | 12W |
| **YANG Yang,**  **2017** | 17/13 | 36.25 | NA | 30(15/15) | TG1.0+GC | GC | effective rate, recurrence rate | 4W |
| **YIN Hongwei,**  **2016** | 35/39 | 6.2 ± 1.1 | 0.197 ± 0.047 | 74(37/37) | TG1.0+GC | GC | effective rate, gastrointestinal events, liver injury events | 12W |
| **YU Changhong,**  **2020** | 21/15 | 6~14 | NA | 36(18/18) | TG1.5+TCM | TG1.5 | effective rate | 12W |
| **YUE Miao,**  **2006** | 32/20 | 19.26 | NA | 52(32/20) | TG1.0+GC | GC | effective rate, recurrence rate, gastrointestinal events, liver injury events, leukopenia events | 6W |
| **ZHAI Wensheng,**  **2018** | 39/31 | 7.45 | 0.1~3 | 70(35/35) | TG1.5+TCM | TG1.5 | effective rate, liver injury events | 12W |
| **ZHANG Guowei,**  **2016** | 51/47 | 9.67 | NA | 98(49/49) | TG1.0 | RT | effective rate | 2W |
| **ZHANG Jian,**  **2013** | 31/29 | 8.05 | NA | 60(30/30) | TG1.5+TCM | TG1.5 | effective rate, liver injury events, leukopenia events | 12W |
| **ZHANG Liang,**  **2017** | 39/31 | 7 | 0.155 | 70(35/35) | TG1.0+GC | GC | effective rate | 8W |
| **ZHANG Sishi,**  **2015** | 50/30 | 9.1 | 2.55 | 80(40/40) | TG1.0+GC | GC | effective rate | 12W |
| **ZHANG Yanyan,**  **2020** | 19/11 | 8.55 | 0.56 | 30(15/15) | TG1.0+GC | GC | effective rate | 12W |
| **ZHEN Guizhen,**  **2007** | 57/33 | 10.47 | NA | 90(30/30.30) | TG1.0+TCM | TG1.5+TCM | effective rate, gastrointestinal events, liver injury events, leukopenia events | 12W |
| **ZHENG Yan,**  **2018** | 45/39 | 7.3 | NA | 84(42/42) | TG1.0 | RT | effective rate | 2W |
| **ZHONG Ruiqiong,**  **2012** | 35/41 | 46.55 | 45 | 76(38/38) | TG1.0+GC | GC | effective rate, recurrence rate | 4W |
| **ZHU Kai,**  **2016** | 76/49 | 42.85 | 53.4 | 127(64/63) | TG1.0+GC | GC | effective rate, recurrence rate | 4W |

Abbreviations: **M/F,** male/female; **NA,** not available; **TG1.0,** Tripterygium Glycosides (dose:1mg/Kg/day); **TG1.5,** Tripterygium Glycosides (dose:1.5mg/Kg/day); **TCM,**

Traditional Chinese Medicine prescription or injection; **GC,** Glucocorticoids; **RT,** Routine treatment; **W,** weeks; **D,** days.

## S3.2 References of included RCTs

1. Cai, H., 2015. Observation of the efficacy of tripterygium glycosides combined with traditional Chinese medicine in the treatment of pediatric Henoch-Schönlein nephritis (blood heat injury type). Medical Information. Jan. 2015. Vol. 28 (1). <https://doi.org/10.3969/j.issn.1006-1959.2015.01.164>

2. Cai, Y., et al., 2012. Effect of tripterygium glycosides combined with prednisone in the treatment of adult purpuric nephritis. Chinese Community Doctors. 14(26), 99-100. <https://doi.org/10.3969/j.issn.1007-614x.2012.26.095>

3. Chen, H., 2012. 26 cases of pediatric Henoch-Schönlein nephritis treated with combination of Chinese and western medcine. Zhejiang Journal of Traditional Chinese Medicine. 47(07), 512. <https://doi.org/10.3969/j.issn.0411-8421.2012.07.029>

4. Chen, M., 2015. Clinical efficacy of tripterygium glycosides combined with prednisone in the treatment of adult purpura nephritis. Asia-Pacific Traditional Medicine. 11(17), 120-121. URL: <https://kns.cnki.net/kcms/detail/42.1727.R.20150907.1420.124.html>

5. Chen, T., Li, L., 2016. Efficacy analysis of tripterygium glycosides combined with Xiangdan injection in pediatric Henoch-Schönlein nephritis. Health Guide. (25), 329-329. URL: <https://d.wanfangdata.com.cn/periodical/ChlQZXJpb2RpY2FsQ0hJTmV3UzIwMjIxMjA1EhF5c2Jqem4teDIwMTYyNTMyNBoIdnU0bW93cnA%3D>

6. Ding, Y., et al., 2004. Clinical observation of “Xueniaoting Granule” and Leigongteng Duodaipian in treating purpuric nephritis. Shanghai Jounal of Traditional Chinese Medicine. (08), 37-38. <https://doi.org/10.16305/j.1007-1334.2004.08.019>

7. Dong, L., 2016. Efficacy and safety of tripterygium glycosides combined with glucocorticoids in the treatment of children purpura nephritis. The World Clinical Medicine. 10(9), 166. URL: <https://d.wanfangdata.com.cn/periodical/ChlQZXJpb2RpY2FsQ0hJTmV3UzIwMjIxMjA1Eg9zamxjeXgyMDE2MDkxMzgaCHZ1NG1vd3Jw>

8. Du, YB., Du, YL., 2018. Effect of adult purpura application tripterygium glycosides and prednisone. Journal of Frontiers of Medicine. 8(4). https://doi.org/[10.3969/j.issn.2095-1752.2018.04.019](http://dx.chinadoi.cn/10.3969/j.issn.2095-1752.2018.04.019)

9. He, C., et al., 2020. Effects and safety analysis of tripterygium glycosides on children with purpura nephritis. World Chinese Medicine. 15(20), 3094-3096. https://doi.org/[10.3969/j.issn.1673-7202.2020.20.019](http://dx.chinadoi.cn/10.3969/j.issn.1673-7202.2020.20.019)

10. Jie, D., et al., 2011. Comparison of efficacy and safety of different doses of TwHF polyglycoside tablets combined with traditional Chinese medicine in the treatment of pediatric purpuric nephritis. Medical Information. 24(06), 2261-2262. https://doi.org/[10.3969/j.issn.1006-1959.2011.06.023](http://dx.chinadoi.cn/10.3969/j.issn.1006-1959.2011.06.023).

11. Jin, R., et al., 2009. Combined application of common threewingnut root and hormone in the treatment of Henoch-Schonlein purpura nephritis. Progress of Anatomical Sciences. 15(01), 24-25. <https://doi.org/10.16695/j.cnki.1006-2947.2009.01.021>

12. Jing, S., et al., 2019. Effects of different doses of tripterygium glycoside combined with compound salvia miltiorrhiza on coagulation function and endothelial cell function in patients with Henoch-Schonlein purpura nephritis. Progress in Modern Biomedicine. 19(09), 1692-1696. <https://doi.org/10.13241/j.cnki.pmb.2019.09.019>

13. Ku, L., 2020. Clinical observation on Yangyin Qingyu decoction on children with allergic purpuric nephritis. Guangming Journal of Chinese Medicine. 0(2). https://doi.org/[10.3969/j.issn.1003-8914.2020.02.020](http://dx.chinadoi.cn/10.3969/j.issn.1003-8914.2020.02.020).

14. Li, G., 2021. Clinical analysis of Yangyin Qingyu decoction combined with tripterygium glycosides tablets in the treatment of allergic purpura nephritis. Journal of Practical Traditional Chinese Medicine. 37(01), 3-4. URL: <https://kns.cnki.net/kcms/detail/detail.aspx?FileName=ZYAO202101004&DbName=CJFQ2021>

15. Li, Z., Yang, X., 2010. The efficacy of tripterygium glycosides combined with prednisone in the treatment of 30 cases of adult purpuric nephritis. Shanxi Medical Journal. 39(11), 1542-1543. https://doi.org/[10.3969/j.issn.1000-7377.2010.11.048](http://dx.chinadoi.cn/10.3969/j.issn.1000-7377.2010.11.048).

16. Liu, Y., et al., 2017. Evaluation of efficacy and safety of tripterygium glycosides in treating pediatric purpura nephritis. Women's Health Research. (03), 149-150. URL: <https://kns.cnki.net/kcms/detail/detail.aspx?FileName=ZWVJ201703107&DbName=CJFQ2017>

17. Lu, X., 2018. Effect evaluation of tripterygium glycosides combined with prednisone in treating adult purpura nephritis. China Continuing Medical Education. 10(09), 113-114. https://doi.org/[10.3969/j.issn.1674-9308.2018.09.059](http://dx.chinadoi.cn/10.3969/j.issn.1674-9308.2018.09.059).

18. Lv, Z., 2009. 32 cases of Henoch-Schönlein nephritis treated with "Liangxue Jiedu" decoction. China's Naturopathy. 17(1). [https://doi.org/10.3969/j.issn.1007-5798.2009.01.034.](http://dx.chinadoi.cn/10.3969/j.issn.1007-5798.2009.01.034)

19. Ma, Z., 2017. Clinical efficacy of TCM differentiation combined with tripterygium glycosides tablets in treating Henoch-Schönlein nephritis. The World Clinical Medicine. 11(2), 6. URL: <https://d.wanfangdata.com.cn/periodical/ChlQZXJpb2RpY2FsQ0hJTmV3UzIwMjIxMjA1Eg9zamxjeXgyMDE3MDIwMDUaCHZ1NG1vd3Jw>

20. Meng, Q., Hua, Q., 2016. Tripterygium wilfordii combined with Xiangdan injection on children with allergic purpura nephritis. Journal of Changchun University of Chinese Medicine. 32(05), 1025-1027. <https://doi.org/10.13463/j.cnki.cczyy.2016.05.051>

21. Niu, D., 2019. A clinical study of tripterygium glycosides combined with high-dose methylprednisolone intravenous impulse therapy in 46 children with Henoch-Schönlein purpura nephritis. Journal of North Pharmacy. 16(01), 62-63. https://doi.org/[10.3969/j.issn.1672-8351.2019.01.048](http://dx.chinadoi.cn/10.3969/j.issn.1672-8351.2019.01.048)

22. Qiu, J., 2017. Clinical study of Yangyin Qingyu decoction combined with tripterygium glycosides tablets in treating pediatric Henoch-Schönlein nephritis. Modern Journal of Integrated Traditional Chinese and Western Medicine. 26(23), 2589-2591. https://doi.org/[10.3969/j.issn.1008-8849.2017.23.029](http://dx.chinadoi.cn/10.3969/j.issn.1008-8849.2017.23.029)

23. Shi, Z., 2017. Effect analysis of tripterygium glycosides combined with prednisone in treating adult purpura nephritis. Health Guide. (42), 264. <https://doi.org/10.3969/j.issn.1006-6845.2017.42.251>

24. Song, C., et al., 2004. Efficacy of tripterygium glycosides combined with alginic sodium diester and cimetidine in the treatment of pediatric Henoch-Schönlein nephritis. Journal of Chinese Modern Pediatrics. 1(1). URL: <https://d.wanfangdata.com.cn/periodical/ChlQZXJpb2RpY2FsQ0hJTmV3UzIwMjMwMzIxEg5RSzIwMDQwMjQwNDI3ORoIbjZ5NjU0dGk%3D>

25. Sun, Y., 2022. Discussion on the application of "Liangxue Xiaoban" decoction and tripterygium glycosides in the treatment of purpuric nephritis. Health Horizon. (1), 129-130. URL: <https://d.wanfangdata.com.cn/periodical/ChlQZXJpb2RpY2FsQ0hJTmV3UzIwMjIxMjA1EhpRS0JKQkQyMDIyMjAyMjAxMTkwMDAxNzQyMRoIdnU0bW93cnA%3D>

26. Wang, P., 2016. Clinical efficacy and safety analysis of tripterygium glycosides combined with prednisone in the treatment of adult purpuric nephritis. Cardiovascular Disease Journal of integrated traditional Chinese and Western Medicine. 4(31), 87+90. <https://doi.org/10.16282/j.cnki.cn11-9336/r.2016.31.064>

27. Wang, Z., 1994. Evaluation of the efficacy of corticosteroids and tripterygium glycosides in pediatric purpuric nephritis. Journal of Wenzhou Medical University. (04), 208-210. URL: <https://kns.cnki.net/kcms/detail/detail.aspx?FileName=WZYX404.006&DbName=CJFQ1994>

28. Wen, Y., Jin, F., 2014. Observation of the efficacy of integrated traditional Chinese and western medicine in the treatment of pediatric purpura nephritis. Modern Journal of Integrated Traditional Chinese and Western Medicine. 23(35), 3927-3929. https://doi.org/[10.3969/j.issn.1008-8849.2014.35.018](http://dx.chinadoi.cn/10.3969/j.issn.1008-8849.2014.35.018).

29. Xie, X., Yuan, S., 2016. The efficacy of "Bupi Yiqi Liangxue" decoction in the treatment of pediatric purpuric nephritis and its effect on the body's immune indexes. Shaanxi Journal of Traditional Chinese Medicine. 37(04), 414-415. https://doi.org/[10.3969/j.issn.1000-7369.2016.04.015](http://dx.chinadoi.cn/10.3969/j.issn.1000-7369.2016.04.015).

30. Yang, M., et al., 2019. Hematuria Prescription No. 2 Combined with Tripterygium Glycoside Tablets in the Treatment for Children of Purpura Nephritis ( Pure Hematuria) 62 Cases of Clinical Observation. Chinese Journal of Ethnomedicine and Ethnopharmacy. 28(11), 80-82. URL: <https://d.wanfangdata.com.cn/periodical/ChlQZXJpb2RpY2FsQ0hJTmV3UzIwMjMwMzIxEhN6Z216bWp5eXp6MjAxOTExMDI2GghkbWUxOWVmNw%3D%3D>

31. Yang, Y., Li, D., 2017. Efficacy of tripterygium glycosides and prednisone in adult patients with purpuric nephritis. Mdical Equipment. 30(21), 144-145. https://doi.org/[10.3969/j.issn.1002-2376.2017.21.098](http://dx.chinadoi.cn/10.3969/j.issn.1002-2376.2017.21.098)

32. Yin, H., Wang, K., 2016. Clinical analysis of glucocorticoids combined with tripterygium glycosides in the treatment of purpuric nephritis in children. J Medical Forum. 37(10), 141-142. URL: <https://kns.cnki.net/kcms/detail/11.5479.r.20161018.1222.006.html>

33. Yu, H., 1992. Analysis of efficacy of tripterygium wilfordii and danshen in the treatment of children purpuric nephritis. Chinese Journal of Integrated Traditional and Western Medicine. (06), 343-344+324. [https://doi.org/10.3321/j.issn:1003-5370.1992.06.005.](http://dx.chinadoi.cn/10.3321/j.issn:1003-5370.1992.06.005)

34. Yue, M., 2006. Clinical observation of tripterygium glycosides combined with glucocorticoids in the treatment of purpuric nephritis. Shanxi Med J. (07), 631-632. https://doi.org/[10.3969/j.issn.0253-9926.2006.07.032](http://dx.chinadoi.cn/10.3969/j.issn.0253-9926.2006.07.032).

35. Zhai, W., et al., 2018. Clinical observation on 35 cases of children purpura nephritis with hematuria and proteinuria treated by combination of "purpura Kidney No.1" prescription and tripterygium glycosides. Lishizhen Medicine and Materia Medica Research. 29(01), 131-133. URL: <https://kns.cnki.net/kcms2/article/abstract?v=TOI_Fm_Rdm9rQcDmmzGq04Wfud9-wariYf4DuK-4AX4u9dmu3j3A9xoUhoD3UXofETjrpQ4ZzbiOer-Ccay0eKExZRBXMunryJ2WzWqb5SXKr8RsJDQI650pIqg4_qGlqzwO_kWge3k=&uniplatform=NZKPT&language=CHS>

36. Zhang, G., 2016. Efficacy analysis of tripterygium glycosides in the treatment of pediatric purpura nephritis and its effect on the immune function of patients. World Latest Medicine Information. 16(69), 105+108. URL: <https://kns.cnki.net/kcms2/article/abstract?v=TOI_Fm_Rdm8FgO93ebfs79ywbTqxSGqvkaGfSTXeIutuA1g5jq3-EBTRMKDMrZco-2pcpQ5j7yu5UfuZDOfuehe2wnj4lXnyjc9wuLRZebHQUiC4hhe1oNgStrtwIesL_Wr9CymSLjw=&uniplatform=NZKPT&language=CHS>

37. Zhang, J., et al., 2013. Clinical curative effect on Henoch-Schonlein purpura nephritis ( HSPN) of homemade prescription and correlation research on immune-regulating mechanism. Chinese Journal of Experimental Traditional Medical Formulae. 19(22), 309-312. https://doi.org/[10.11653/syfj2013220309](http://dx.chinadoi.cn/10.11653/syfj2013220309)

38. Zhang, L., 2017. Clinical analysis of glucocorticoids combined with tripterygium glycosides in the treatment of pediatric purpura nephritis. The Journal of Medical Theory and Practice. 30(02), 243-245. <https://doi.org/10.19381/j.issn.1001-7585.2017.02.051>

39. Zhang, S., Zhang, Y., 2015. Clinical observation of tripterygium glycosides, prednisone combined with traditional Chinese medicine in treating children's Henoch-Schonlein purpura nephritis. Journal of Logistics University of PAPF（Medical Sciences）. 24(05), 389-390. <https://doi.org/10.16548/j.2095-3720.2015.05.018>

40. Zhang, Y., 2020. Observation on the efficacy of prednisone combined with tripterygium wilfordii polyglycoside in the treatment of allergic purpura nephritis. Kangyi (20), 271. https://doi.org/ 10.12332/j.issn.2095-6525.2020.20.266

41. Zheng, G., 2007. Efficacy and safety of different dosage multi-glycoside of Tripterygium Wilfordii Hook. F.with Chinese medicine for hematuria in children with Henoch-Schönlein purpura nephritis. HeNan University of Traditional Chinese Medicine. https://doi.org/[10.7666/d.y1095204](http://dx.chinadoi.cn/10.7666/d.y1095204)

42. Zheng, Y., 2018. Analysis of the efficacy and effect of tripterygium glycosides in the treatment of pediatric purpura nephritis. Diet Health. 5(8), 61-62. <https://doi.org/10.3969/j.issn.2095-8439.2018.08.075>

43. Zhong, R., et al., 2012. Clinical observation of tripterygium glycosides combined with prednisone in the treatment of 38 adult purpuric nephritis. Jilin Medical Journal. 33(12), 2570-2571. https://doi.org/[10.3969/j.issn.1004-0412.2012.12.093](http://dx.chinadoi.cn/10.3969/j.issn.1004-0412.2012.12.093)

44. Zhu, K., 2016. Study on the effect of treatment with tripterygium glycosides and prednisone in adult purpuric nephritis. Guide of China Medicine. 14(32), 143-144. <https://doi.org/10.15912/j.cnki.gocm.2016.32.124>

S4. Risk of bias assessment of included RCT

## Fig. S4.1 Risk of bias graph

**
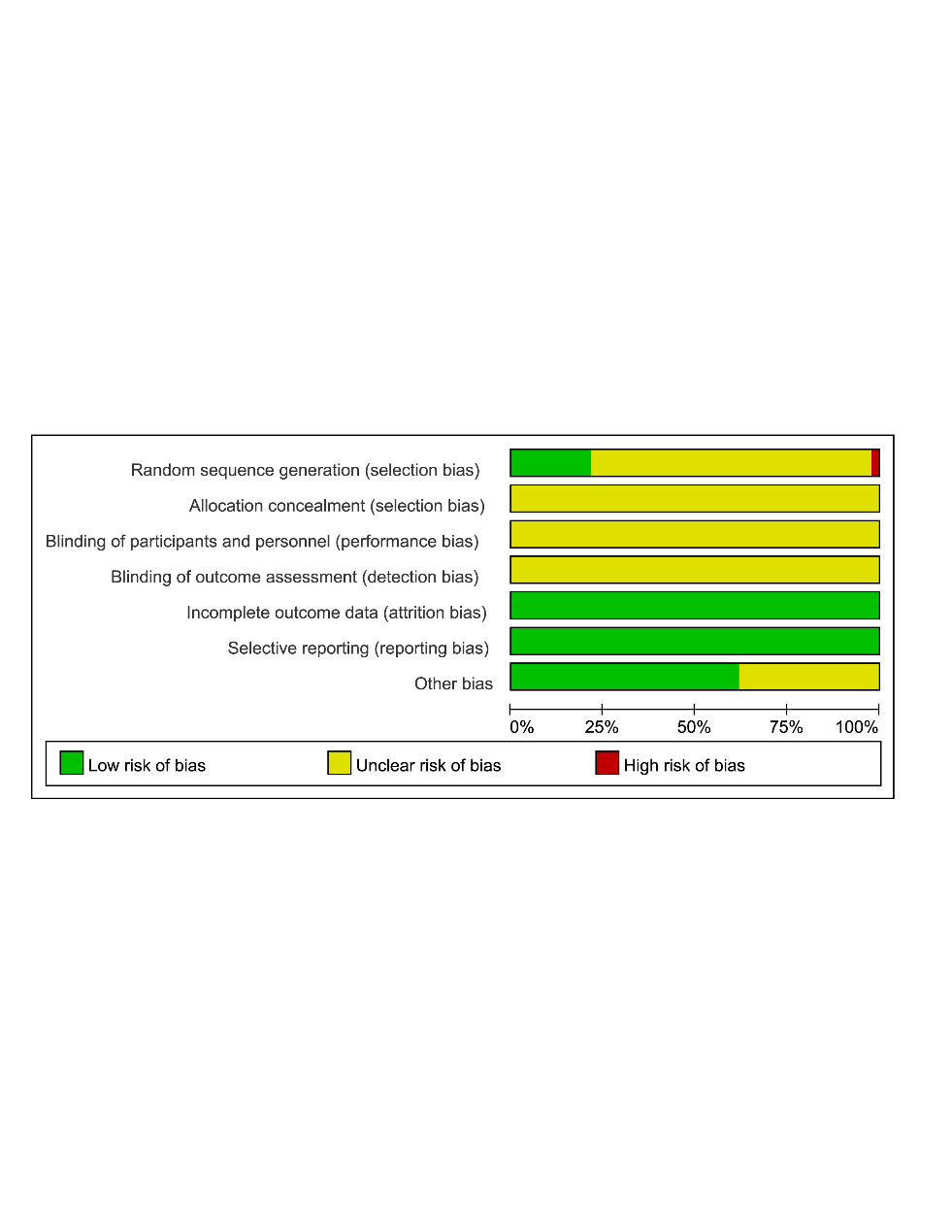
**

## Fig. S4.2 Risk of bias summary.


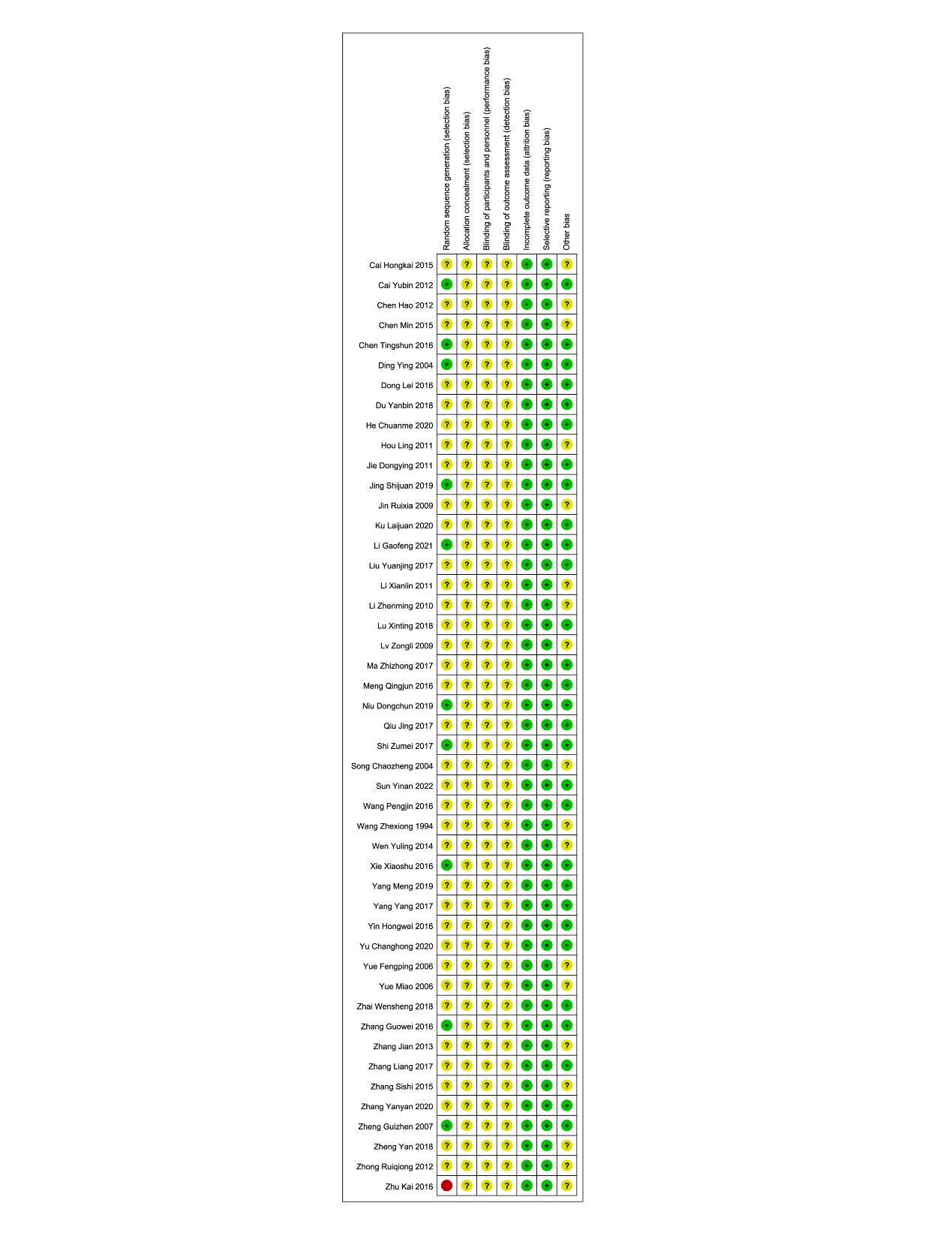


S5. Supplementary information for all the outcomes of interest

## S5.1 Effective rate

### Fig. S5.1.1 Node-splitting analysis of inconsistency


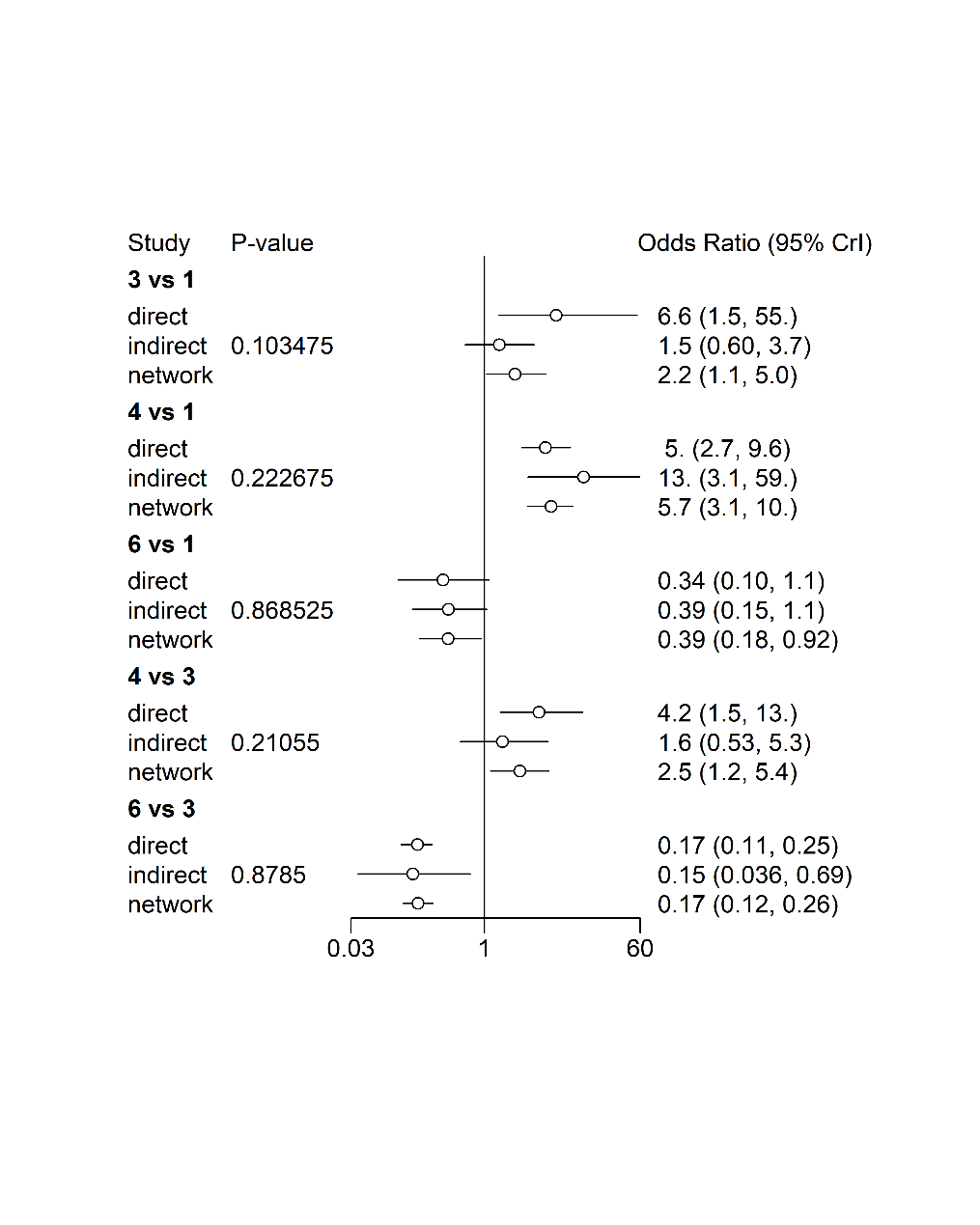


### Fig. S5.1.2 Results of heterogeneity analysis


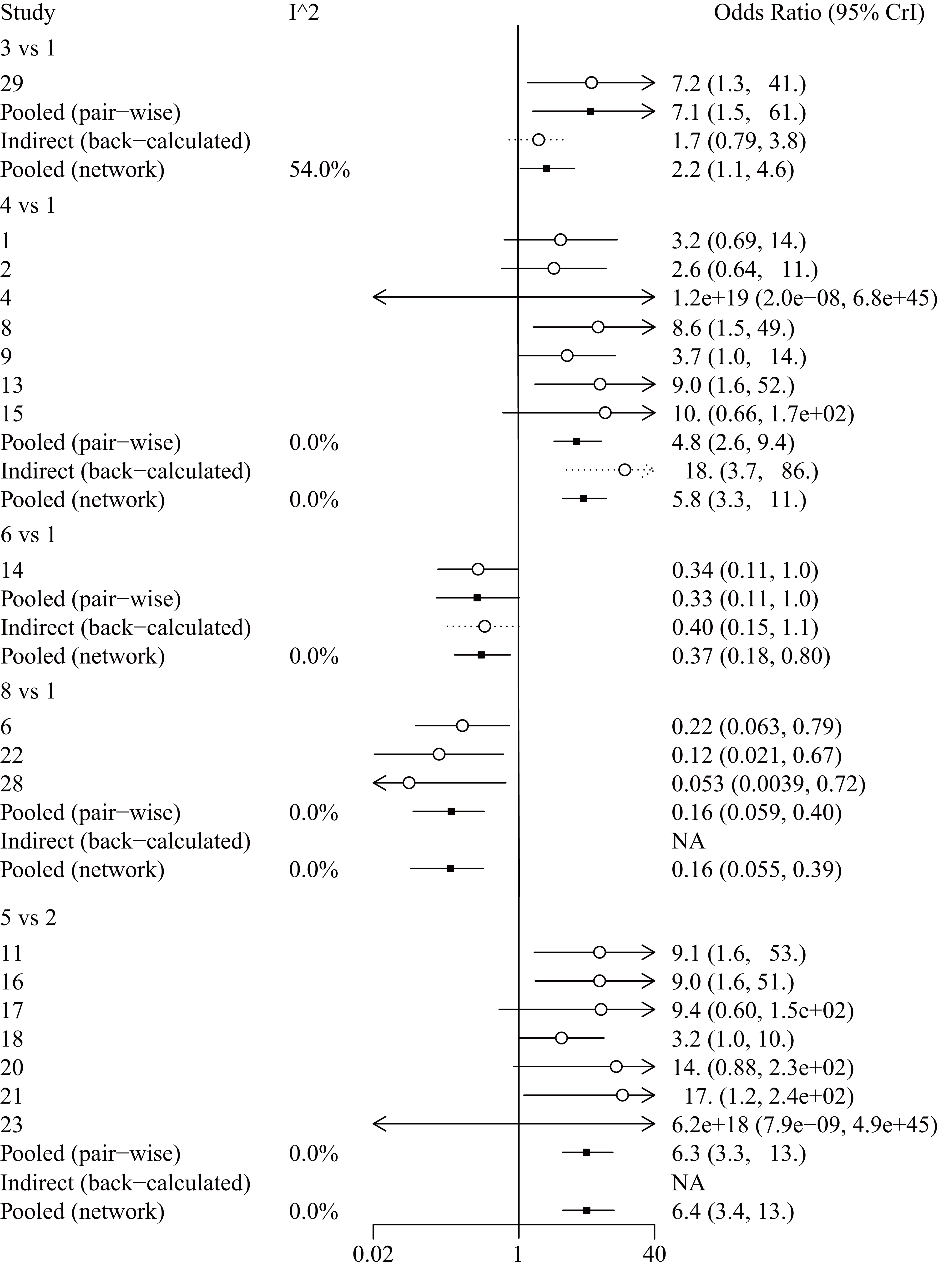


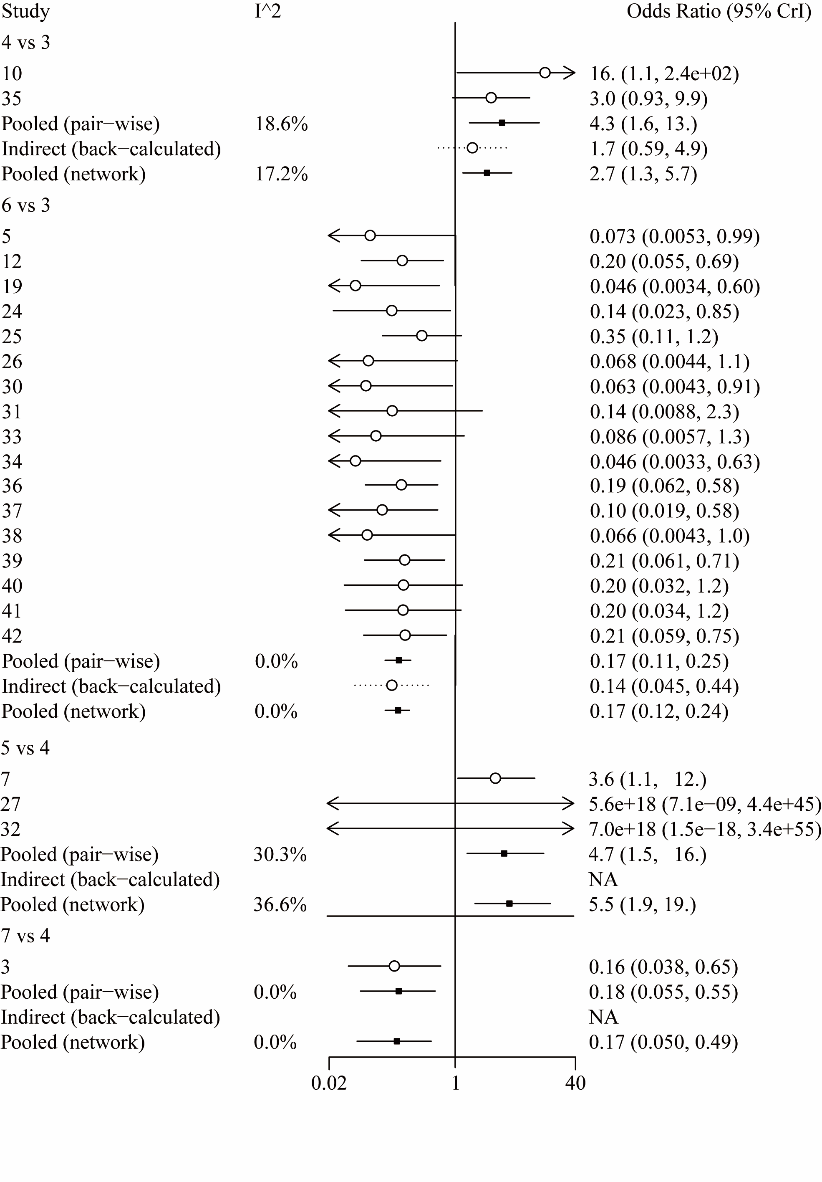


### S5.1.3 NMA including trials with participants' age <18.

(1) Node-splitting analysis of inconsistency


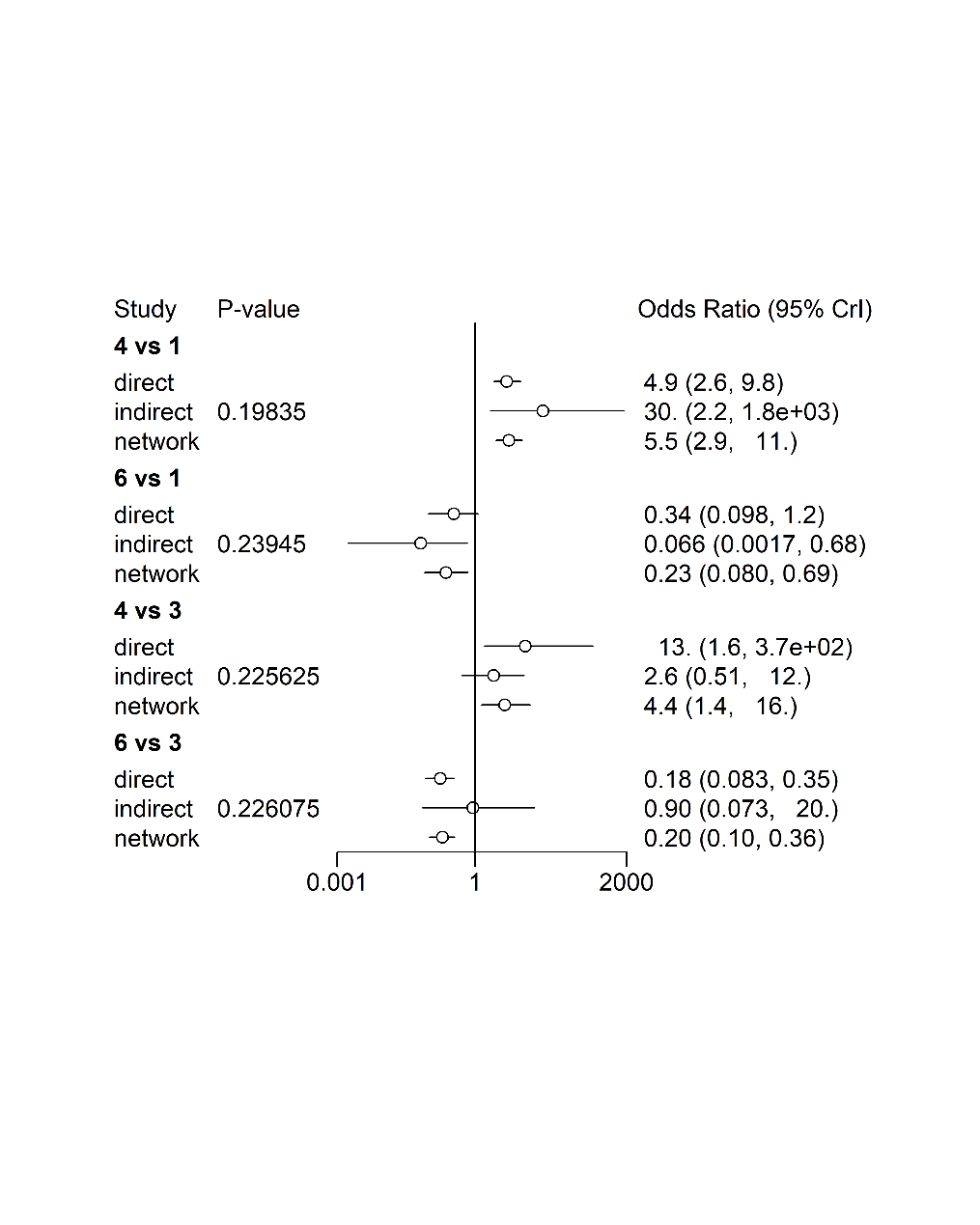


(2) Results of heterogeneity analysis


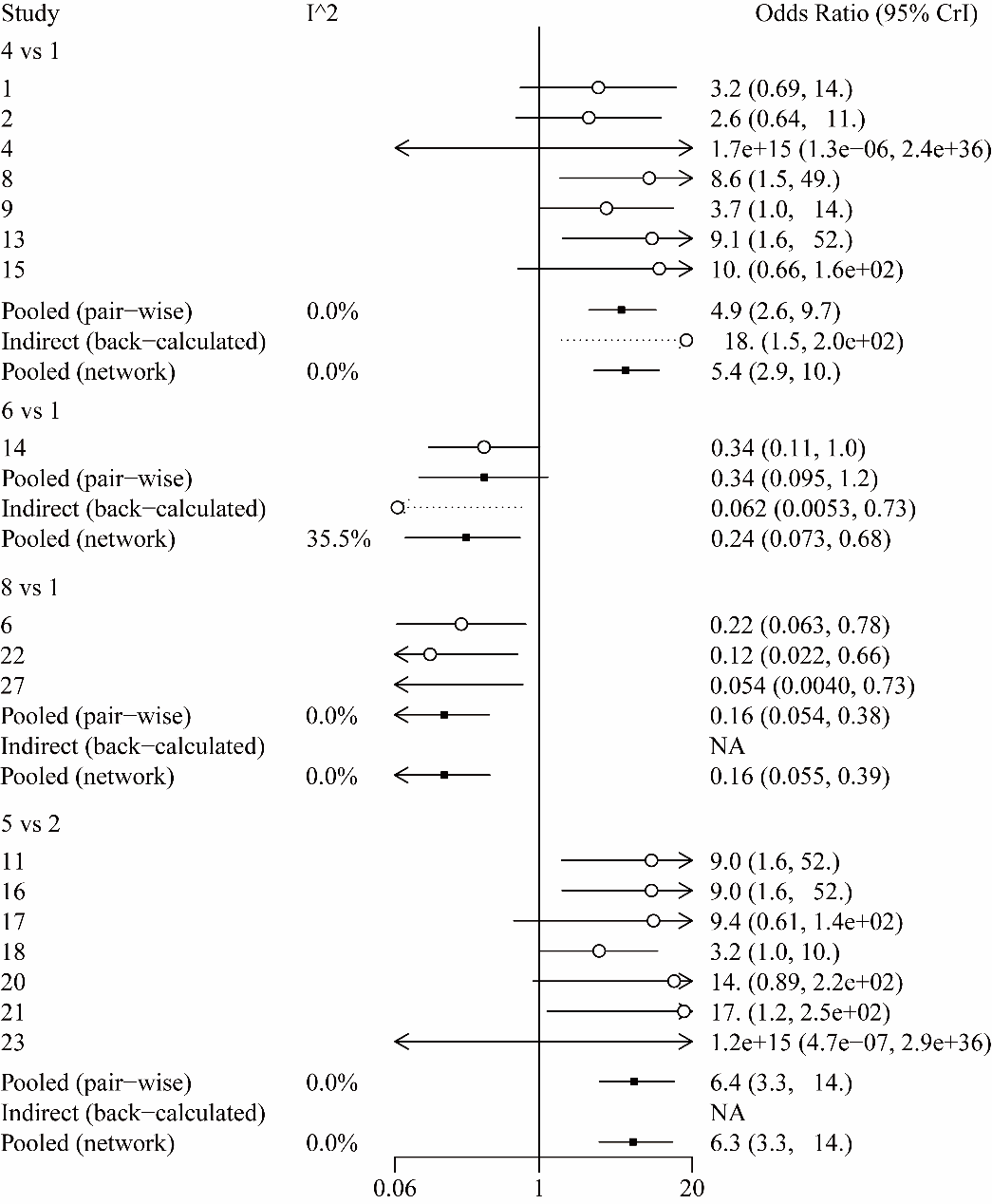


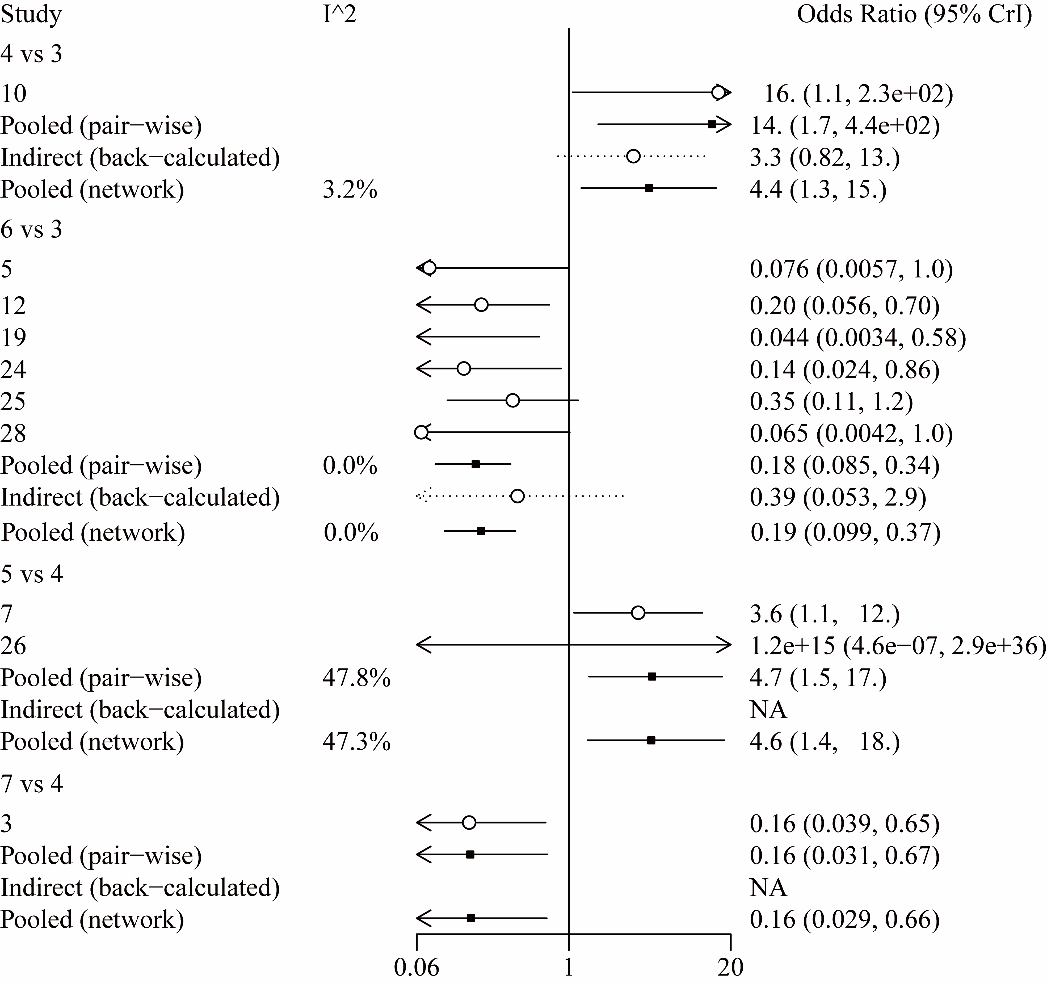


(3) The league table of effective rate

| **TG1.0** |  |  |  |  |  |  |  |
| --- | --- | --- | --- | --- | --- | --- | --- |
| 3.93  (0.88, 20) | **TG1.5** |  |  |  |  |  |  |
| 1.27  (0.38, 3.91) | 0.32  (0.05, 1.87) | **TG1.0+GC** |  |  |  |  |  |
| 5.38  (2.97, 10.54) | 1.39  (0.31, 5.48) | 4.25  (1.38, 14.88) | **TG1.0+TCM** |  |  |  |  |
| 25.34  (6.87, 112.9) | 6.41  (3.28, 13.76) | 20.02  (3.87, 125.8) | 4.65  (1.46, 17.98) | **TG1.5+TCM** |  |  |  |
| 0.25  (0.08, 0.73) | 0.06  (0.01, 0.38) | 0.2  (0.1, 0.37) | 0.05  (0.01, 0.14) | 0.01  (0, 0.05) | **GC** |  |  |
| 0.88  (0.15, 4.21) | 0.22  (0.02, 1.66) | 0.68  (0.09, 4.56) | 0.16  (0.03, 0.66) | 0.03  (0, 0.23) | 3.55  (0.47, 22.38) | **TCM** |  |
| 0.15  (0.06, 0.38) | 0.04  (0.01, 0.23) | 0.12  (0.03, 0.54) | 0.03  (0.01, 0.08) | 0.01  (0, 0.03) | 0.62  (0.15, 2.57) | 0.17  (0.03, 1.22) | **RT** |

The relative effects are measured as risk ratios along with 95%CI. Abbreviations: **TG1.0,** Tripterygium Glycosides (dose:1mg/Kg/day); **TG1.5,** Tripterygium Glycosides (dose:1.5mg/Kg/day); **TCM,** Traditional Chinese Medicine prescription or injection; **GC,** Glucocorticoids; **RT,** Routine treatment.

(4) The rank cumulative probability plot


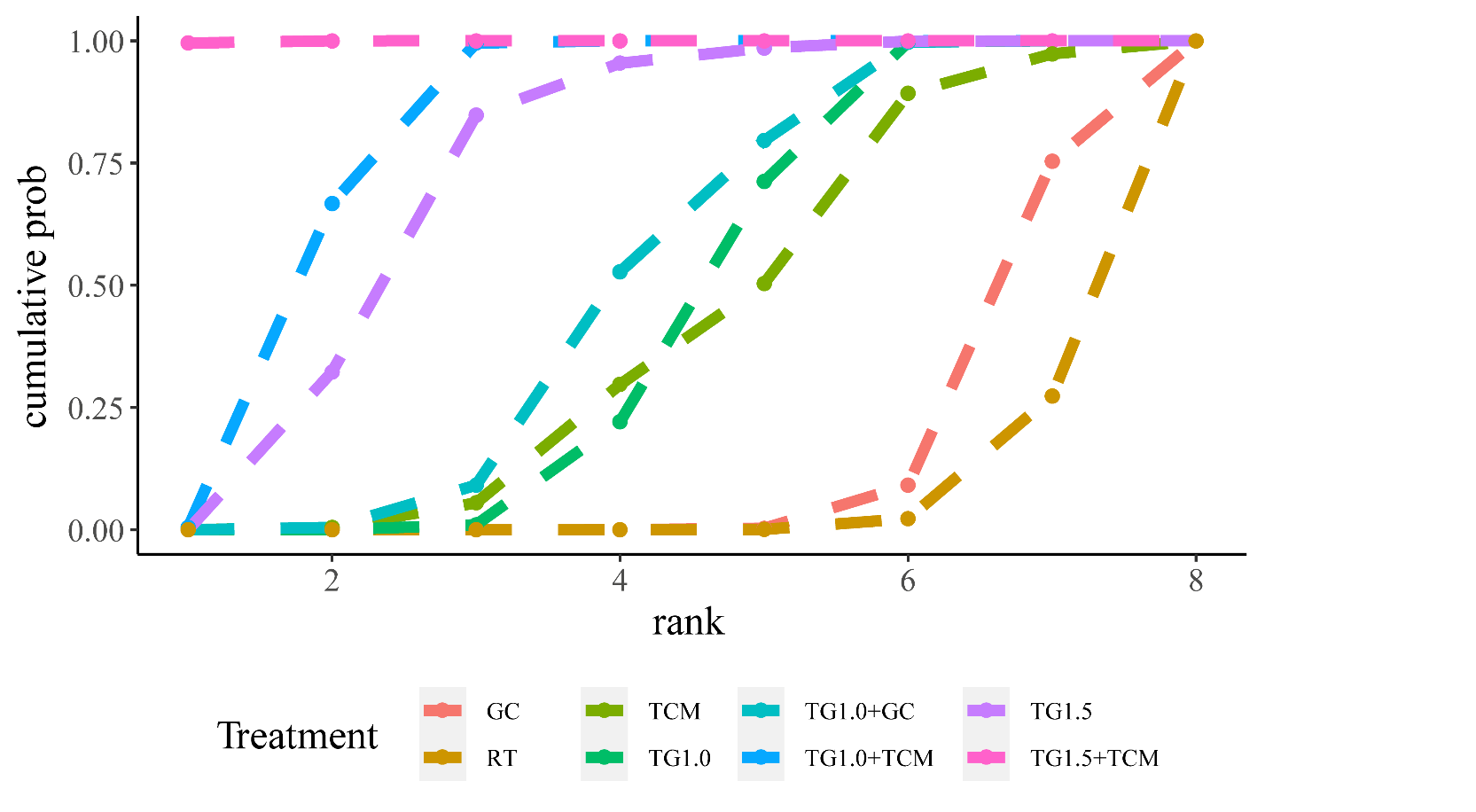


### S5.1.4 PMA including trials with participants' age >18.

(1) The forest plot


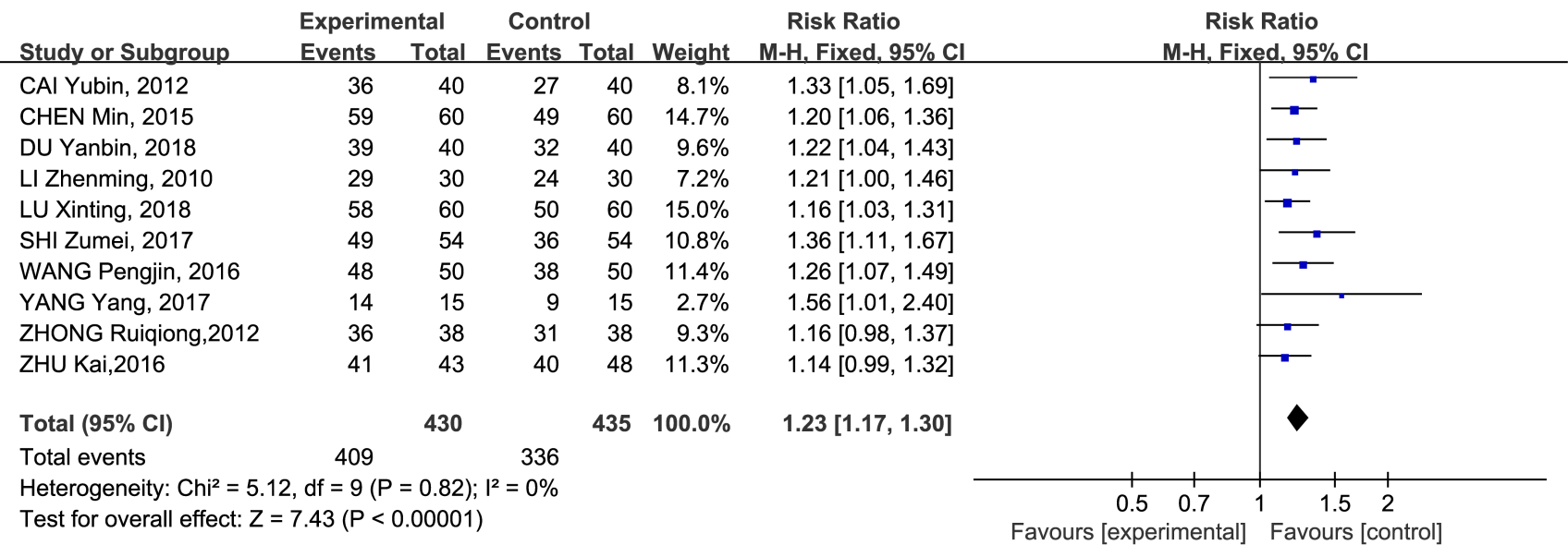


(2) The funnel plot


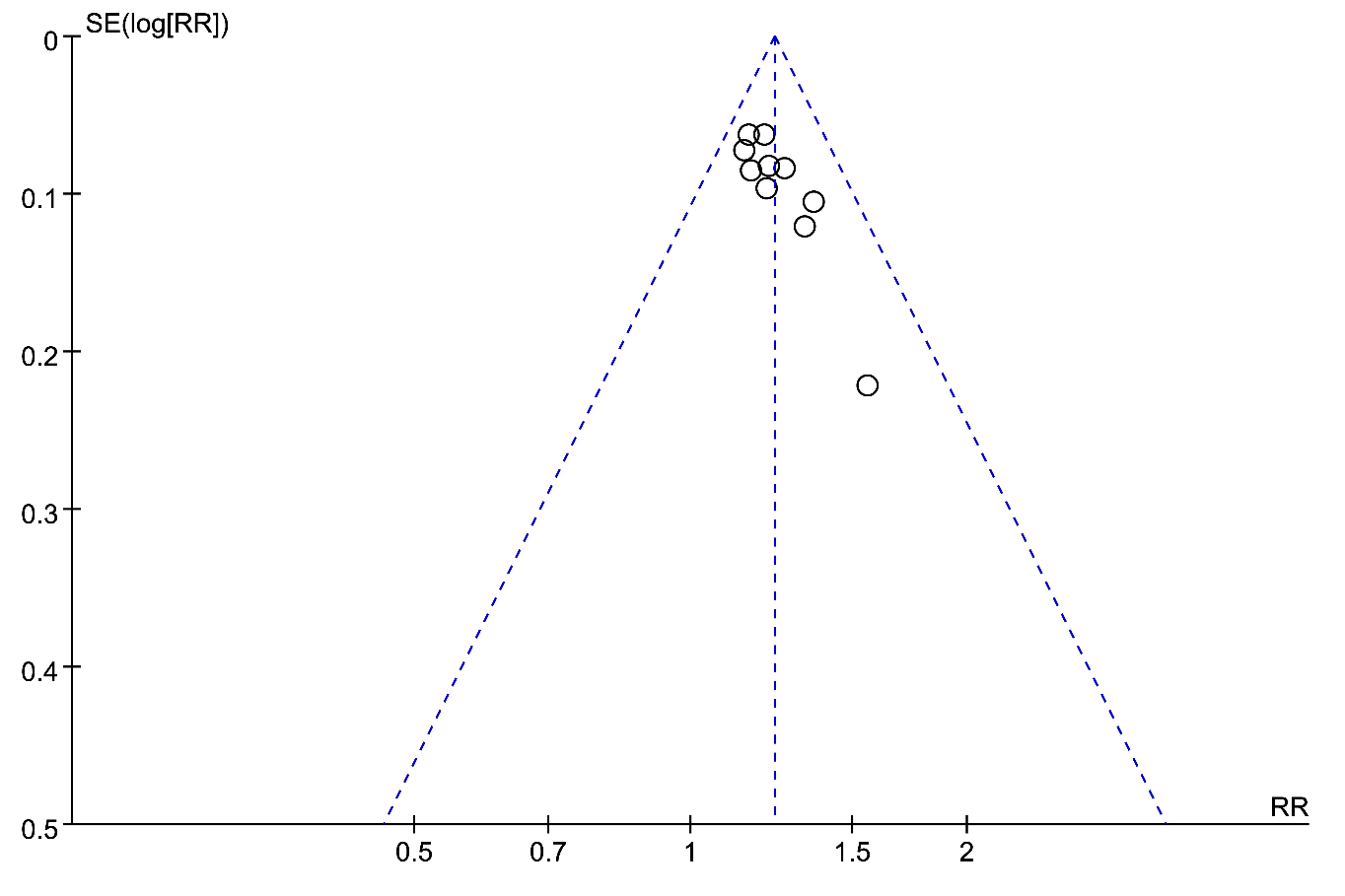


## S5.2 Recurrence rate

### Fig. S5.2.1 Results of heterogeneity analysis


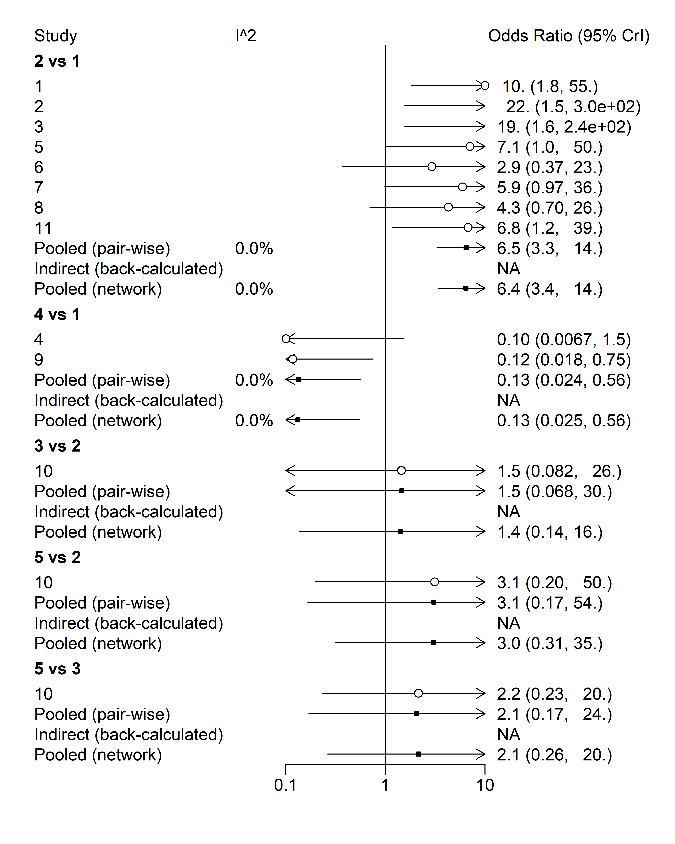


### Fig. S5.2.2 The funnel plot


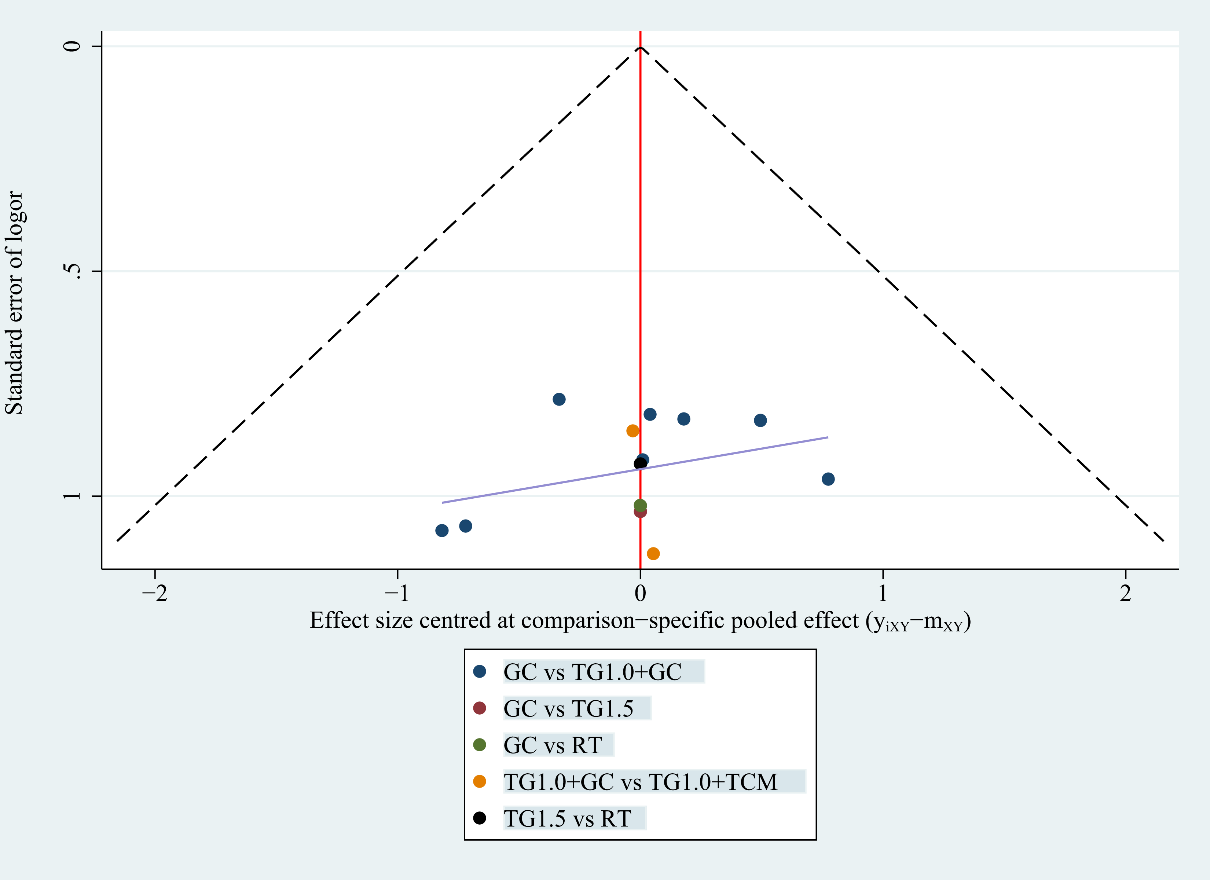


### S5.2.3 PMA including trials with participants' age >18.

1. The forest plot


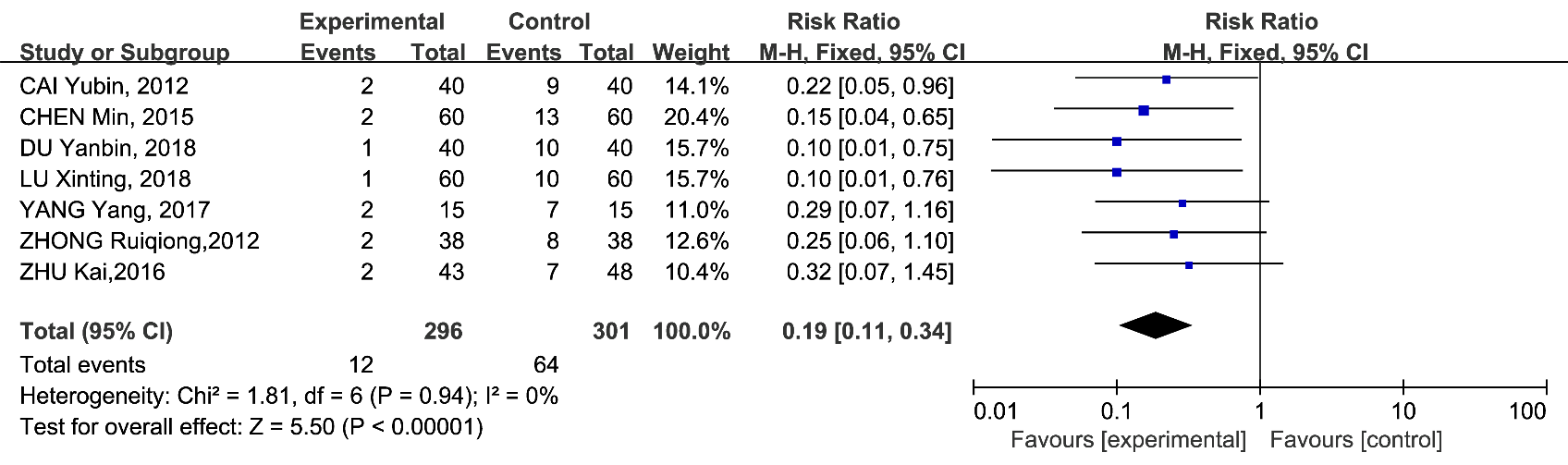


(2) The funnel plot


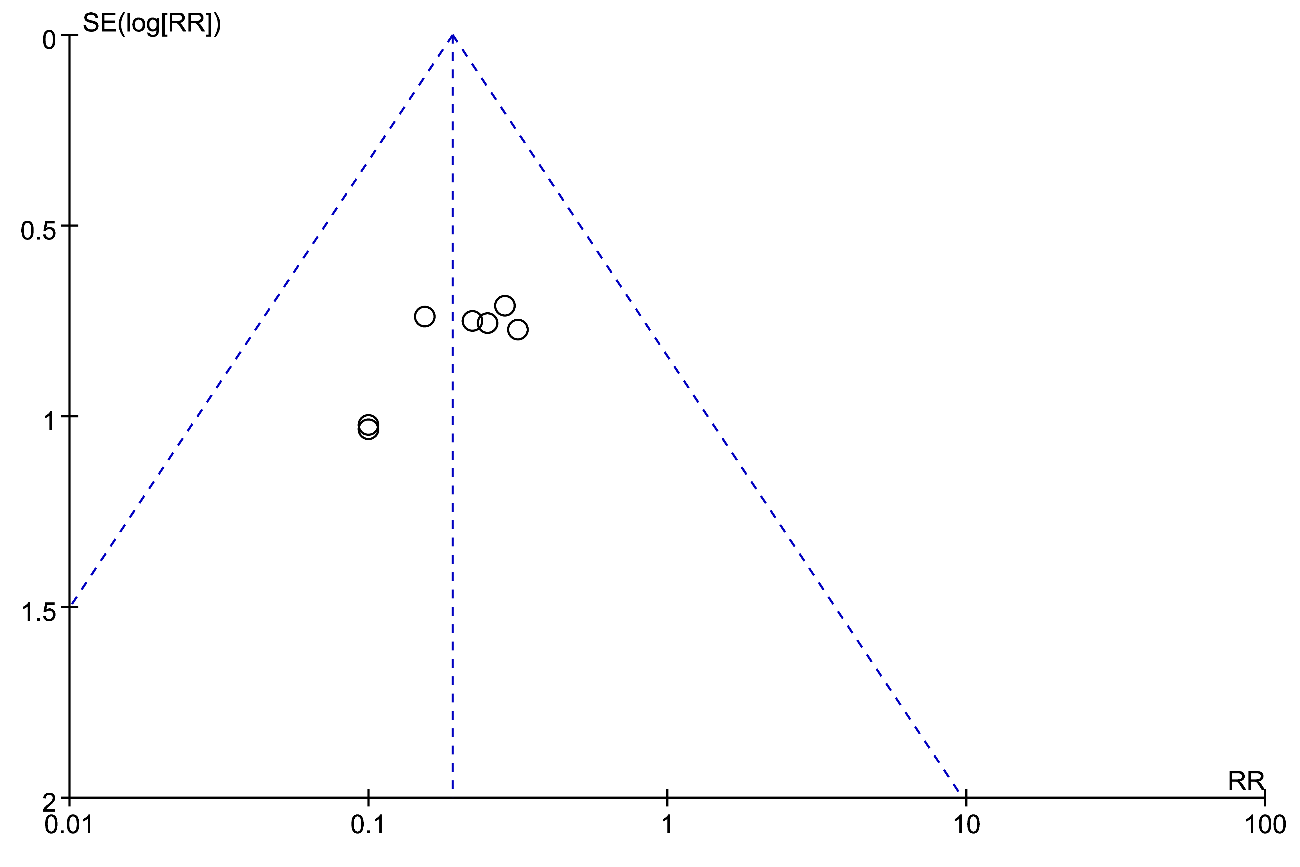


## S5.3 Liver injury events

### Fig. S5.3.1 Results of heterogeneity analysis


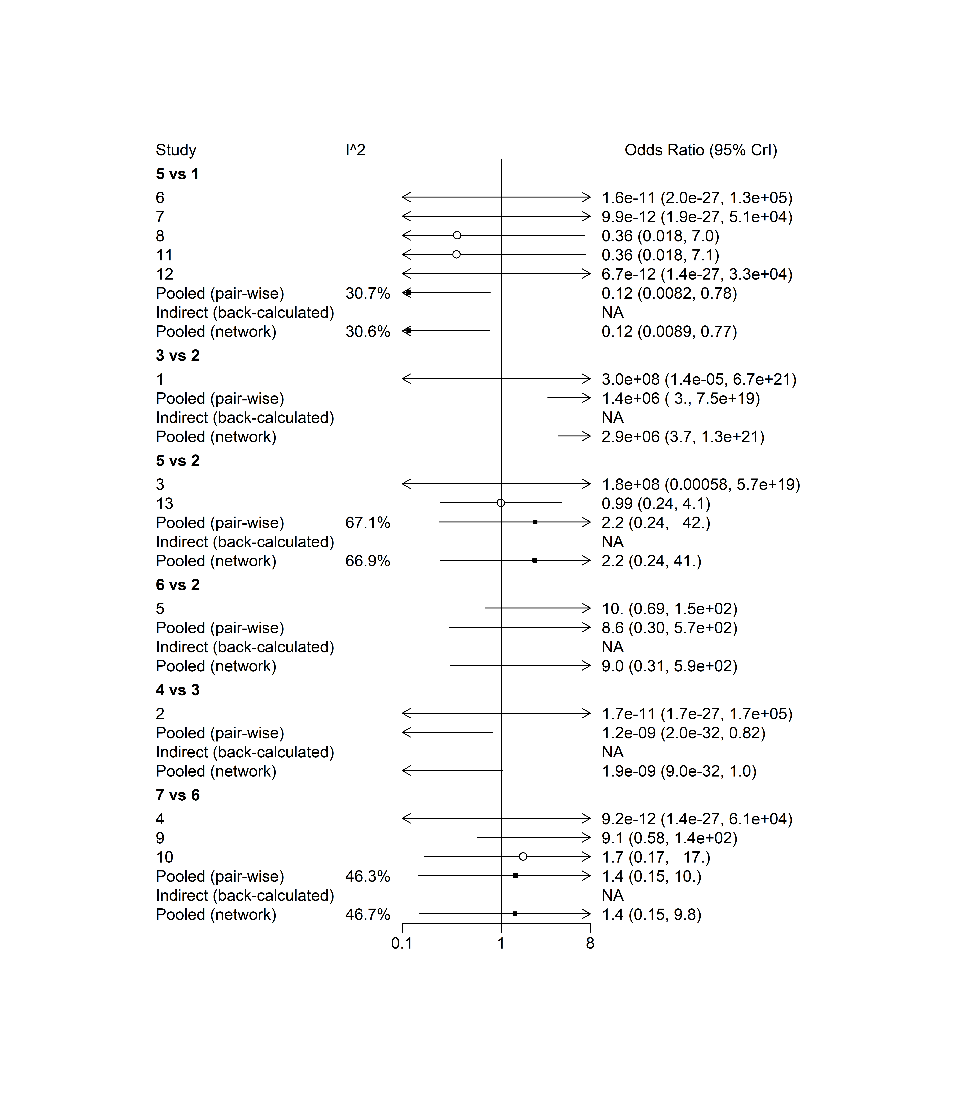


### Table S5.3.2 The league table of liver injury events

| **TG1.0** |  |  |  |  |  |  |
| --- | --- | --- | --- | --- | --- | --- |
| 0  (0, 0.65) | **RT** |  |  |  |  |  |
| 0  (0, 0.7) | 62.1  (0, 3.738e+23) | **TG1.5+TCM** |  |  |  |  |
| 0  (0, 4.03) | 222.4  (0, 1.509e+24) | 3.87  (0.04, 383.2) | **TG1.0+GC** |  |  |  |
| 0  (0, 6.66) | 297.5  (0, 2.233e+24) | 5.51  (0.03, 751.3) | 1.41  (0.15, 9.9) | **GC** |  |  |
| 0  (0, 0.23) | 24.36  (0, 1.199e+23) | 0.45  (0.02, 4.08) | 0.11  (0, 3.49) | 0.08  (0, 5.35) | **TG1.0+TCM** |  |
| 0  (0, 9.02) | 584.3  (0, 3.694e+24) | 8.69  (1.31, 116.5) | 2.28  (0.02, 512.1) | 1.62  (0.01, 693.4) | 20.23  (1.11, 1160) | **TG1.5** |

The relative effects are measured as risk ratios along with 95%CI. Abbreviations: **TG1.0,** Tripterygium Glycosides (dose:1mg/Kg/day); **TG1.5,** Tripterygium Glycosides (dose:1.5mg/Kg/day); **TCM,** Traditional Chinese Medicine prescription or injection; **GC,** Glucocorticoids; **RT,** Routine treatment.

### Fig. S5.3.3 The forest plot


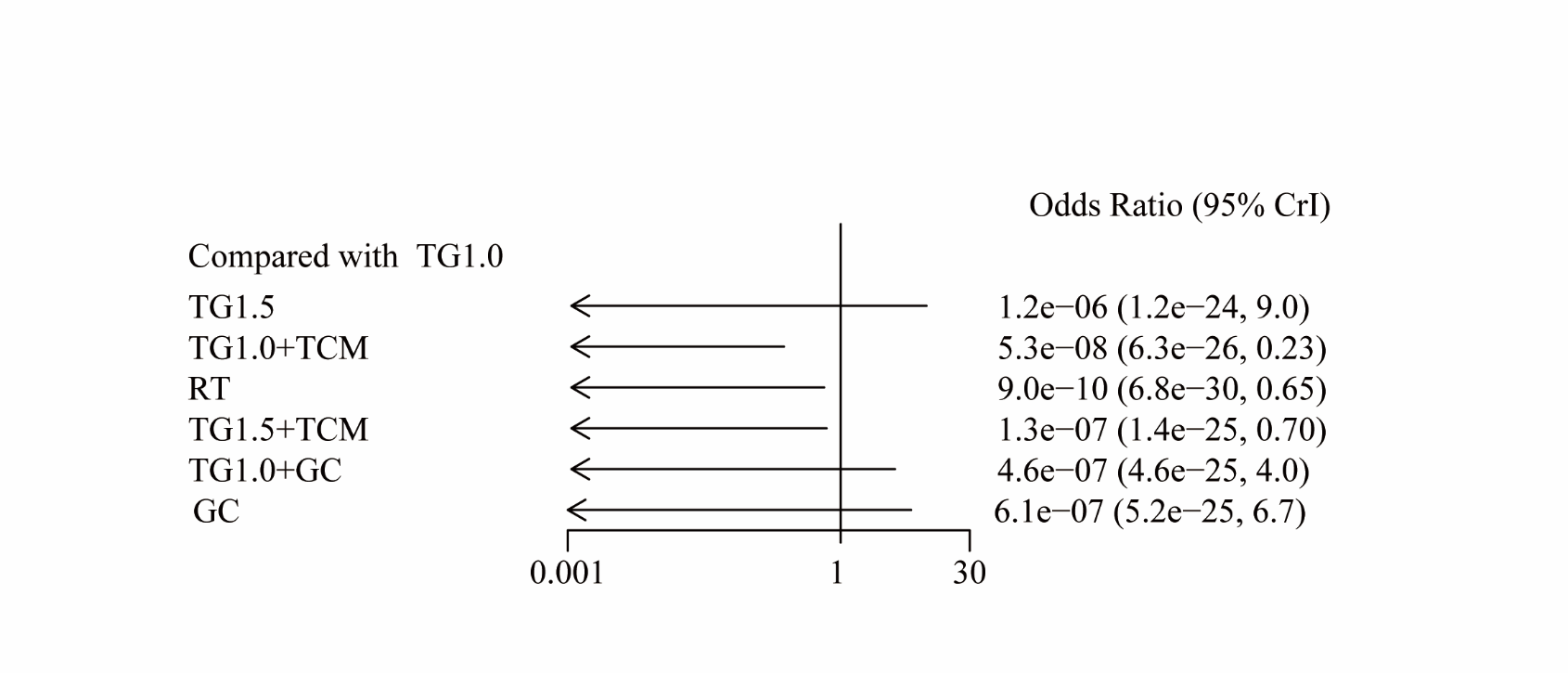


### Fig. S5.3.4 The funnel plot


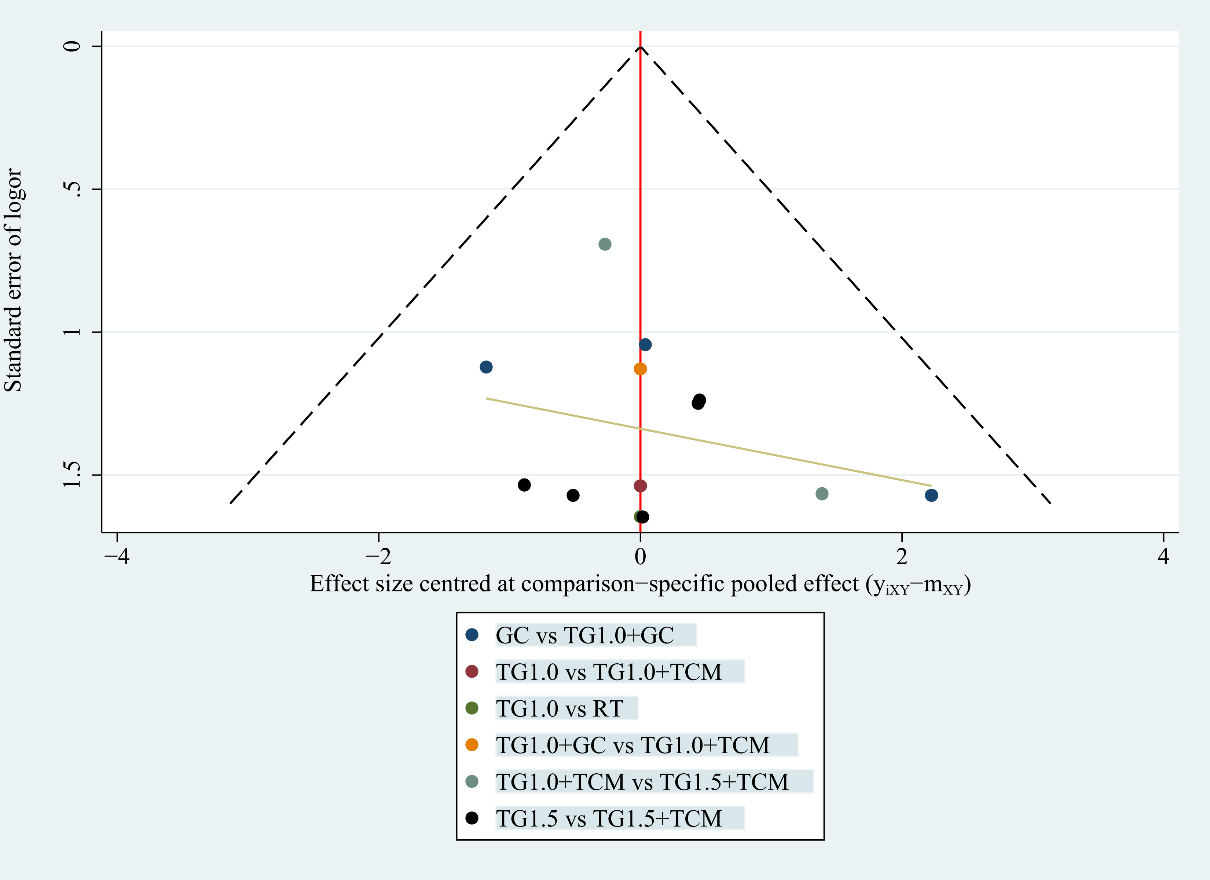


### S5.3.5 NMA including trials with participants' age <18.

1. Results of heterogeneity analysis


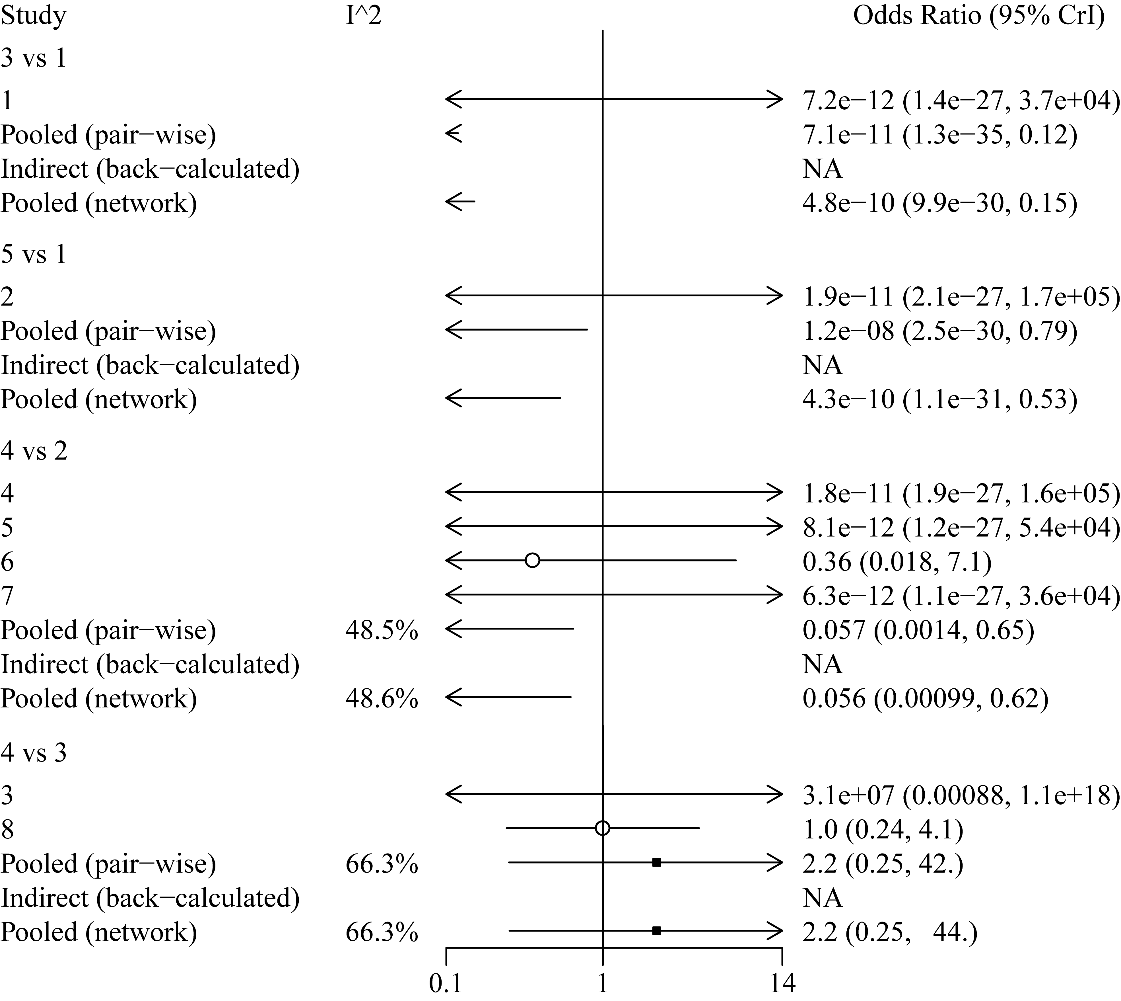


1. The league table of liver injury events

| **TG1.0** |  |  |  |  |
| --- | --- | --- | --- | --- |
| 0  (0, 12.2) | **TG1.5** |  |  |  |
| 0  (0, 0.11) | 0.02  (0, 0.59) | **TG1.0+TCM** |  |  |
| 0  (0, 0.35) | 0.06  (0, 0.59) | 2.17  (0.25, 41.51) | **TG1.5+TCM** |  |
| 0  (0, 0.84) | 0.01  (0, 7.795e+20) | 0.72  (0, 4.001e+22) | 0.3  (0, 1.783e+22) | **RT** |

The relative effects are measured as risk ratios along with 95%CI. Abbreviations: **TG1.0,** Tripterygium Glycosides (dose:1mg/Kg/day); **TG1.5,** Tripterygium Glycosides (dose:1.5mg/Kg/day); **TCM,** Traditional Chinese Medicine prescription or injection; **RT,** Routine treatment.

1. The forest plot


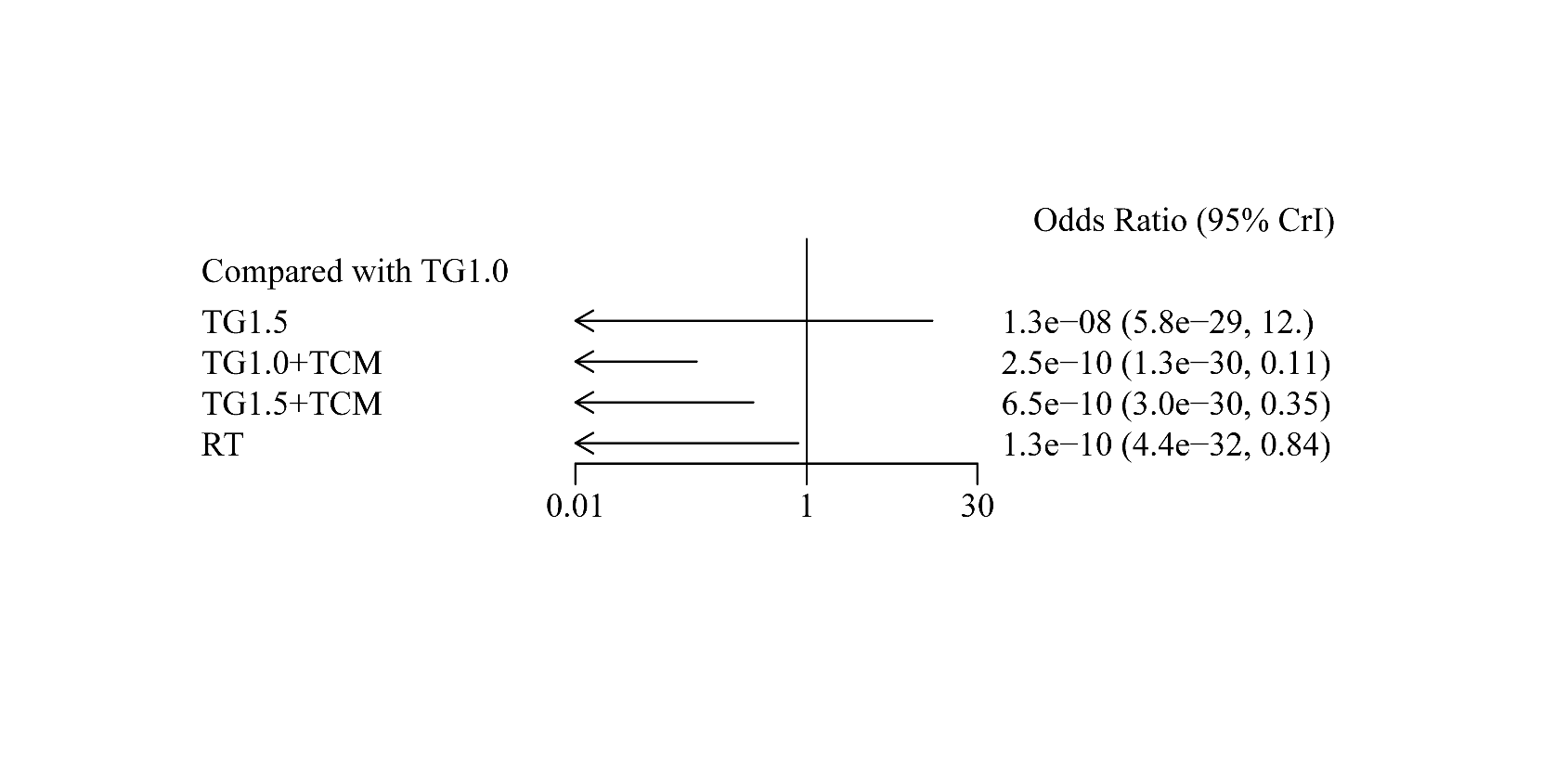


1. The rank cumulative probability plot


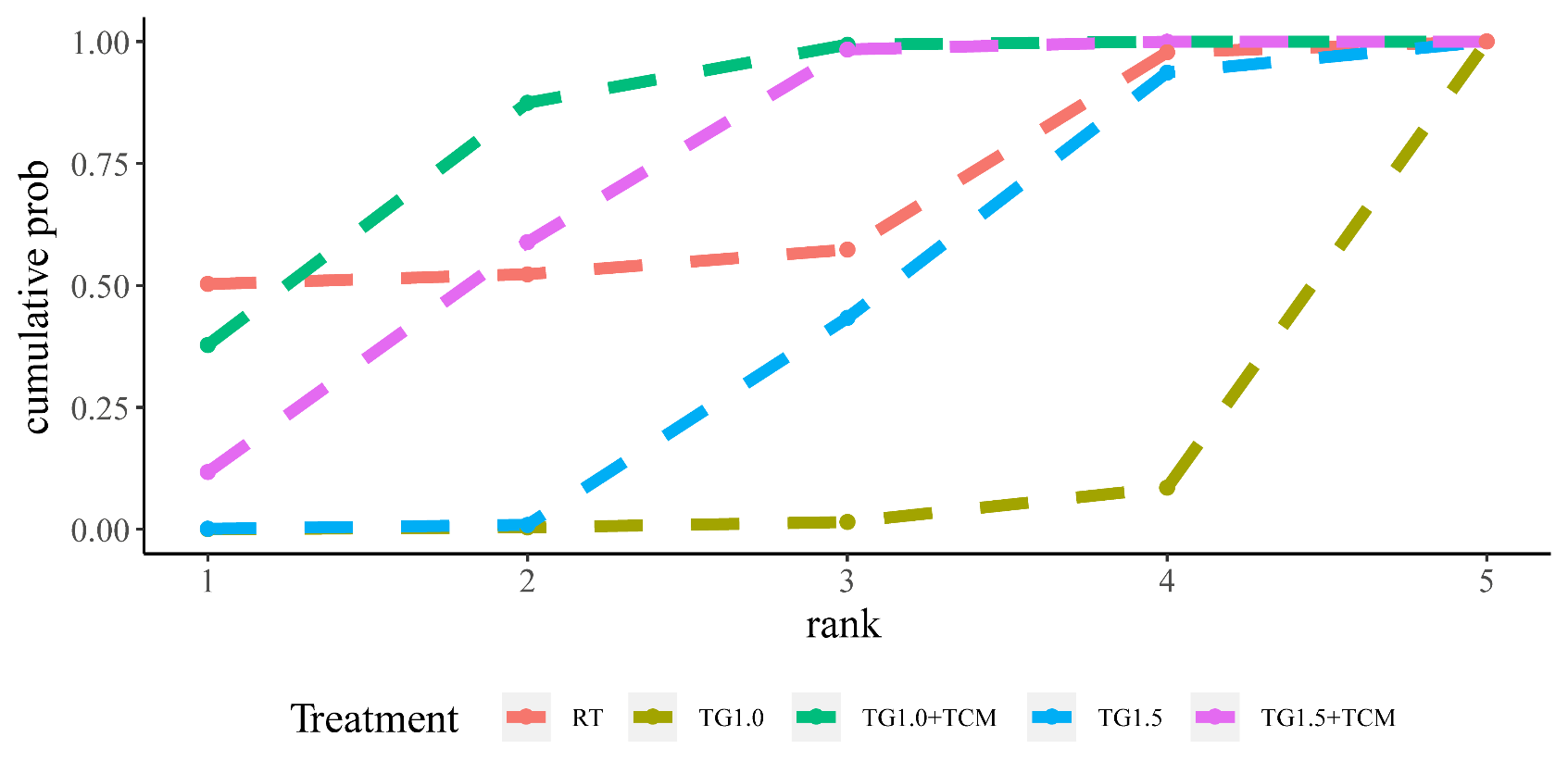


## S5.4 Leukopenia events

### Fig. S5.4.1 Results of heterogeneity analysis


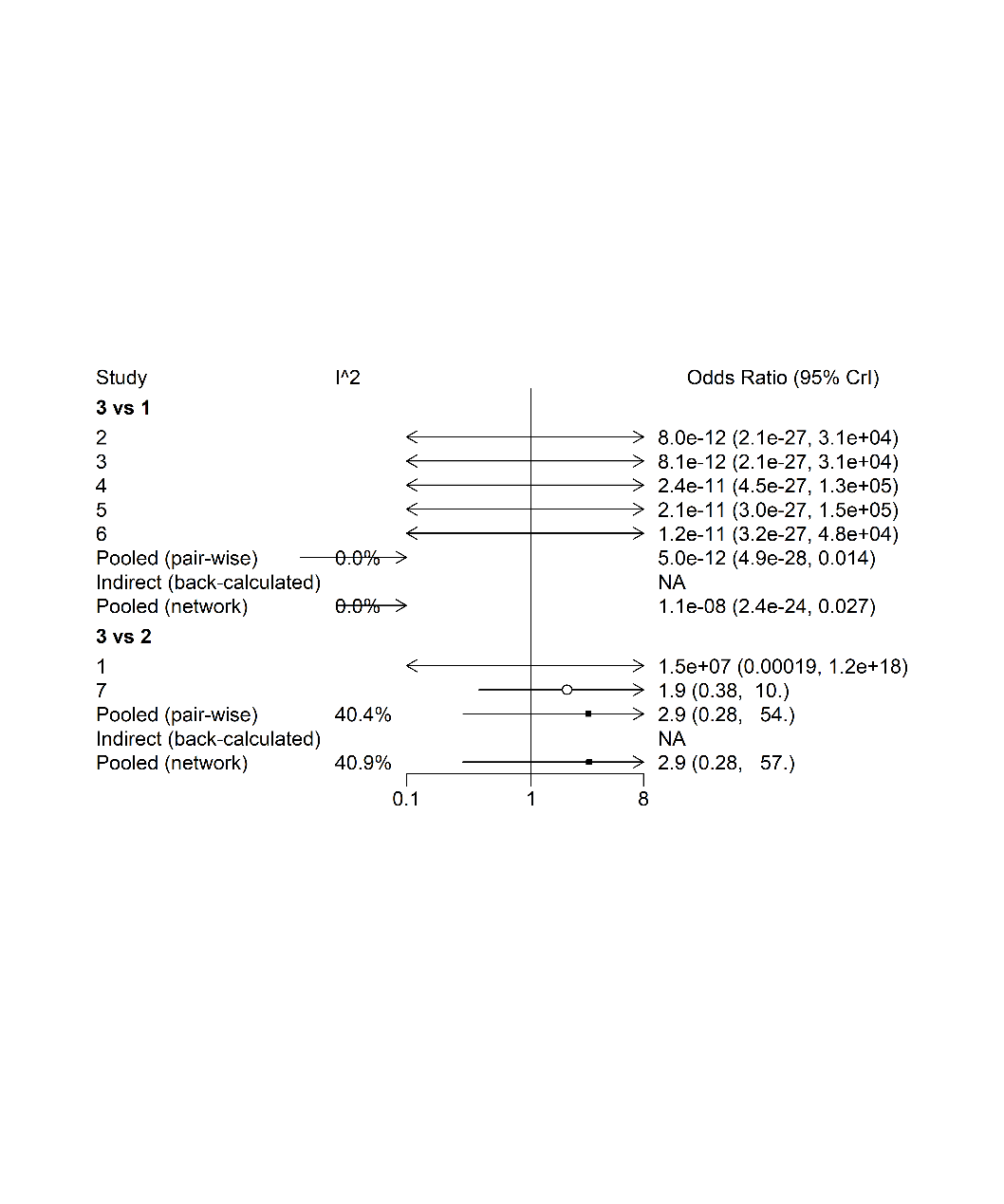


### Table S5.4.2 The league table of leukopenia events

| **TG1.5** |  |  |
| --- | --- | --- |
| 0  (0, 0.01) | **TG1.0+TCM** |  |
| 0  (0, 0.02) | 2.86  (0.28, 54.92) | **TG1.5+TCM** |

The relative effects are measured as risk ratios along with 95%CI. Abbreviations: **TG1.0,** Tripterygium Glycosides (dose:1mg/Kg/day); **TG1.5,** Tripterygium Glycosides (dose:1.5mg/Kg/day); **TCM,** Traditional Chinese Medicine prescription or injection.

### Fig. S5.4.3 The forest plot


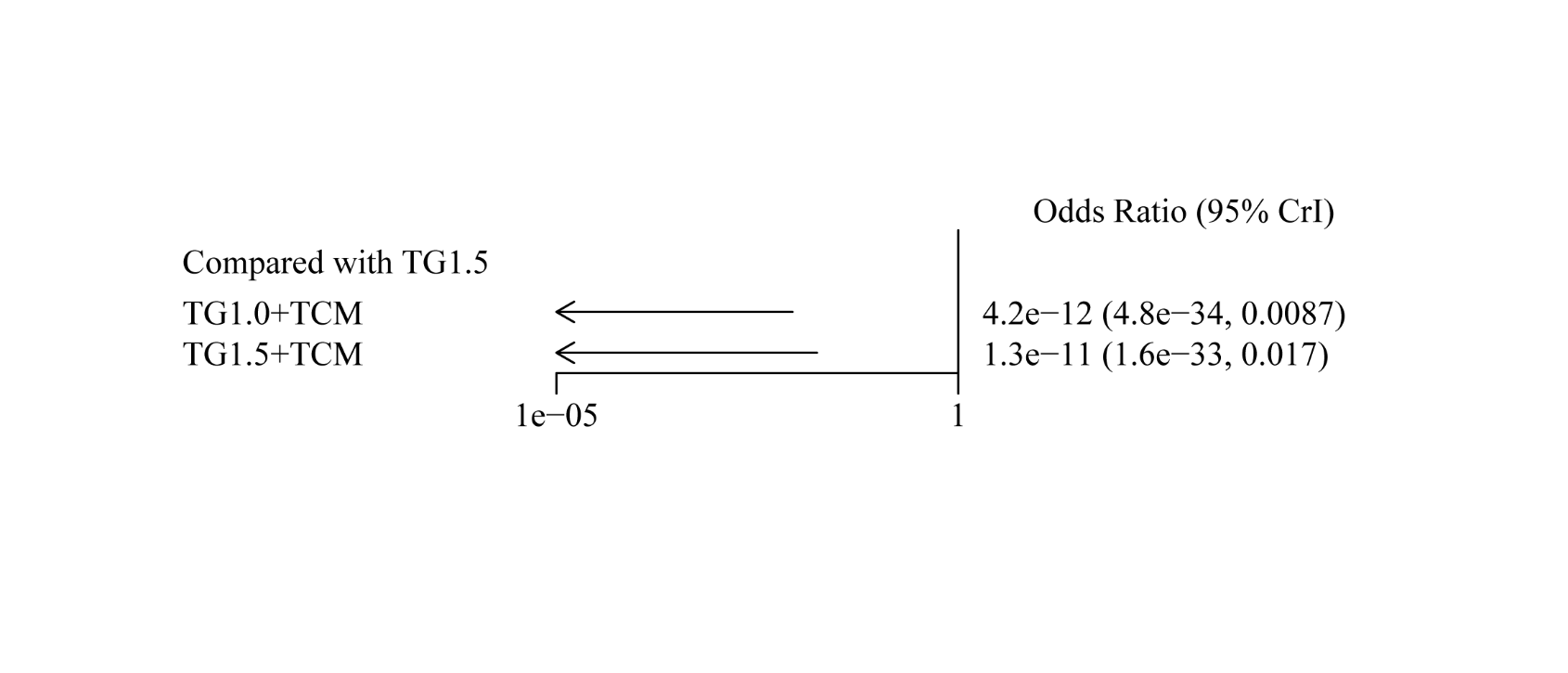


## S5.5 Gastrointestinal events

### Fig. S5.5.1 Node-splitting analysis of inconsistency


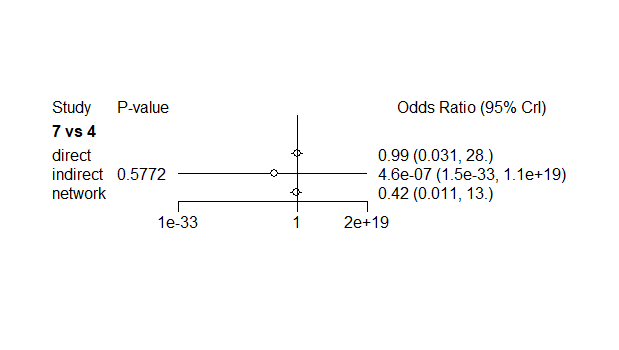


### Fig. S5.5.2 Results of heterogeneity analysis


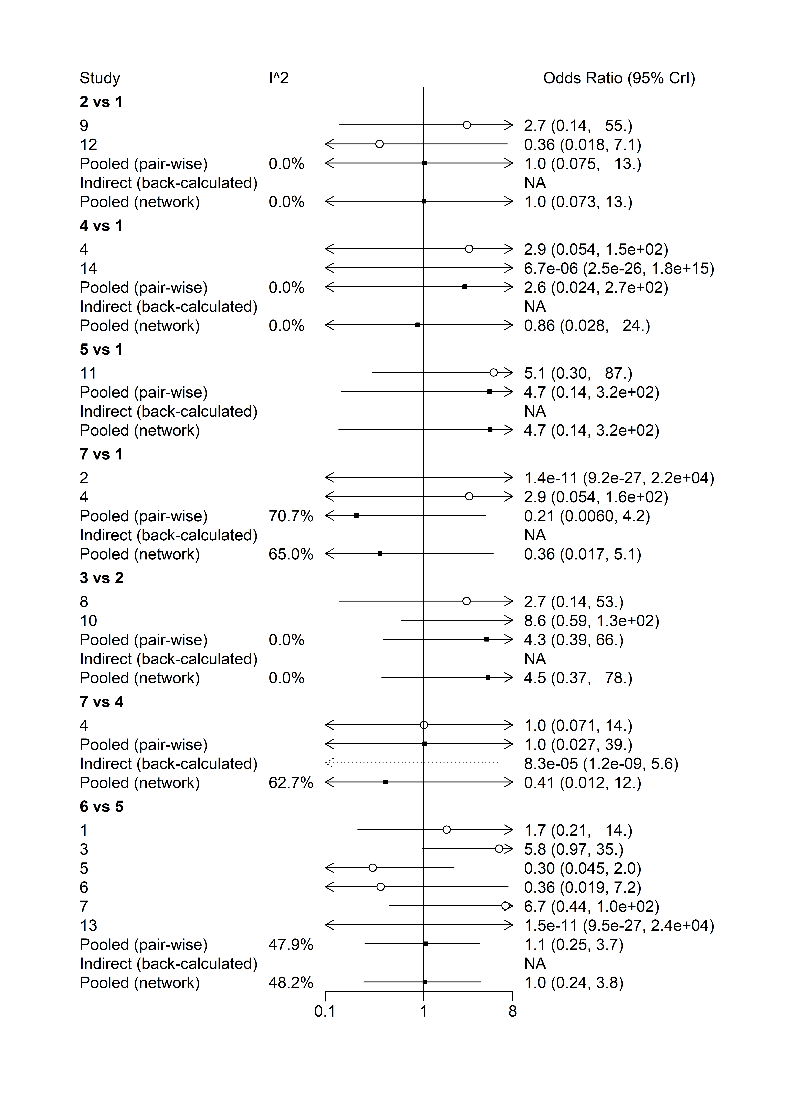


### Table S5.5.3 The league table of gastrointestinal events

| **TG1.0** |  |  |  |  |  |  |
| --- | --- | --- | --- | --- | --- | --- |
| 1.02  (0.08, 13.93) | **TG1.0+TCM** |  |  |  |  |  |
| 4.35  (0.38, 73.25) | 4.35  (0.12, 196.4) | **RT** |  |  |  |  |
| 0.88  (0.01, 61.27) | 0.86  (0.03, 24.54) | 0.2  (0, 27.69) | **TG1.5+TCM** |  |  |  |
| 4.89  (0.06, 633.7) | 4.67  (0.14, 291.8) | 1.1  (0.01, 254.7) | 5.58  (0.04, 1239) | **TG1.0+GC** |  |  |
| 5.05  (0.05, 751.8) | 4.85  (0.1, 352.9) | 1.14  (0, 300.3) | 5.75  (0.03, 1452) | 1.04  (0.24, 3.8) | **GC** |  |
| 0.37  (0.01, 15.38) | 0.37  (0.02, 5.21) | 0.08  (0, 7.38) | 0.41  (0.01, 12.84) | 0.08  (0, 6.36) | 0.07  (0, 8.03) | **TCM** |

The relative effects are measured as risk ratios along with 95%CI. Abbreviations: **TG1.0,** Tripterygium Glycosides (dose:1mg/Kg/day); **TG1.5,** Tripterygium Glycosides (dose:1.5mg/Kg/day); **TCM,** Traditional Chinese Medicine prescription or injection; **GC,** Glucocorticoids; **RT,** Routine treatment.

### Fig. S5.5.4 The forest plot


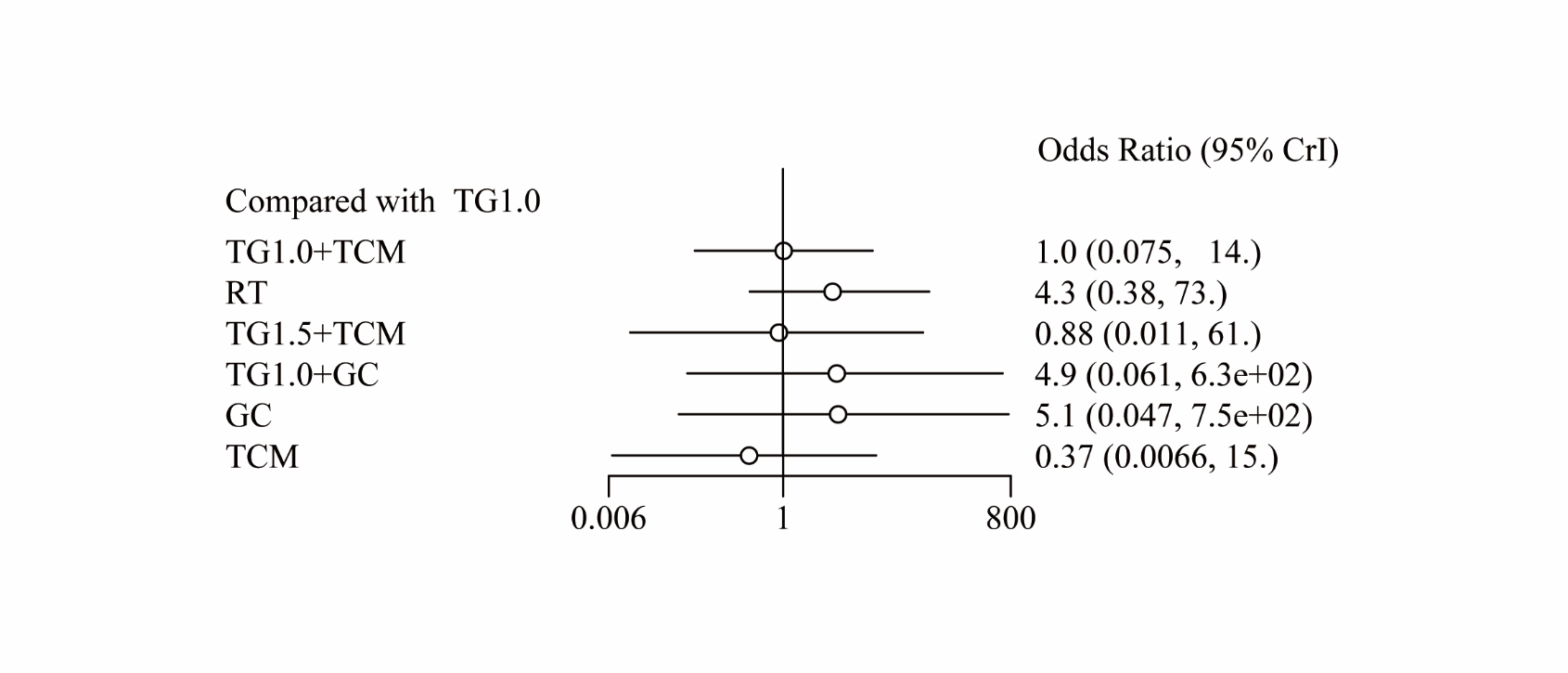


### Fig. S5.5.5 The funnel plot


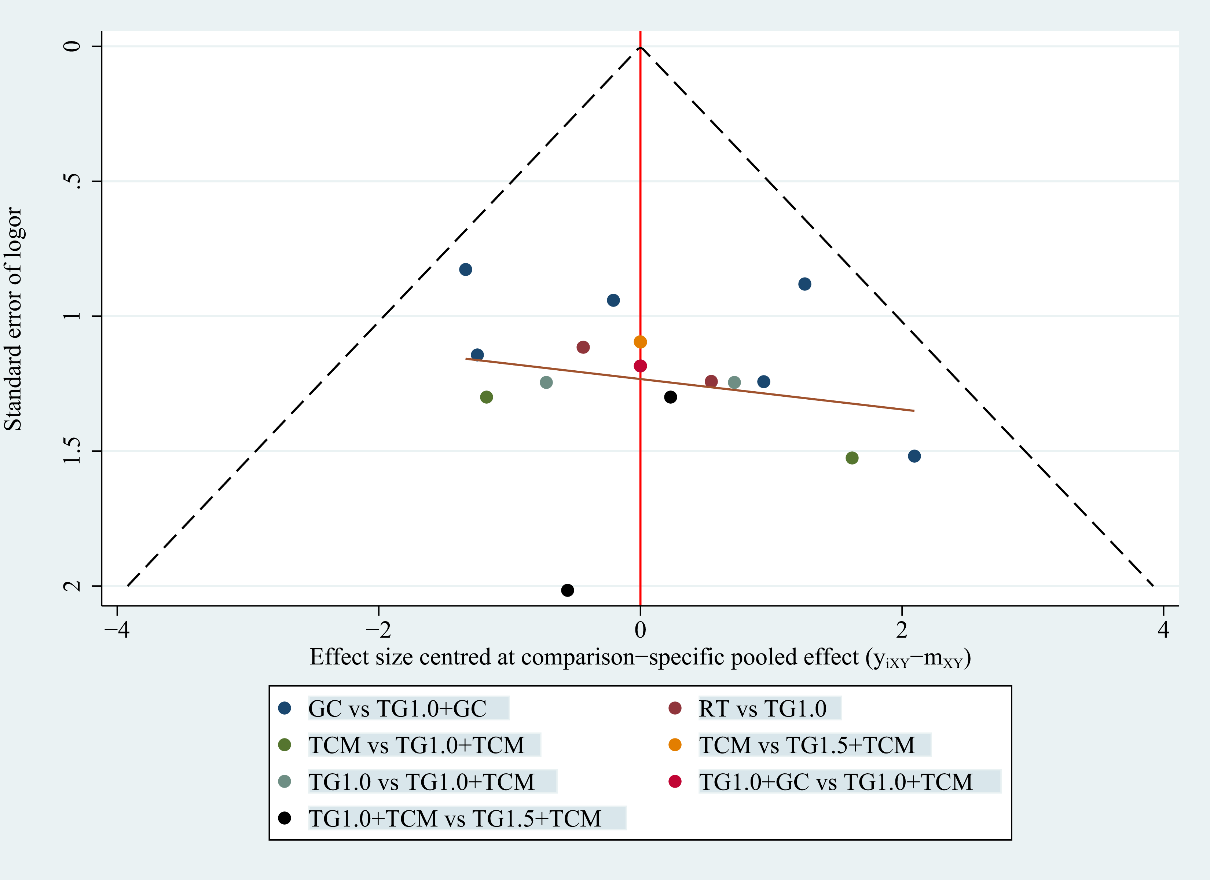


### S5.5.6 NMA including trials with participants' age <18.

1. Results of heterogeneity analysis


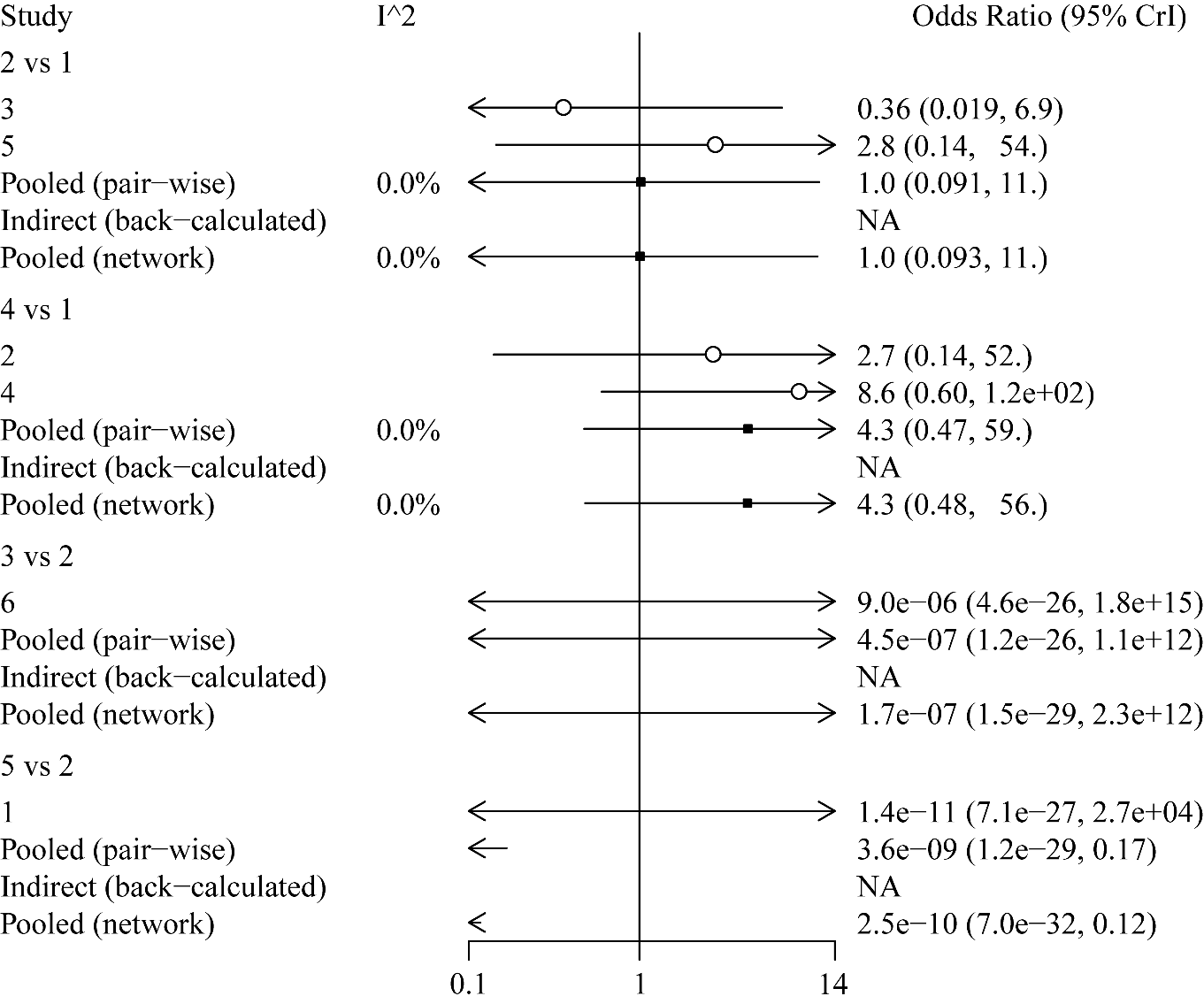


1. The league table of gastrointestinal events

| **TG1.0** |  |  |  |  |
| --- | --- | --- | --- | --- |
| 0.98  (0.09, 10.33) | **TG1.0+TCM** |  |  |  |
| 0  (0, 1.039e+13) | 0  (0, 8.604e+12) | **TG1.5+TCM** |  |  |
| 4.22  (0.47, 53.58) | 4.35  (0.17, 135.8) | 2.624e+05  (0, 5.175e+28) | **RT** |  |
| 0  (0, 0.17) | 0  (0, 0.13) | 0  (0, 7.85e+20) | 0  (0, 0.05) | **TCM** |

The relative effects are measured as risk ratios along with 95%CI. Abbreviations: **TG1.0,** Tripterygium Glycosides (dose:1mg/Kg/day); **TG1.5,** Tripterygium Glycosides (dose:1.5mg/Kg/day); **RT,** Routine treatment; **TCM,** Traditional Chinese Medicine prescription or injection.

1. The forest plot


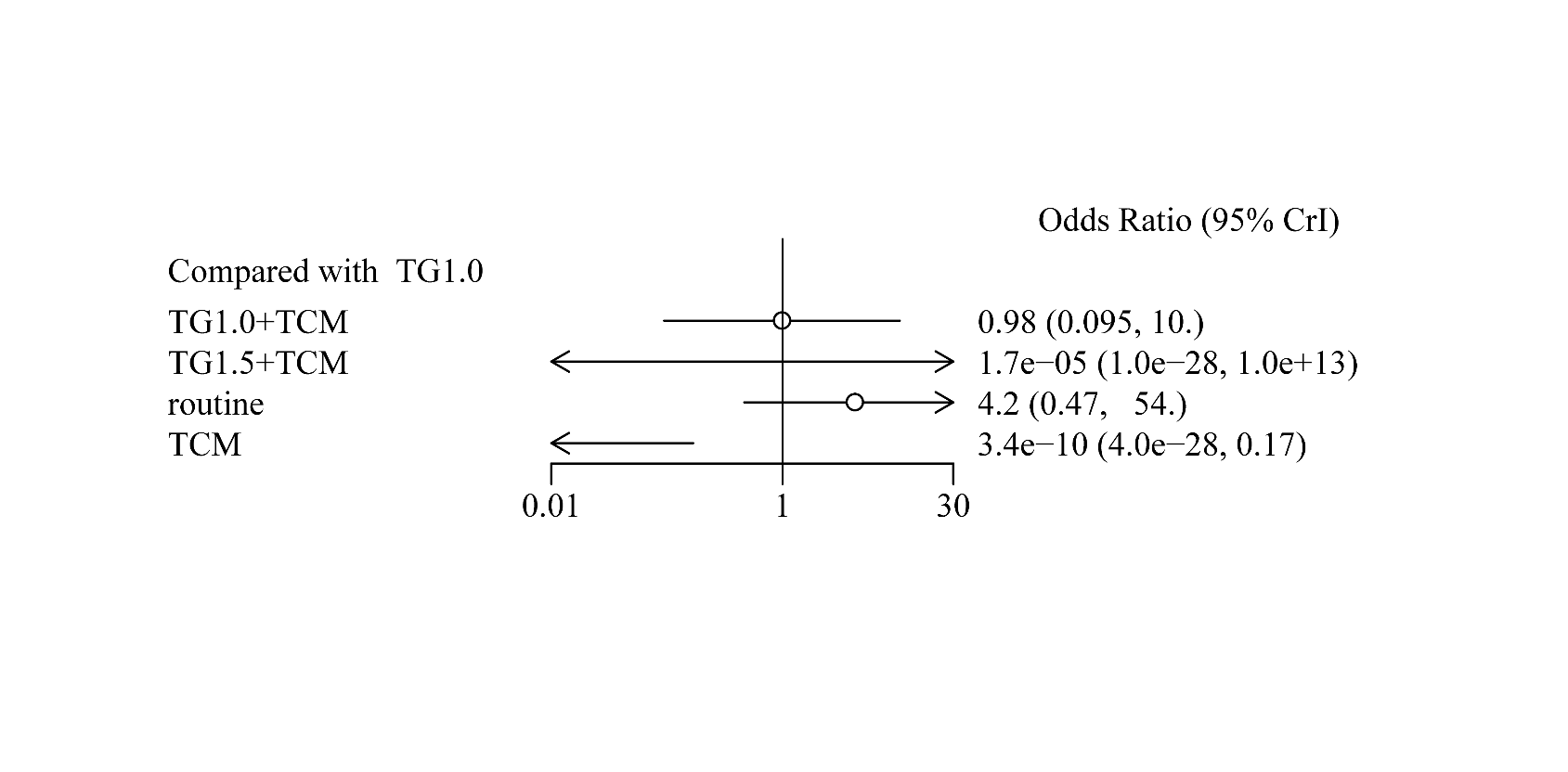


1. The rank cumulative probability plot


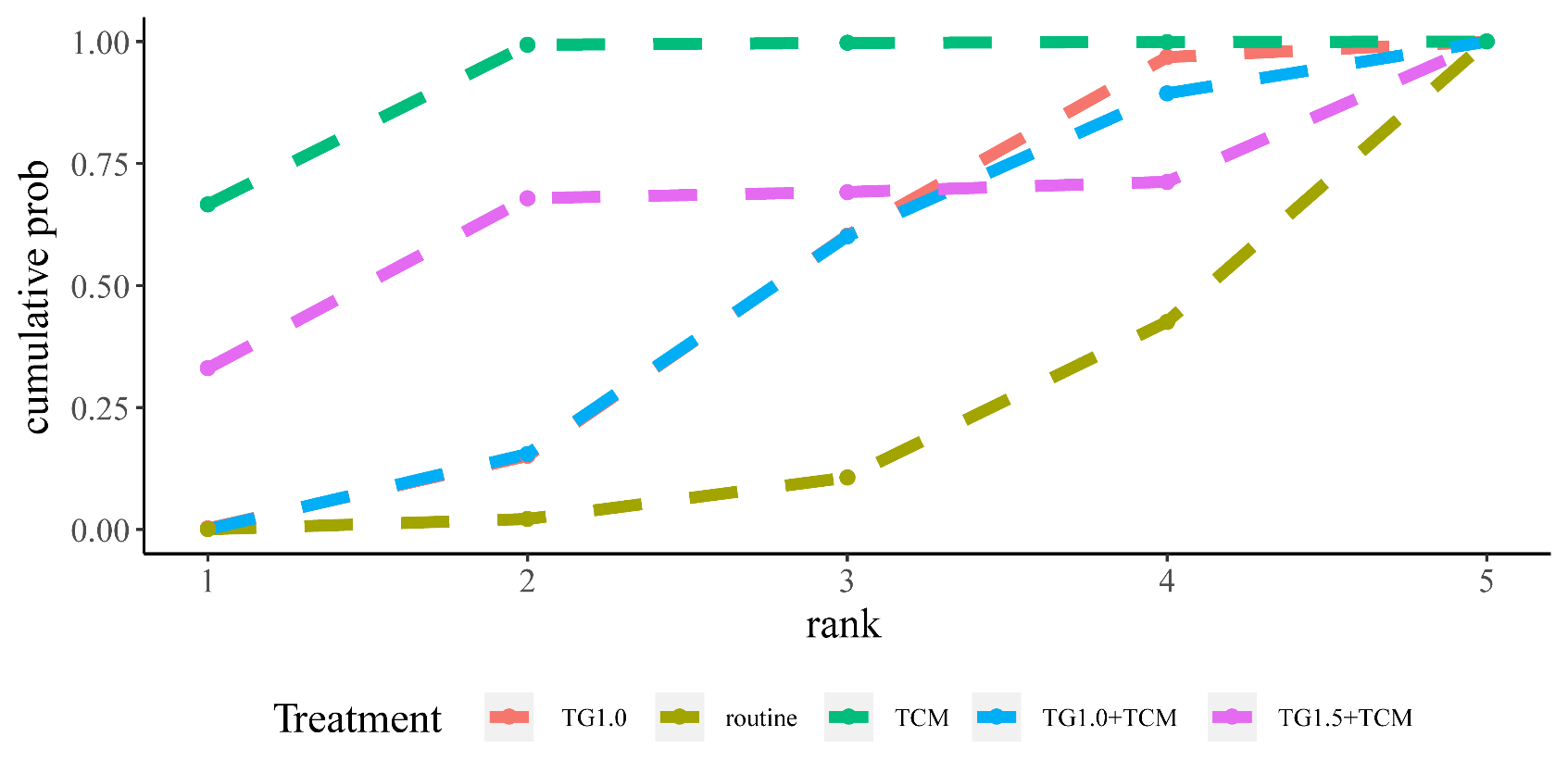


# S6. The clusterank plots

## Fig. S6.1 Effective rate and liver injury events


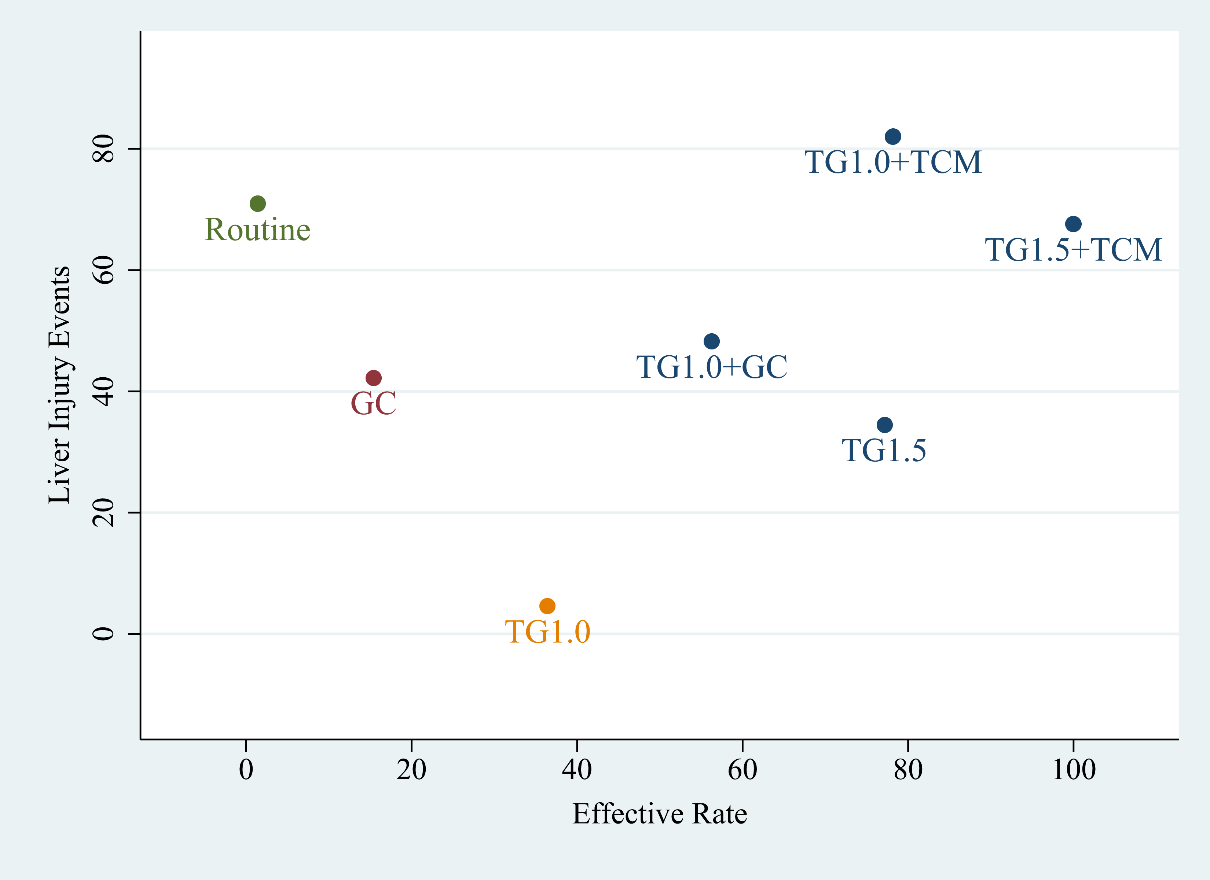


## Fig. S6.2 Effective rate and leukopenia events


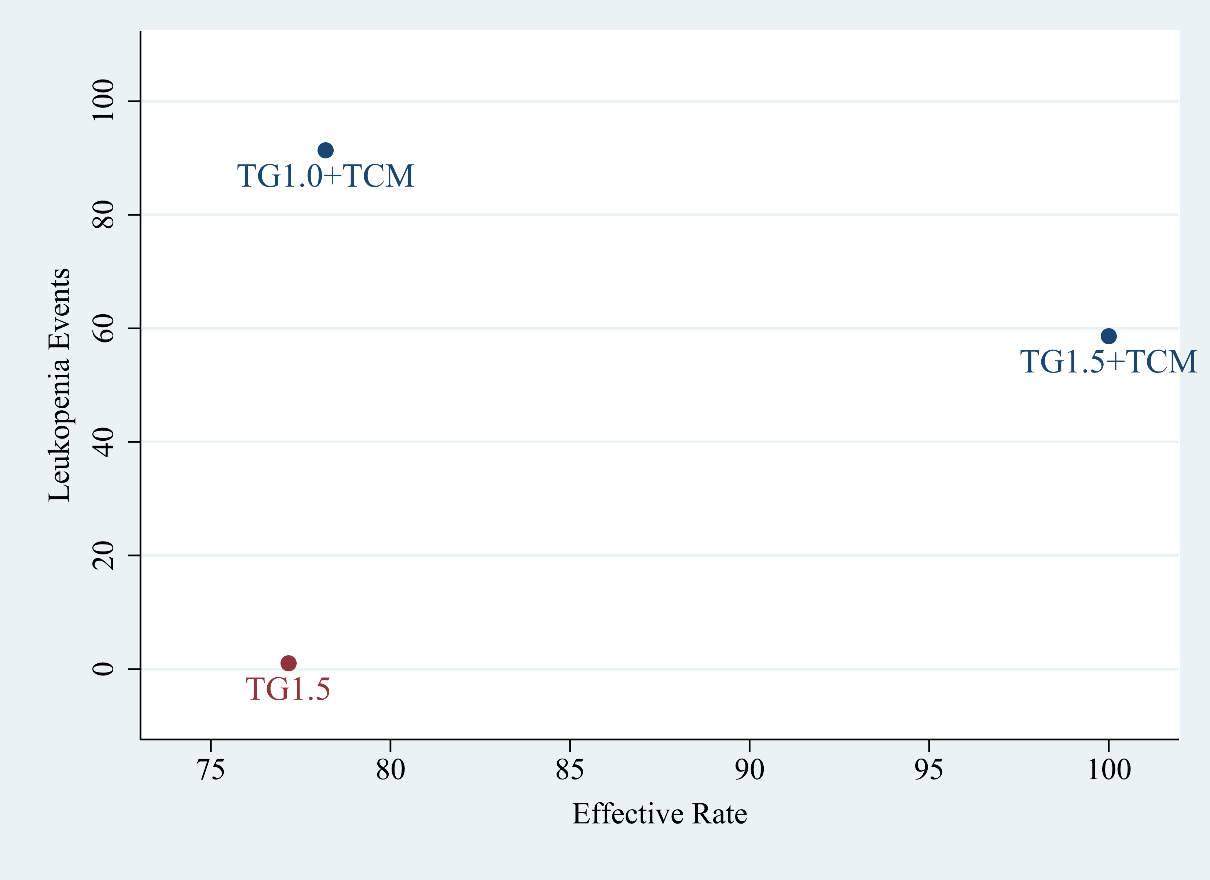


## Fig. S6.3 Effective rate and gastrointestinal events


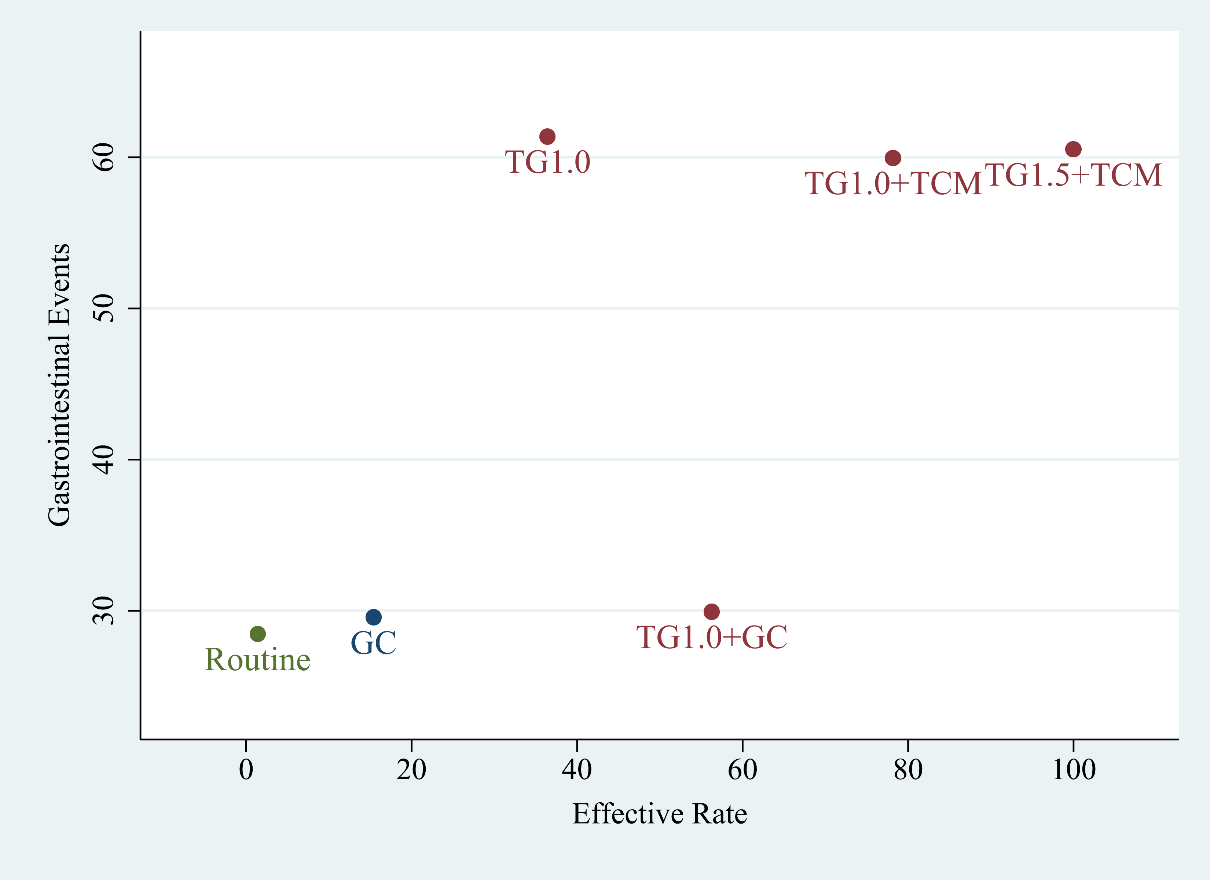

Supplement: Multimedia component 1 [file mmc1.docx]
