# Supplementary material for: Comparison of different doses of Tripterygium glycosides treating in IgA vasculitis nephritis: A Bayesian network meta-analysis
Source: Heliyon. 2024 Jul 14;10(14):e34329. doi: 10.1016/j.heliyon.2024.e34329 (PMC11305250; doi:10.1016/j.heliyon.2024.e34329)
Supplement: Multimedia component 2 [file mmc2.docx]

Appendix A. Supplementary file

Table 1.

Results of PMA (comparison of effective rate)

| Comparison | Number of Studies | Number of Participants | I^2^ (%) | p (I^2^ ) | Effect Estimate  (OR, 95% CI) | p (OR) |
| --- | --- | --- | --- | --- | --- | --- |
| TG1.0 VS RT | 3 | 278 | 0 | 0.69 | 5.95 [2.52, 14.07] | <0.0001 |
| TG1.0 VS GC | 1 | 59 | - | - | 2.85 [0.99, 8.21] | 0.05 |
| TG1.0+GC VS GC | 18 | 1413 | 0 | 1.00 | 5.55[3.85,8.00] | <0.00001 |
| TG1.0+TCM VS TG1.0 | 7 | 486 | 0 | 0.83 | 4.5[2.51,8.07] | <0.00001 |
| TG1.0+TCM VS TCM | 2 | 124 | 0 | 0.81 | 5.03[1.76,14.44] | 0.003 |
| TG1.0+TCM VS TG1.0+GC | 2 | 108 | 0 | 0.34 | 4.01[1.49,10.82] | 0.006 |
| TG1.5+TCM VS TG1.0+TCM | 3 | 164 | 0 | 0.76 | 4.35[1.60,11.79] | 0.004 |
| TG1.5+TCM VS TG1.5 | 7 | 517 | 0 | 0.93 | 5.71[3.01,10.84] | <0.00001 |
| TG1.5+TCM VS TCM | 1 | 24 | - | - | 34.09[1.64,707.92] | 0.02 |

Abbreviations: **TG1.0,** Tripterygium Glycosides (dose: 1 mg/kg/day); **TG1.5,** Tripterygium Glycosides (dose: 1.5 mg/kg/day); **TCM,** traditional Chinese medicine; **GC,** glucocorticoid; **RT,** routine treatment; **OR,** odds ratio; **CI,** confidence interval.

Table 2

Results of PMA (comparison on secondary outcomes (NO. of studies >1))

| Comparison | Number of Studies | Number of Participants | I^2^ (%) | p (I^2^ ) | Effect Estimate  (OR, 95% CI) | p (OR) |
| --- | --- | --- | --- | --- | --- | --- |
| Recurrence rate |  |  |  |  |  |  |
| TG1.0+GC VS GC | 8 | 649 | 0 | 0.94 | 0.17 [0.09, 0.32] | <0.00001 |
| TG1.0+TCM VS TG1.0+GC | 2 | 108 | 0 | 0.95 | 0.15 [0.04, 0.58] | 0.006 |
|  |  |  |  |  |  |  |
| Liver injury events |  |  |  |  |  |  |
| TG1.5+TCM VS TG1.5 | 5 | 401 | 0 | 0.95 | 0.29 [0.09, 0.98] | 0.05 |
| TG1.5+TCM VS TG1.0+TCM | 2 | 140 | 0 | 0.33 | 1.43 [0.44, 4.68] | 0.55 |
| TG1.0+GC VS GC | 3 | 186 | 36 | 0.21 | 0.62 [0.20, 1.92] | 0.40 |
|  |  |  |  |  |  |  |
| Gastrointestinal events |  |  |  |  |  |  |
| TG1.0+GC VS GC | 6 | 436 | 58 | 0.04 | -0.01 [-0.06, 0.04] | 0.59 |
| TG1.0+TCM VS TCM | 2 | 124 | 22 | 0.26 | 0.03 [-0.05, 0.11] | 0.43 |
| TG1.5+TCM VS TG1.0+TCM | 2 | 84 | 0 | 0.39 | 0.02 [-0.06, 0.11] | 0.60 |
| TG1.0 VS RT | 2 | 196 | 10 | 0.29 | -0.05 [-0.11, 0.01] | 0.08 |
| TG1.0+TCM VS TG1.0 | 2 | 160 | 0 | 0.40 | 0.00 [-0.06, 0.06] | 1.00 |
|  |  |  |  |  |  |  |
| Leukopenia events |  |  |  |  |  |  |
| TG1.5+TCM VS TG1.5 | 5 | 401 | 0 | 0.99 | 0.19 [0.05, 0.74] | 0.02 |
| TG1.5+TCM VS TG1.0+TCM | 2 | 140 | 0 | 0.77 | 2.01 [0.51, 7.94] | 0.32 |

Abbreviations: **TG1.0,** Tripterygium Glycosides (dose: 1 mg/kg/day); **TG1.5,** Tripterygium Glycosides (dose: 1.5 mg/kg/day); **TCM,** traditional Chinese medicine; **GC,** glucocorticoid; **RT,** routine treatment; **OR,** odds ratio; **CI,** confidence interval.

Table 3

The league table of two outcomes

| TG1.0 |  |  |  |  |  |  |  |
| --- | --- | --- | --- | --- | --- | --- | --- |
| 6.14  (1.48, 26.66) | TG1.5 | 9.27  (0.83, 129.3) | 71.62  (4.27, 1596) |  |  | 0.48  (0.05, 3.82) | 1.43  (0.14, 17.92) |
| 2.15  (1.06, 4.91) | 0.35  (0.07, 1.53) | TG1.0+GC | 7.44  (1.8, 41.99) |  |  | 0.05  (0, 0.56) | 0.15  (0.07, 0.31) |
| 5.82  (3.32, 10.64) | 0.93  (0.24, 3.52) | 2.76  (1.18, 5.69) | TG1.0+TCM |  |  | 0.01  (0, 0.11) | 0.02  (0, 0.1) |
| 39.52  (11.86, 158.3) | 6.43  (3.4, 13.52) | 17.89  (5.06, 80.59) | 6.75  (2.33, 23.57) | TG1.5+TCM |  |  |  |
| 0.91  (0.17, 4.1) | 0.15  (0.02, 1.03) | 0.42  (0.08, 2.09) | 0.16  (0.03, 0.61) | 0.02  (0, 0.13) | TCM |  |  |
| 0.16  (0.06, 0.37) | 0.03  (0, 0.15) | 0.07  (0.02, 0.22) | 0.03  (0.01, 0.08) | 0  (0, 0.02) | 0.43  (0.13, 1.35) | RT | 3.05  (0.31, 35.69) |
| 0.37  (0.17, 0.8) | 0.06  (0.01, 0.27) | 0.17  (0.11, 0.24) | 0.06  (0.03, 0.15) | 0.01  (0, 0.03) | 0.41  (0.08, 2.43) | 2.34  (0.74, 7.71) | GC |

Note: A summary of the NMA results for the efficacy rate (bottom left) and recurrence rate (top right corner) is shown in Table 3. The row-defining treatment was compared with the column-defining treatment, and the relative effects were measured as risk ratios along with 95%CI. Abbreviations: **TG1.0,** Tripterygium Glycosides (dose: 1 mg/kg/day); **TG1.5,** Tripterygium Glycosides (dose: 1.5 mg/kg/day); **TCM,** traditional Chinese medicine; **GC,** glucocorticoid; **RT,** routine treatment.

Table 4

The surface under the cumulative ranking curve (SUCRA) of each outcome

|  | Effective rate | | Recurrence rate | |
| --- | --- | --- | --- | --- |
| Rank | Treatment | S-V | Treatment | S-V |
| 1 | TG1.5+TCM | 1.000 | TG1.0+TCM | 0.998 |
| 2 | TG1.0+TCM | 0.782 | TG1.0+GC | 0.742 |
| 3 | TG1.5 | 0.772 | GC | 0.371 |
| 4 | TG1.0+GC | 0.563 | TG1.5 | 0.292 |
| 5 | TG1.0 | 0.364 | RT | 0.097 |
| 6 | TCM | 0.351 |  |  |
| 7 | GC | 0.154 |  |  |
| 8 | RT | 0.014 |  |  |

|  | Liver injury events | | Gastrointestinal events | | leukopenia events | |
| --- | --- | --- | --- | --- | --- | --- |
| Rank | Treatment | S-V | Treatment | S-V | Treatment | S-V |
| 1 | TG1.0+TCM | 0.820 | TCM | 0.802 | TG1.0+TCM | 0.914 |
| 2 | RT | 0.709 | TG1.0 | 0.614 | TG1.5+TCM | 0.586 |
| 3 | TG1.5+TCM | 0.676 | TG1.5+TCM | 0.605 | TG1.5 | 0.000(5.250E-05) |
| 4 | TG1.0+GC | 0.482 | TG1.0+TCM | 0.600 |  |  |
| 5 | GC | 0.422 | TG1.0+GC | 0.299 |  |  |
| 6 | TG1.5 | 0.345 | GC | 0.296 |  |  |
| 7 | TG1.0 | 0.046 | RT | 0.285 |  |  |

Abbreviations: **TG1.0,** Tripterygium Glycosides (dose: 1 mg/kg/day); **TG1.5,** Tripterygium Glycosides (dose: 1.5 mg/kg/day); **TCM,** traditional Chinese medicine; **GC,** glucocorticoid; **RT,** routine treatment; **S-V,** SUCRA value.
